# Exploring Long Arm Amide-Linked Side Chains in the Design of Antifungal Azole Inhibitors of Sterol 14α-Demethylase (CYP51)

**DOI:** 10.1021/acs.jmedchem.4c02922

**Published:** 2025-05-22

**Authors:** Marwa Alsulaimany, Mikhail V. Keniya, Rehab S. Alanazi, Yasmeen N. Ruma, Carwyn S. Hughes, Arwyn T. Jones, Joel D. A. Tyndall, Josie E. Parker, Brian C. Monk, Claire Simons

**Affiliations:** † School of Pharmacy and Pharmaceutical Sciences, 2112 Cardiff University, King Edward VII Avenue, Cardiff CF10 3NB, U.K.; ‡ Faculty of Dentistry, Sir John Walsh Research Institute, 2495 University of Otago, Dunedin 9016, New Zealand; § School of Pharmacy, 2495 University of Otago, Dunedin 9054, New Zealand; ∥ School of Biosciences, 2112 Cardiff University, Museum Avenue, Cardiff CF10 3AX, U.K.

## Abstract

The rise in fungal drug resistance has exacerbated the treatment of invasive fungal infections, most commonly caused by Candida. This research describes the synthesis of extended “long-arm” azole antifungals that were evaluated against wild-type and resistant fungal species. Biphenyl derivative **22** was the most effective derivative, displaying potent inhibitory activity against Saccharomyces, Candida, and Cryptococcus CYP51 enzymes, including in resistant strains, in comparison with posaconazole. The X-ray crystal structure of *S*-**22** complexed with *S. cerevisiae* CYP51 showed a hydrogen bond between the oxygen of the trifluoromethoxy group of *S-* **22** and the His381 side chain of *S. cerevisiae* CYP51, which is postulated to contribute significantly to its binding, and stabilization in the presence of the *S. cerevisiae* CYP51 Y140F/H, C. parapsilosis and C. auris CYP51 Y132F mutations and the C. auris K143R mutation. Computational studies and IC_50_ evaluation of compound **22** vs *C. albicans* wild-type, Y132F, and Y132H/K143 mutant strains supported MIC observations.

## Graphical abstract

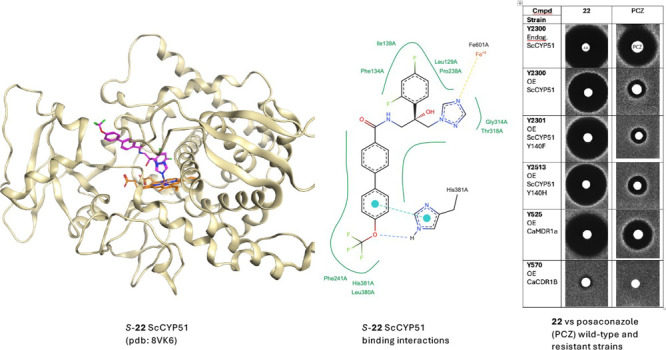

## Introduction

The global threat of invasive fungal infection (IFI) and antifungal resistance led the WHO to list *Candida albicans*, Candida auris, Aspergillus fumigatus, and *Cryptococcus neoformans* as critical fungal pathogens.^1^ IFIs mainly affect people with health conditions associated with impaired immune function, including those with cancer, HIV, TB, and diabetes mellitus, as well as immunocompromised patients such as transplant patients and those in intensive care units.^2–7^ IFIs were also highlighted during the COVID pandemic, where candidemia, aspergillosis, and mucormycosis were common comorbidities.^8^

Four classes of antifungal agents are used to treat IFIs^9^: azole antifungals (e.g., fluconazole, voriconazole, and posaconazole), echinocandins (e.g., caspofungin, micafungin, and the recently approved rezafungin^10^), polyenes (e.g., amphotericin B), and pyrimidines (e.g., flucytosine). Azoles, which inhibit ergosterol biosynthesis by targeting sterol 14α-demethylase (CYP51), have been a mainstay of antifungal therapy.

The major role of fungal CYP51 is the demethylation of lanosterol, a key intermediate in the biosynthesis of ergosterol.^11^ Ergosterol, the major sterol component of fungal plasma membranes, is essential for membrane stability, with inhibition of fungal CYP51 resulting in loss of ergosterol and accumulation of other sterol intermediates affecting fungal membrane stability and fungal growth.^11^ However, resistance to azole antifungal, resulting from overexpression and/or mutations in the *ERG11* (*CYP51*) gene^12,13^ or overexpression of plasma membrane efflux pumps,^14^ can result in reduced or complete loss of efficacy. Fluconazole (FLC) and voriconazole (VCZ) (Figure 1) are particularly affected by single and double amino acid mutations in CYP51, as demonstrated by *C. albicans* CYP51 (CaCYP51)^12^ with mutations of Tyr132 (Y132H or Y132F) and Lys143 (K143R) significantly reducing antifungal activity. The long-chain triazole derivatives itraconazole (ITC) and posaconazole (PCZ) are used in the treatment of Candida and Aspergillus IFIs. However, ITC is less well tolerated compared with the smaller azoles (FLC and VCZ) and PCZ, the most potent azole antifungal (*C. albicans* MIC ≤ 0.03 μg/mL^11^), which has been shown to retain efficacy against FLC-resistant strains,^12^ has limited bioavailability.^15,16^ Oteseconazole (VT-1161) (Figure 1), the first tetrazole antifungal agent approved for the treatment of recurrent vulvovaginal candidiasis,^17^ has improved selectivity, however, azole resistance owing to drug efflux and CYP51 mutations reduces its antifungal activity.^18^

**Figure 1:**
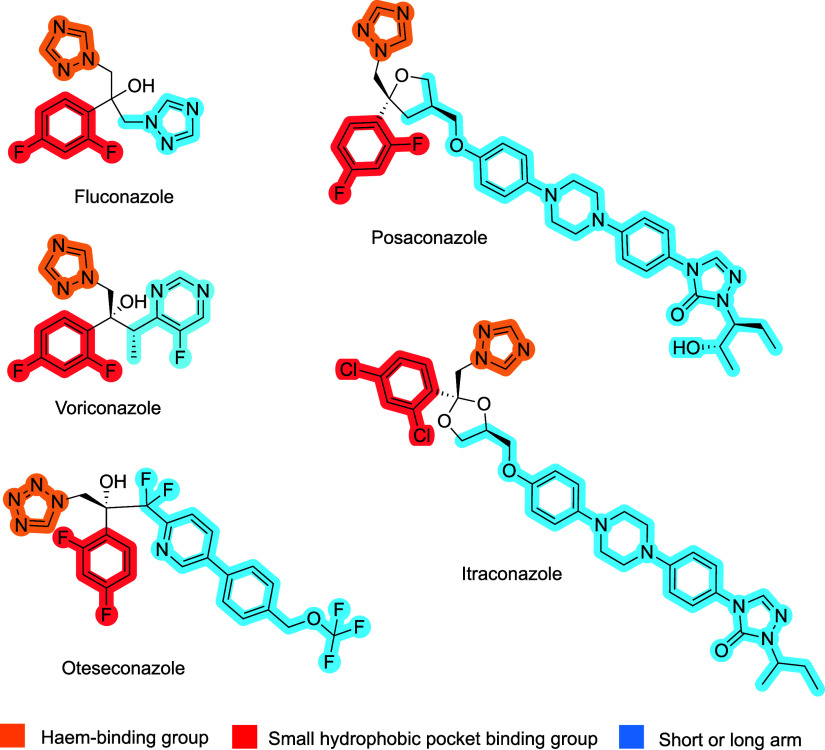
Clinically used azole triazole and tetrazole antifungal agents.

Drug resistance and limitations of the currently available azole antifungals has resulted in the design of new azoles, with some interesting hybrid combinations reported^19^ such as FLC-ketoconazole hybrid (*C. albicans* SC5314, MIC_50_ < 1 μg/mL),^20^ FLC–COX inhibitor hybrid (*C. albicans* drug susceptible strains MIC_80_ 0.003–0.007 μg/mL)^21^ and, with the discovery of oteseconazole, there is interest in tetrazole derivatives.^22,23^ Although promising azole antifungals have been described, reported antifungal data are generally limited to drug-sensitive fungal strains. In the research described here, we have considered the spectrum of activity against different fungal species and, importantly, activity against resistant strains, as well as selectivity, toxicity, and drug-like properties.

A range of triazole derivatives with long arm amide-linked extensions have been designed to investigate their effect on CYP51 binding (Figure 2). We have previously investigated the linker and found the amide optimal compared with urea, thiourea, and sulfonamide groups.^24^ By maintaining a 2,4-difluorophenyl and 1,2,4-triazol-1-yl head group, with the triazole as the haem binding group and the amide as the linker, the optimal combination of “long arm” extension has been explored through evaluation against a wide range of fungi, including *S. cerevisiae* strains individual recombinant wild-type and azole-resistant CYP51 enzymes, or the MDR1 or CDR1 drug efflux, complemented by studies in both wild-type and azole-resistant clinical isolates of pathogenic fungi.

**Figure 2:**
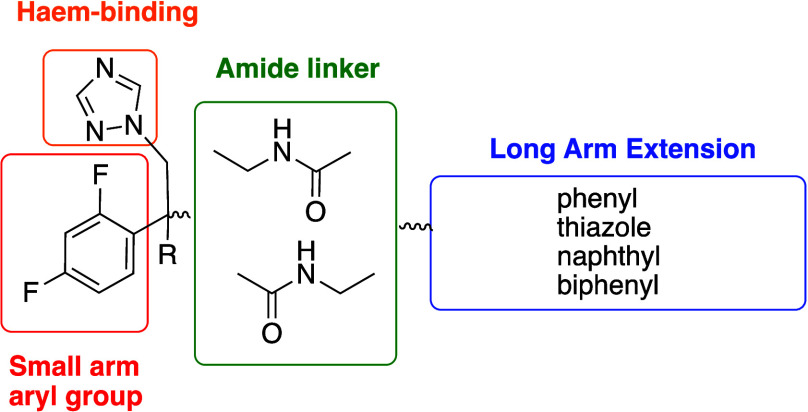
Design of triazole antifungal agents with long arm amide-linked extensions. (R = OH or H).

The importance of the tertiary hydroxyl group (Figure 2, R = OH), which has been shown to be involved in a water-mediated hydrogen bonding network for FLC, VCZ and VT-1161 with Tyr140 in *S. cerevisiae* ^18,25^ and for FLC with Tyr132 in *C. albicans*,^12,24^ was also investigated by the preparation and testing of compounds lacking the hydroxyl group (Figure 2, R = H).

## Results and Discussion

### Chemistry

Starting with 1-(2,4-difluorophenyl)-2-(1*H*-1,2,4-triazol-1-yl)­ethan-1-one (**1**), the key amine intermediate (**4**) was prepared in a 3-step synthetic route as previously described (Scheme 1).^26,27^ The first step involved a Corey–Chaykovsky epoxidation^28^ of the ketone in **1** by reaction with tetramethylsulfoxonium iodide (TMSOI) under basic conditions using 20% aqueous NaOH at 60 °C for 6 h to give **2**.^29^ Ring opening of the epoxide (**2**) with NaN_3_ gave the azide (**3**), which was reduced to the required amine intermediate (**4**) by catalytic hydrogenation using Pd/C at ∼40 psi (Paar hydrogenator). Coupling of the amine (**4**) with the respective carboxylic acids (**5**, **6**, **8**–**10**), using carbonyldiimidazole (CDI) as the activating/coupling reagent, or acid chloride (**7**), gave the final amide products (**11**–**16**) in good yields (54–65%) (Scheme 1).

**Scheme 1:**
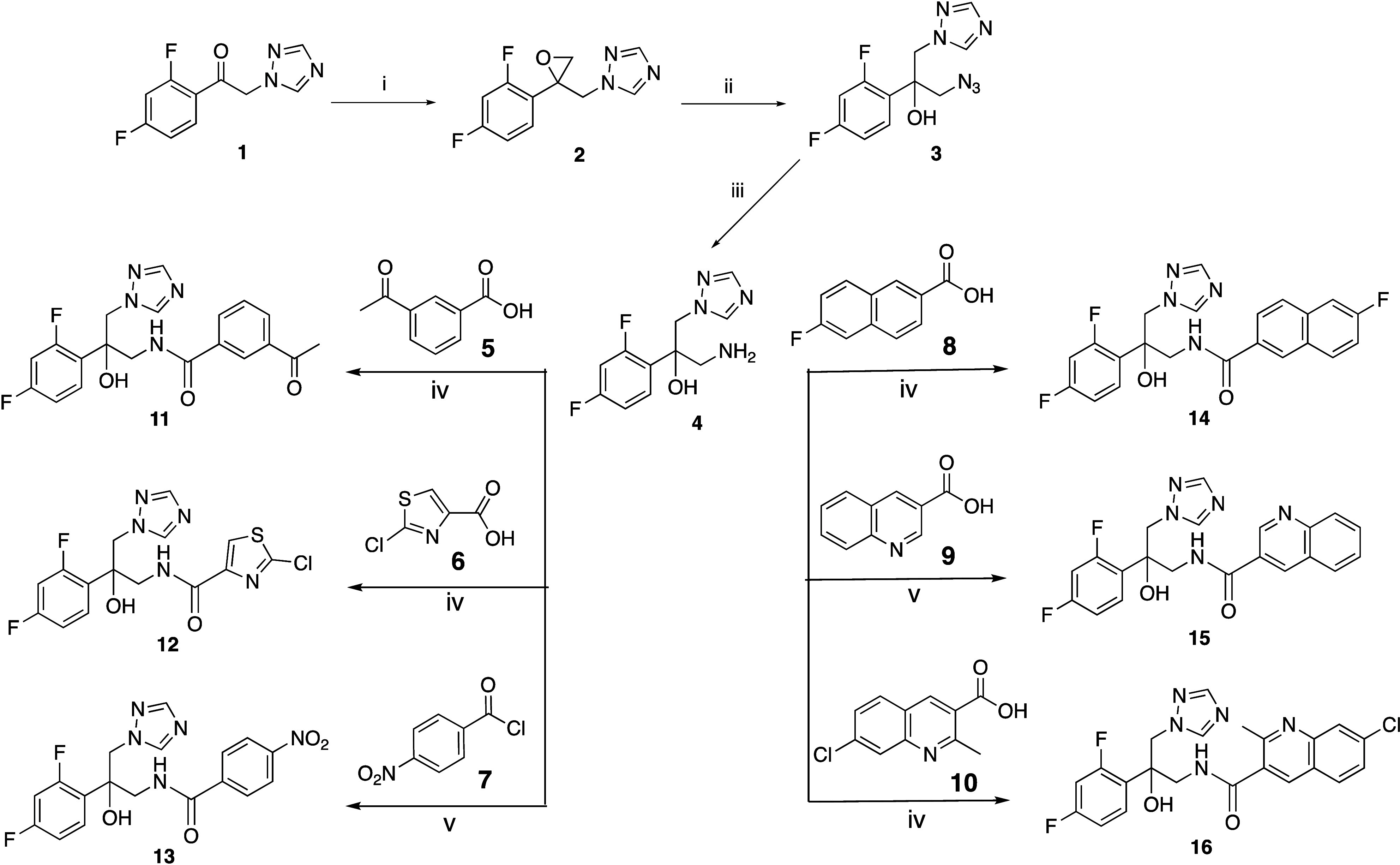
*Reagents and Conditions*: (i) TMSOI, 2M Aq. NaOH, Toluene, 60 °C, 6 h, 83% Crude (ii) NaN_3_, NH_4_Cl, DMF, 60 °C, 2 h, r.t., 60% (iii) H_2_, Pd/C, EtOH, ∼40 psi, 5 h, 69% (iv) CDI, DMF, r.t., o/n 54–65% (v) Pyridine, r.t., o/n, 65%

The biphenyl derivatives (**21**–**24**) were prepared by CDI coupling of the amine intermediate (**4**) with the biphenylcarboxylic acid derivatives (**17**–**20**), while the extended diamide derivative (**26**) was prepared by reaction of the amine (**25**), obtained by catalytic reduction of the nitro compound (**13**), with 4-chlorobenzoyl chloride (Scheme 2).

**Scheme 2:**
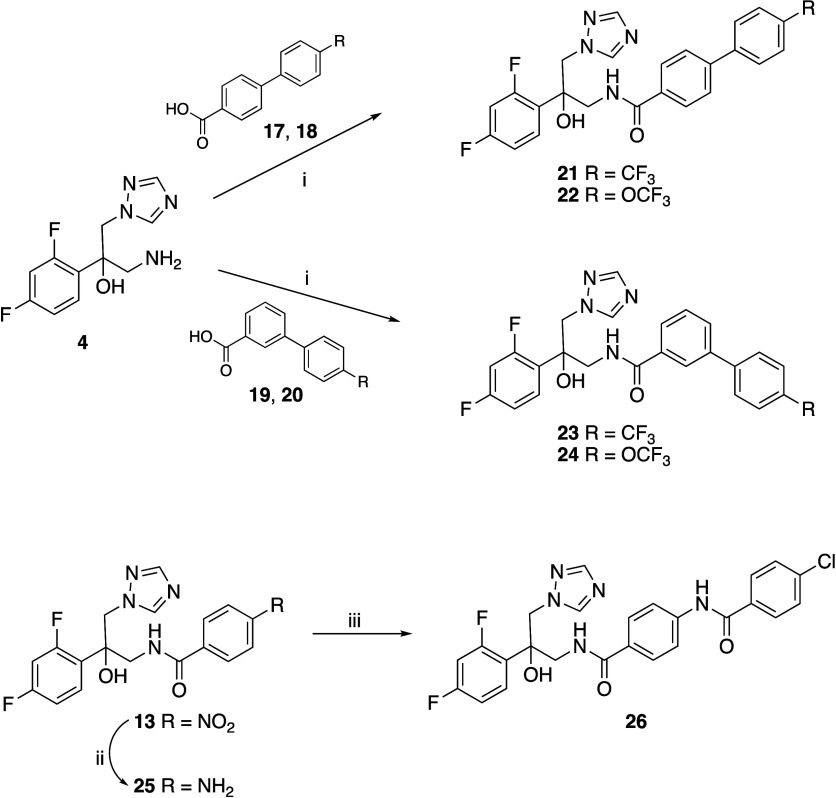
*Reagents and Conditions*: (i) CDI, DMF, r.t., o/n 65–76% (ii) H_2_ (Balloon), Pd/C, EtOH, r.t., 3 h, 100% (iii) 4-Chlorobenzoyl Chloride, Pyridine, r.t., o/n, 78%

To investigate the importance of the tertiary hydroxy group for antifungal activity in wild-type and resistant fungal strains, a different synthetic strategy was used to prepare the biphenyl (**37**–**40**) (Scheme 3) and extended diamide (**44**–**48**) (Scheme 4) derivatives. The triazole carboxylic intermediate (**32**) was prepared in 5 steps from 2-(2,4-difluorophenyl)­acetic acid (**27**). The first step converted the acid (**27**) to the methyl ester (**28**) using SOCl_2_/CH_3_OH to allow subsequent selective deprotonation of the acidic α-proton of (**28**) using NaOCH_3_ and reaction of the resulting anion with paraformaldehyde to give the hydroxy derivative (**29**). The hydroxyl group was then converted to the mesylate (**30**), which was displaced by a triazole anion, generated *in situ* by the reaction of triazole with K_2_CO_3_. The methyl ester of the triazole derivative (**31**) was then hydrolyzed under basic conditions to give the key carboxylic acid intermediate (**32**). CDI coupling with biphenyl amine derivatives (**33**–**36**) gave the final amide products (**37**–**40**) in yields ranging from 40 to 66% (Scheme 3).

**Scheme 3:**
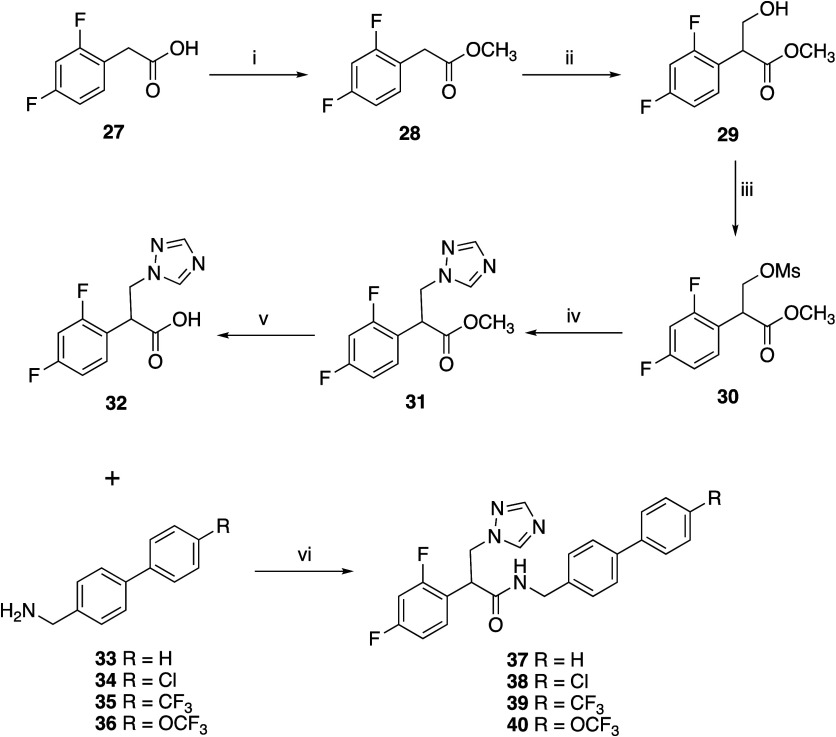
*Reagents and Conditions*: (i) SOCl_2_, MeOH, 60 °C, 3 h, 91%, (ii) NaOCH_3_, (HCHO)_*n*_, DMSO, r.t., 4 h, 78%, (iii) MsCl, Et_3_N, CH_2_Cl_2_, r.t., o/n, 84%, (iv) (a) Triazole, K_2_CO_3_, CH_3_CN, 45 °C, 1 h (b) **30**, 70 °C, 4 h Then r.t. o/n, 85% (v) LiOH, THF, H_2_O, r.t., 1 h, 73% (vi) CDI, DMF, r.t., o/n 40–66%

**Scheme 4:**
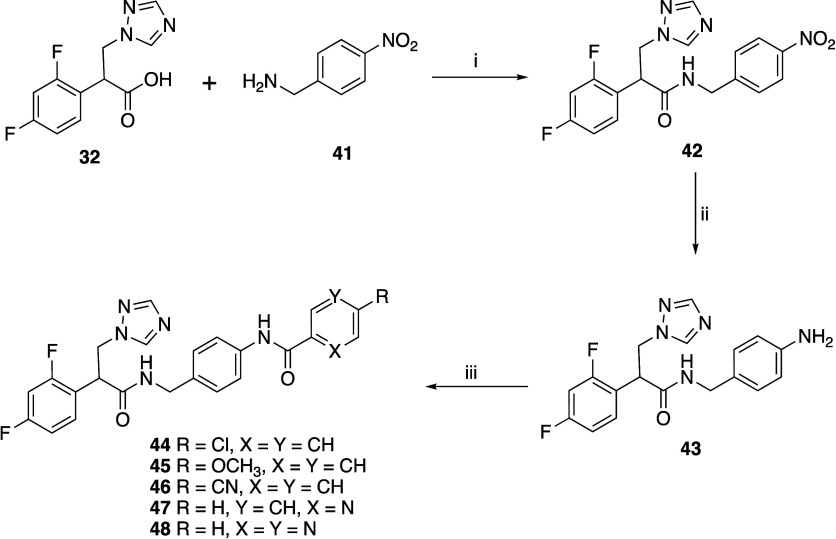
*Reagents and Conditions*: (i) B­(OCH_2_CF_3_)_3_, CPME, 100 °C, o/n 67% (ii) H_2_ (Balloon), Pd/C, CH_3_OH, r.t., 3 h, 93% (iii) Acyl Chloride, Pyridine, r.t., o/n, 68–80%

The extended diamide derivatives (**44**–**48**) were prepared in three steps starting from the coupling of the triazole carboxylic acid intermediate (**32**) with 4-nitrobenzylamine (**41**) (Scheme 4). An initial coupling reaction using CDI, as in Schemes 1–3, was unsuccessful and gave complex mixtures. However, use of B­(OCH_2_CF_3_)_3_ as the coupling reagent and cyclopentylmethyl ether (CPME) as solvent^30^ at 100 °C overnight produced the nitro derivative (**42**) in 67% yield. Reduction of the nitro group via catalytic hydrogenation yielded the amine intermediate (**43**), which was reacted overnight with the respective acyl chloride in pyridine to give the final products (**44–48**) (Scheme 4).

### Antifungal Evaluation

#### Susceptibility Tests with Recombinant S. cerevisiae Strains Expressing Fungal CYP51 or Drug Efflux Pumps

Final compounds were screened as racemic mixtures using agarose diffusion assays to visualize their inhibitory activity against a panel of recombinant *S. cerevisiae* strains i.e., *S. cerevisiae* strains expressing control levels of wild-type CYP51 (ScCYP51), overexpressed wild-type ScCYP51, overexpressed azole-resistant ScCYP51 Y140F/H mutations and overexpressed *C. albicans* efflux pumps MDR1a or CDR1B (Figure 3 and Table S1). The antifungal azole PCZ was used as a positive control, and glucan synthase inhibitor micafungin (MCF) served as an independent control.

**Figure 3:**
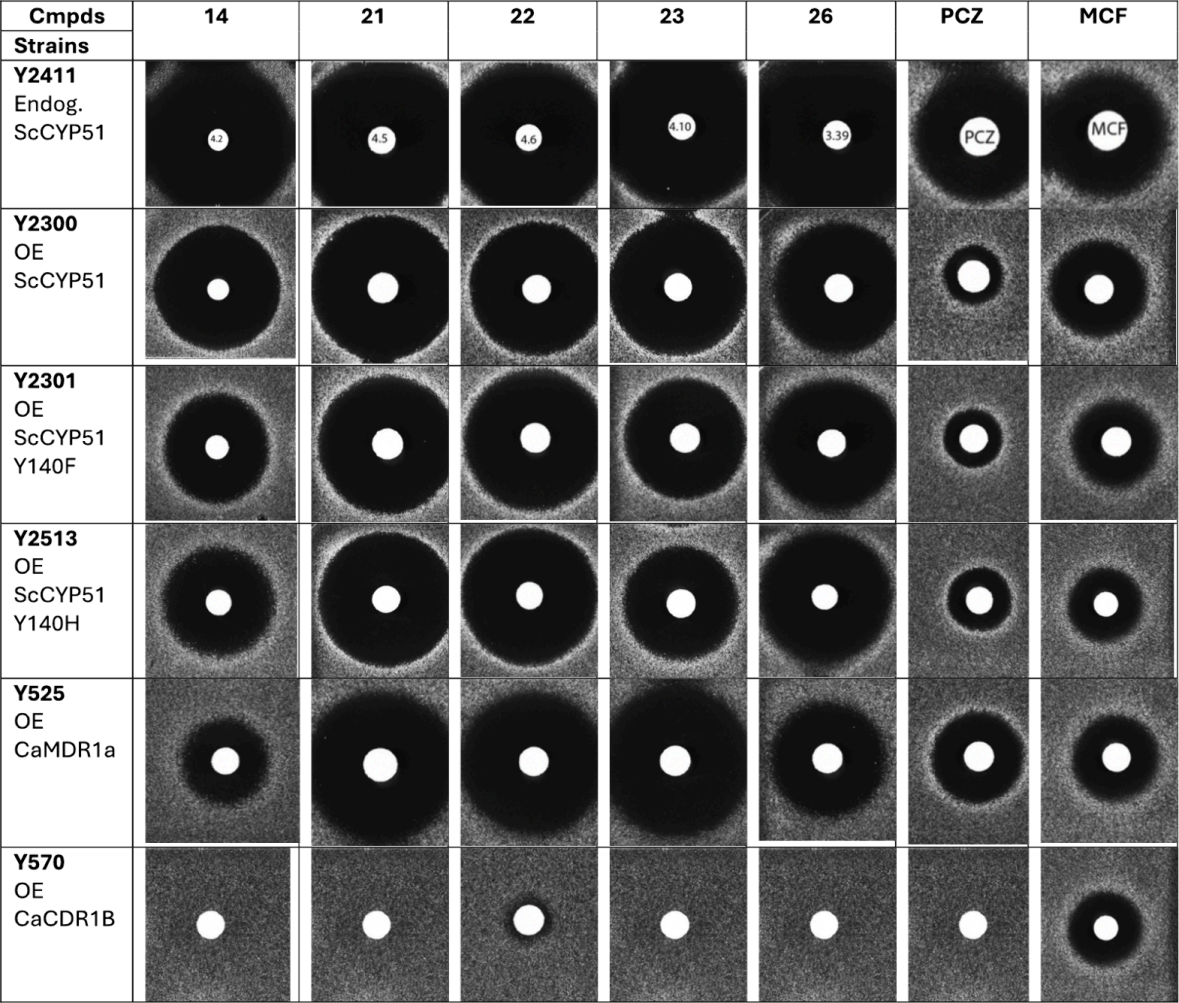
Susceptibilities of yeast constructs to exemplar compounds **14**, **21**, **22, 23,** and **26.** Y2411–azole sensitive control strain, Y2300 ScCYP51 overexpressed, Y2301 ScCYP51 Y140F mutant, Y2513 ScCYP51 Y140H mutant, Y525 MFS efflux pump CaMDR1a overexpressed, and Y570 ABC efflux pump CaCDR1B overexpressed. PCZ Posaconazole, MCF Micafungin.

Evaluation of the shorter aryl amide derivatives (**11**–**16**) (Figures 3 and S1) found that all compounds were active against the hypersusceptible *S. cerevisiae* control strain Y2411 and had reduced activity against the Y2300 strain overexpressing functional ScCYP51, consistent with targeting of ScCYP51. Of these compounds, only the 6-fluoro-2-naphthamide derivative (**14**) and the quinoline-3-carboxamide (**15**) derivative showed activity against the azole-resistant strains expressing ScCYP51 Y140F/H. The 6-fluoro-2-naphthamide derivative (**14**) was the optimal compound from this series. Still, it had a reduced effect against strain Y525, which overexpresses the *C. albicans* MFS transporter MDR1a, and no effect against strain Y570, which overexpresses the *C. albicans* ABC transporter CDR1B (Figure 3). The biphenyl derivatives (**21**–**24**) displayed effective inhibitory activity against all the strains except Y570 (Figures S2 and 3) with the 4’-(trifluoromethyl)-[1,1’-biphenyl]-4-carboxamide (**21**) and 4’-(trifluoromethoxy)-[1,1’-biphenyl]-4-carboxamide (**22**) derivatives showing optimal inhibitory activity (Figures 3 and S2). The extended diamide (**26**) and the biphenyl (**22**) showed similar activity against ScCYP51 and its mutants, but the diamide was less effective when CaMDR1a was expressed (Figures S2 and 3). In response to the *para*-position of the amide attachment on the biphenyl group, the ScCYP51 Y132F/H mutations conferred significantly greater resistance to **23** and **24** than **21** and **22** (Figures 3 and S2). The biphenyl derivatives lacking the tertiary hydroxy group (**37**–**40**) all showed inhibitory activity against the susceptible *S. cerevisiae* control strain Y2411 and the CYP51 overexpressing *S. cerevisiae* strain Y2300 (Figure S3). The trifluoromethyl derivative (**39**) lacked inhibitory activity against the ScCYP51 Y140 mutants (Y2301 and Y2513) and MFS transporter overexpressing (Y520) strains, while the other derivatives (**37**, **38**, and **40**) showed low inhibitory activity (Figure S3).

The extended diamide derivatives lacking a hydroxy group (**44**–**48**) showed mixed results (Figure S4). The benzamide derivatives (**44**–**46**) displayed positive results against Y2411 and Y2300, while the pyrazine-2-carboxamide (**48**) had lower inhibitory activity against the Y2300 strain, and the nicotinamide derivative (**47**) poorly inhibited Y2411 and did not inhibit Y2300 (Figure S4). The most promising derivative in this series was chlorobenzamide (**44**), which strongly inhibited the susceptible control strain Y2411 and the ScCYP51 overexpressing strain Y2300 (Figure S4) and retained some inhibitory activity against the CaMDR1a but not the CaCDR1B expressing strain. Despite the lack of the tertiary hydroxyl group, it showed reduced activity against the ScCYP51 Y140F/H mutants compared with **21**, **22**, and **23**, which retain the tertiary hydroxyl group.

Minimum inhibitory concentrations (MIC_80_) values were obtained for recombinant CYP51s expressed in a hypersusceptible *S. cerevisiae* host strain in response to the more promising compounds **14**, **22, 23, 24**, and **26** (Table 1). MIC values were also obtained for *S. cerevisiae* strains expressing *S. cerevisiae* CYP51 Y140F/H and for clinically relevant MFS (MDR1a) and ABC (CDR1B) transporters from *C. albicans* expressed in *S. cerevisiae* (Table S1).

**Table 1:** Susceptibility of *S. cerevisiae* Strains Expressing Recombinant *S. cerevisiae* or *C. albicans* Proteins Plus *C. albicans* Clinical Isolates^a^ ^,^ ^b^

compound	14	22	23	24	26	PCZ
strain	MIC_80_ (nM)					
*S. cerevisiae* models						
Y2411 Endog. ScCYP51	15 ± 3	30 ± 23	37 ± 11	30	39 ± 14	97 ± 40
Y2300 OE ScCYP51	48 ± 4	114 ± 14	69 ± 39	163 ± 61	182 ± 30	2004 ± 50
Y2301 OE ScCYP51 Y140F	326 ± 48	93 ± 46	411 ± 71	419 ± 83	123 ± 38	191 ± 41
Y2513 OE ScCYP51 Y140H	632 ± 123	78 ± 56	805 ± 121	719 ± 1	87 ± 4	147 ± 57
*C. albicans* models						
Y2459 OE CaCYP51 CaCpr	54 ± 21	54 ± 22	60 ± 11	116 ± 61	118 ± 3	220 ± 70
Y525 OE CaMDR1a	≫1000	451	440	430	>1000	218
Y570 OE CaCDR1B	33,500	5800	8100	17,000	>40,000	>25,000
*C. albicans* clinical isolates						
Y71 SC5314	18	40	100	85	208 ± 83	73
Y1	10	67	120	120	285	70
Y610 FHB1	75	153 ± 67	400 ± 113	370 ± 42	950	151 ± 16
Y611 FHB3	2400	1650	4250	3400	19,500	530

a The endogenous ScCYP51 is deleted in all strains expressing recombinant CYP51s but not in strains expressing recombinant drug efflux pumps. OE indicates overexpression of following proteins.

b Values are presented as mean ± SD based on at least two separate experiments, with each experiment providing the mean of a pair of measurements (at least 4 measurements in total). When no SD is given, the numbers represent screens that present the mean of a pair of measurements.

All the compounds tested affected the control strain Y2411 expressing the endogenous ScCYP51 at MIC_80_ values of <50 nM. A further 2- to 5-fold reduction in susceptibility was found with the strain overexpressing recombinant ScCYP51, consistent with targeting of this enzyme. As each of the test compounds contains a tertiary hydroxyl, comparable with FLC and VCZ but not PCZ, further reductions in susceptibility were expected for strains expressing the ScCYP51 Y140F/H mutations. Such reductions in susceptibility were observed in response to **14**, **23**, and **24**, but not **22** or **26**. As with other azole drugs, the dramatic reductions in susceptibility showed the test compounds were effluxed by strains expressing CaCDR1B and, in common with other azoles, except the long-tailed azoles such as PCZ and ITC, they were also effluxed by the strain expressing CaMDR1a. The selected compounds showed good activity against wild-type *C. albicans* clinical isolates comparable with PCZ but not strains known to overexpress CaCDR1 drug efflux pumps (Table 1). *C. albicans* strain Y610 weakly expresses CaCDR1, and its daughter strain Y611 overexpresses this efflux pump, explaining reductions in susceptibility by test compounds and PCZ for these strains compared with the wild type Y1 and Y71 strains (Table 1).

### Structural Resolution of ScCYP51 in Complex with Compound **22**

The X-ray crystal structure of ScCYP51–6× His in complex with *S*-**22** (PDB ID: 8VK6) was determined at a resolution of 1.89 Å via molecular replacement (Table S2), as with previously described structures.^18,25^ The crystallization experiment used a racemic mixture of **22**. Clear electron density was present for only the *S*-enantiomer of the ligand (Figure S5). The ligand coordinates the haem iron via the triazole nitrogen (Figure 4a). A water-mediated hydrogen bond network is present between a haem propionate, Tyr140, the tertiary hydroxyl, and the linker amide NH of *S*-**22**. A further hydrogen bond is made between the terminal trifluoromethoxy group and the imidazole of His381. Another water molecule is in close proximity to the outer aromatic ring, making hydrogen bonds with three main chain atoms (His381 NH, Ser382 NH, and C=O). The biphenyl group makes van der Waals interactions with Leu129, Leu380, Phe384, and Met509.

**Figure 4:**
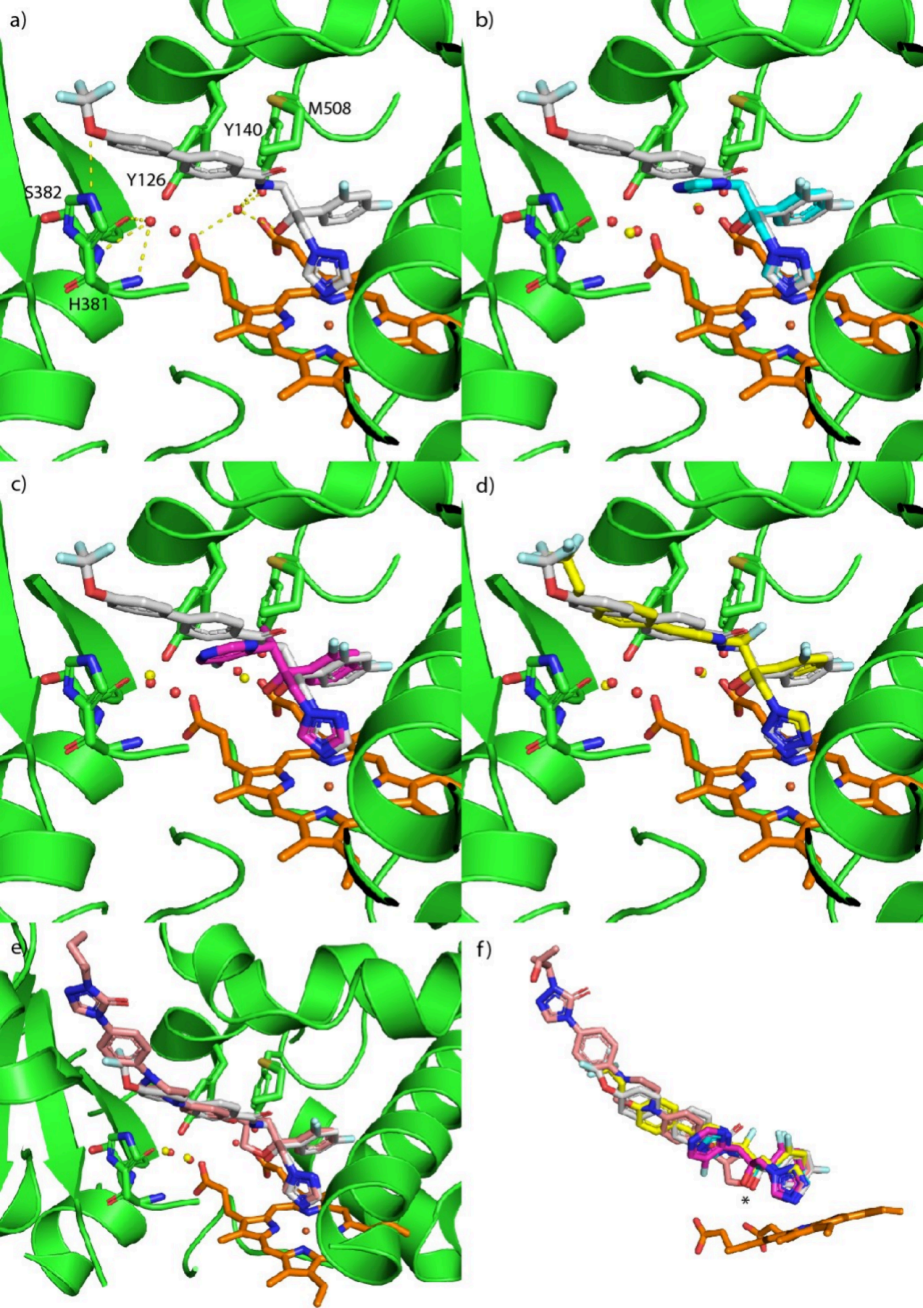
Binding of the Azole Antifungals to ScCYP51. (a) *S*-**22** (white) bound in the active site of the enzyme (green cartoon, parts of the protein are hidden to aid visualization) (PDB ID: 8VK6). Water molecules are shown as red spheres, haem and the iron are shown as orange sticks. Hydrogen bonds are shown as dashed yellow lines. (b) FLC (cyan) complexed with ScCYP51 (PDB ID: 4WMZ)^31^ superimposed on the *S*-**22** complex. Conserved waters from the FLC structure are shown as yellow spheres. (c) VCZ (magenta) (PDB ID: 5HS1)^25^ superimposed on the *S*-**22** complex. (d) Oteseconazole (yellow) (PDB ID: 5UL0)^18^ superimposed on the *S*-**22** complex. (e) PCZ (salmon) (PDB ID: 6E8Q)^18^ superimposed on the *S*-**22** complex. (f) All 5 ligands in their ScCYP51-bound conformation. The four hydroxyl groups of *S*-**22**, FLC, VCZ, and oteseconazole along with the PCZ tetrahydrofuran oxygen are indicated with an asterisk.

Compound *S*-**22** binds in the same position and orientation as other known short-tail azole drugs FLC and VCZ, i.e., with the same water-mediated hydrogen bond network but with the additional hydrogen bond to the amide linker (Figure 4b,c). The additional water molecule near His381 is conserved in these structures. Compound *S*-**22** is most similar to oteseconazole in shape, with the biphenyl rings overlapping in a staggered fashion as the amide linker is one atom longer than the linker in oteseconazole (Figure 4d). As a result, the terminal trifluoromethyl groups of oteseconazole and compound *S*-**22** are in similar positions. The coordinating triazole and difluorophenyl group of the long chain azole PCZ binds in an almost identical manner to *S*-**22,** with the 4-ring chain extending further along the access channel (Figure 4e). The piperazine ring of PCZ also forms a hydrogen bond with conserved water adjacent to His381. The tertiary hydroxyl group of *S*-**22**, FLC, VCZ, oteseconazole, and the oxygen of the 5-membered tetrahydrofuran ring of PCZ are all in the same position, adjacent to the haem (Figure 4f).

### In Vitro Inhibition of CYP51 by Compound **22**

The *S. cerevisiae* CYP51 single amino acid mutants Y140F/H (Y132 in *C. albicans*) within the ligand binding site did not reduce the antifungal activity of lead compound **22** (Table 1, Figure 3), i.e., a behavior similar to PCZ but unlike FLC, VCZ, and oteseconazole. The CaCYP51 K143R mutation also affects binding with a haem propionate, which may distort the haem and helix C, and therefore has the potential to affect azole binding. Although single mutations can affect antifungal activity, the greatest effect has been observed with the double mutants.^12^

IC_50_ values were determined for the lead compound **22** with the isolated wild-type CaCYP51, single mutant (Y132F), and the double mutant (Y132H + K143R) enzymes (Table 2 and Figure S6). Compound **22** inhibited the wild-type CaCYP51 with an IC_50_ of 0.6691 ± 0.02115 μM and retained inhibitory activity against the single Y132F mutant (IC_50_ 1.238 ± 0.0379 μM). However, this inhibitory activity was notably reduced against the double mutant (IC_50_ = 8.490 ± 1.271 μM). A similar profile against wild type and double mutant was noted for FLC, while PCZ retained inhibitory activity against both strains.^12^

**Table 2:** IC_50_ Values against Wild Type and Double Mutant CaCYP51

	IC_50_ ^a^ (μM)		
CaCYP51	22	FLC	PCZ^b^
Wild type	0.669 ± 0.0212	0.335 ± 0.017	0.195 ± 0.009^b^
Y132F	1.238 ± 0.0379	0.606 ± 0.066^b^	0.343 ± 0.020^b^
Y132H + K143R	8.490 ± 1.271	25.72 ± 3.550	0.196 ± 0.007^b^

a IC_50_ determinations were performed in duplicate. Mean IC_50_ values together with standard deviations are shown.

b Ref 12.

### Selectivity of Compound **22**

Lead compound **22** showed good (13 to 37-fold) selectivity for CaCYP51 compared with the human liver drug metabolizing enzymes 1A2, 2C9, 2C19, and 2D6. Only modest selectivity was observed for the dominant liver drug metabolizing enzyme CYP3A4 (Table 3, IC_50_ 1.62 ± 0.23 μM), which is consistent with CYP3A4 inhibition exhibited by most azole antifungals^32–34^ (e.g., PCZ, IC_50_ 2.4 ± 0.4 μM^32^; ITC 0.03–0.13 μM^33,34^ ; VCZ, IC_50_ 2.90–3.80 μM^33,35^ ), with fluconazole (IC_50_ 6–13.1 μM^33,34^ ) and the tetrazole oteseconazole (IC_50_ 140 μM^33^) exceptions.

**Table 3:** CYP IC_50_ (μM) Profile of Lead Compound **22** ^a^

compound	1A2	2C9	2C19	2D6	3A4	CaCYP51
**22**	9.33 ± 1.39	>25	14.5 ± 2.23	>25	1.62 ± 0.23	0.669 ± 0.021

a Control standards: CYP1A2 a-naphthoflavone IC_50_ 0.02 ± 0.002 μM, CYP2C9 sulfaphenazole IC_50_ 0.245 ± 0.05 μM, CYP2C19 tranylcypromine IC_50_ 14.4 ± 1.62 μM, CYP2D6 quinidine IC_50_ 0.137 ± 0.015 μM, CYP3A4 ketoconazole IC_50_ 0.076 ± 0.002 μM.

The modest selectivity for human CYP3A4 observed for the long-arm azoles, including compound **22**, is primarily owing to the large active site of human CYP3A4. CY3A4 is responsible for the metabolism of ∼50% of drugs and has been shown to be highly flexible with ligand-induced conformational changes.^36^ CYP3A4 has also been shown to accommodate multiple ligands.^36^ Therefore, although the long-arm azoles (including **22**) are large and quite bulky, which helps to exclude these compounds from binding with the CYP enzymes with smaller active sites, they are still accommodated by CYP3A4. However, this does not explain the reported CYP3A4 selectivity of oteseconazole,^33^ which *S*-**22** most closely fits from superimposition (Figure 4d), so difficult to rationalize the difference observed in CYP3A4 selectivity either by crystallography or computationally. A better rationale may be related to the tetrazole moiety, which has reduced basicity compared with triazole, so it may not bind as strongly with the CYP3A4 haem iron.

### Viability Assay of Compound **22**

The cytotoxic effect of lead compound **22** was evaluated using a viability assay with the human normal breast epithelial cell line MCF-10A,^37^ compared with PCZ as the gold standard and staurosporine as a positive toxic control. MCF-10A cells were incubated for 48 h with compound **22** and PCZ (concentration range of 10 μM to 1 pM) and staurosporine (10 μM) before performing the CellTiter Blue assay.^38^ Compound **22** was very well tolerated with no difference observed between **22** and PCZ (Figure 5).

**Figure 5:**
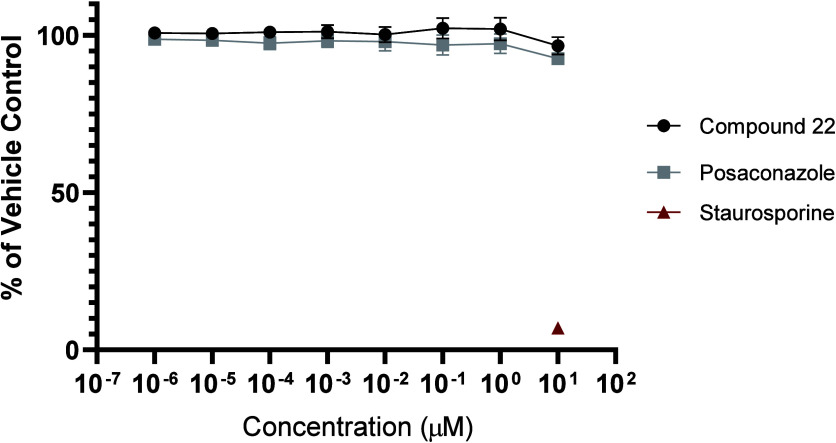
Viability analysis of compound **22** and Posaconazole incubated with MCF-10A cells for 48 h with staurosporine acting as a toxic control. Error bars represent SEM from three independent experiments.

### Computational Analysis of *C. albicans* Wild-Type CYP51 and the Single Mutants (Y132F, Y132H, and K143R) and Double Mutant (Y132H + K143R)

CaCYP51 wild type, single mutants (Y132F, Y132H and K143R) and Y132H + K143R double mutant protein–ligand complexes were prepared as previously described using the crystal structure of CaCYP51 (PDB 5FSA)^24^ and subject to molecular dynamics simulations using the Desmond program of Schrödinger software (full details provided in the Supporting Information).^39,40^ The single and double mutants were prepared using the protein builder function in the molecular operating environment software^41^ to mutate Y132 and K143, followed by energy minimization of the side chains prior to molecular dynamics simulation of the protein *S*-**22** complex. Attempts to perform MD simulation on the Y132H + K143R double mutant protein alone resulted in considerable distortion of the haem with the Fe pulled out of the expected planar conformation and sitting below the protoporphyrin ring, with subsequent loss of two hydrogen bonds in the protoporphyrin ring (Figure S7). For this reason, the protein *S*-**22** complex was directly subject to MD simulation.

The CaCYP51 wild type protein–ligand complexes of the *R*- and *S*-enantiomers of compound **22** were generated, however, only the *S*-enantiomer showed interaction of the triazole with the haem (Figure S8). This finding was consistent with the crystallographic results described here, with clear electron density only obtained for the *S*-enantiomer **22** complexed with ScCYP51. Computational studies of other amide-linked long arm azole derivatives also describe preferential binding of the *S*-enantiomer with the resolved *S*-enantiomer of respective compounds showing potent antifungal activity (drug-sensitive *C. albicans*) compared with moderate activity observed for the *R*-enantiomer.^21,42^ Ligand *S*-**22** bound in the active site of CaCYP51 wild type, single and double mutants (Figure S9), with Fe–N distances of 2.42 Å (wild type), 2.58 Å (Y132F), 2.60 Å (Y132H), 2.73 Å (K143R), and 2.82 Å (Y132H + K143R). Additional binding interactions were observed in CaCYP51 wild-type and included hydrophobic interactions between the biphenyl moiety with Phe380 (π–π) and Met508 (vdW), and water-mediated hydrogen bonding interactions between the amide carbonyl and Leu121 and the tertiary hydroxy and Tyr132 (Figure 6a,b). Key binding interactions in the double mutant included hydrophobic interaction between the biphenyl moiety with His377 (vdW), and water-mediated hydrogen bonding interactions between the amide carbonyl and Leu121. Of note in the double mutant was the loss of the water-mediated interaction with the tertiary hydroxy owing to mutation of Tyr132 to His132 (Figures 6a,b and S9 and S10). The reduced binding interactions with *S*-**22** in the Y132H + K143R strain can be observed from the protein–ligand interactions histogram over the course of the 150 ns MD simulation (Figures 6d, S9 and S10). Reduced binding interactions may be sufficient to explain the reduced enzyme inhibitory activity of compound *S*-**22** against the CaCYP51 double mutant strain (Table 2), as the ability of the longer azoles such as PCZ and oteseconazole to form additional binding interactions in the access channel has been linked to improved antifungal activity and reduced resistance.^18^

**Figure 6:**
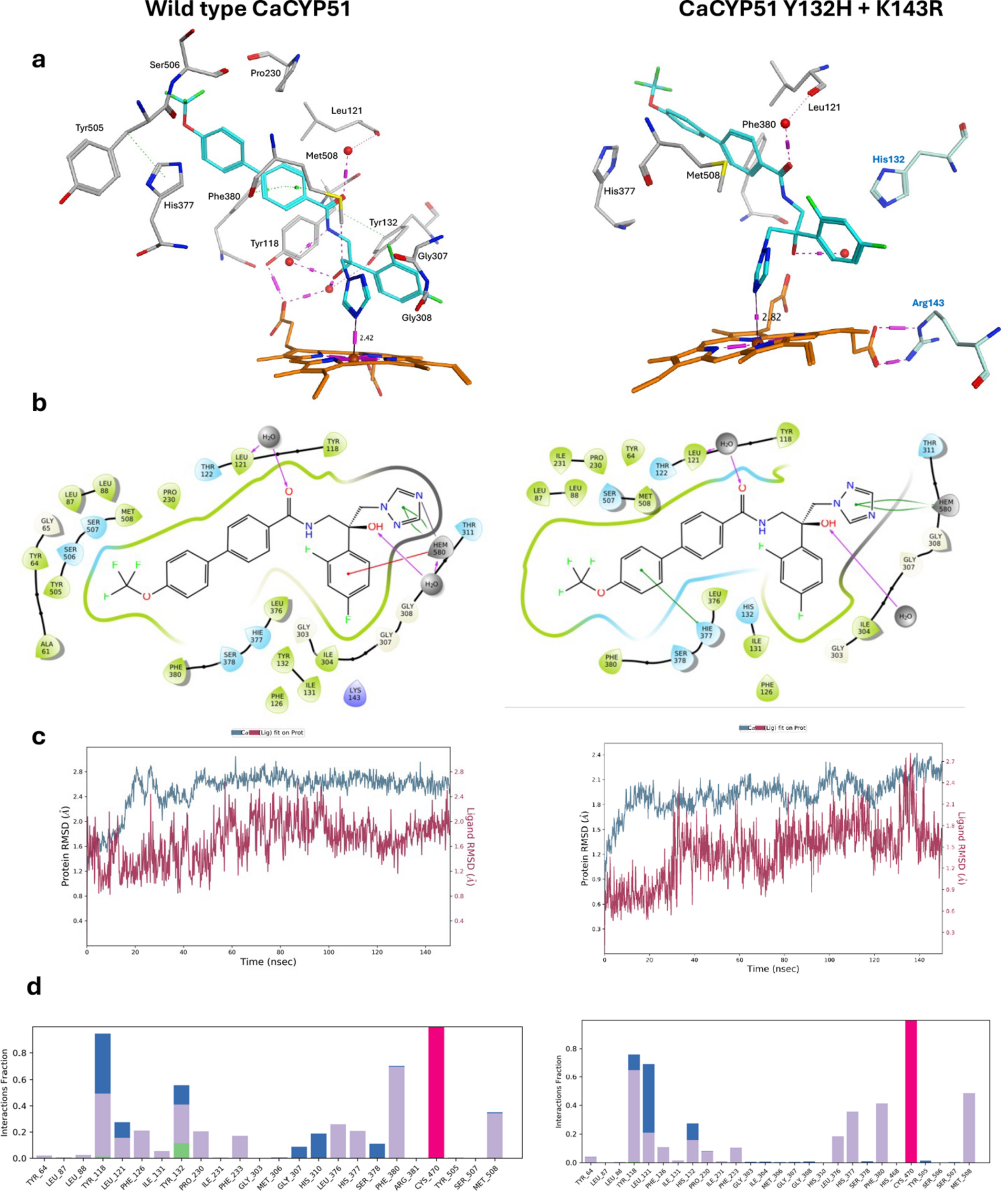
(a) Three-dimensional image of compound *S*-**22** in CaCYP51 wild-type and Y132H + K143R double mutant [Haem in orange, H_2_O shown as red spheres] (b) 2D ligand interactions (c) protein–ligand RMSD over 150 ns MD simulation time and (d) protein–ligand interactions over the course of the 150 ns MD simulation [Hydrophobic (purple), hydrogen bonds (green), ionic (pink), and water bridges (blue)].

The water-mediated hydrogen bond network between a haem propionate, Tyr132, and the tertiary hydroxyl of *S*-**22** of CaCYP51 WT, comparable to that observed in the ScCYP51-*S*-**22** crystal structure (Figure 4), was retained in the CaCYP51 Y132F and Y132H single mutants, although the interaction observed with Tyr132 was lost (Figures S9 and S10). However, in the single CaCYP51 K143R mutant, this water-mediated hydrogen bond network was lost as observed with the CaCYP51 Y132H + K143R double mutant (Figures 6 and S9 and S10), resulting from distortion of the haem.

### Evaluation of Broad-Spectrum Antifungal Activity

Minimum inhibitory concentrations (MIC_80_) values were obtained against *S. cerevisiae* recombinant models expressing wild-type C. glabrata, C. parapsilosis, C. auris, Rhizopus arhizus, *Cryptococcus neoformans*, and Aspergillus fumigatus CYPs (Table 4). MIC values were also obtained for *S. cerevisiae* strains expressing C. parapsilosis CYP51 Y132F, C. auris CYP51 Y132F and K143R-mediated azole resistance, and isoform-based innate resistance in R. arrhizus (CYP51 F5) and A. fumigatus (CYP51A). In addition, MIC values were obtained for clinically relevant MFS (MDR1a) and ABC (CDR1B) transporters from C. glabrata and C. auris expressed in *S. cerevisiae.*

**Table 4:** Susceptibility of S. cerevisiae Strains Expressing C. parapsilosis, C. glabrata, C.auris, C. neoformans, R. arrhizus, and A. fumigatus CYPs^a^ ^,^ ^b^

compound	14	22	23	24	26	PCZ
strain	MIC_80_(nM)					
*C. parapsilosis* models						
Y2721 OE CpCYP51	24	99 ± 0	55 ± 3	94		122 ± 37
Y2716 OE CpCYP51 Y132F	1000	210 ± 43	1906 ± 20	1750		135 ± 58
*C. glabrata* models						
Y2374 OE CgCYP51	47 ± 6	101	76 ± 12	168	205	332
Y433 OE CgCDR1	9200 ± 707	5850	13,100 ± 3800	11,800 ± 1400	>40,000	23,800
*C. auris* models						
Y2767 OE CauCYP51		110 ± 10				196 ± 22
Y2768 OE CauCYP51 Y132F		241 ± 38				168 ± 6
Y2769 OE CauCYP51 K143R		225 ± 33				117 ± 5
Y2765 OE CauMDR1		59 ± 14				96 ± 3
Y2766 OE CauCDR1		20,867 ± 7159				971 ± 49
*Cryptococcus neoformans* model						
2711 CnCYP51 OE CnCPR	5	18 ± 0	20 ± 0	2		35 ± 15
*Rhizopus arrhizus* models						
Y2649 OE CYP51 F1 RaCPR	83	470 ± 240	32 ± 19	16		100 ± 0
Y2651 OE CYP51 F5 RaCPR	3500	1356 ± 907	2567 ± 938	1400		54 ± 11
*Aspergillus fumigatus* models						
Y2746 OE AfCYP51A AfCPR AfERG6	>15,000	12,411 ± 834	3515 ± 209	2700		33 ± 14
Y2747 OE AfCYP51B AfCPR AfERG6	300	72 ± 29	150 ± 26	50		70 ± 24

a The endogenous ScCYP51 is deleted in all strains expressing recombinant CYP51s but not in strains expressing recombinant drug efflux pumps. OE indicates overexpression of following proteins.

b Values are presented as mean ± SD based on at least two separate experiments, with each experiment providing the mean of a pair of measurements (at least 4 measurements in total). When no SD is given the numbers represent screens which present the mean of a pair of measurements.

All exemplar compounds displayed improved inhibitory activity (MIC) against *S. cerevisiae* recombinant models expressing wild-type C. glabrata, C. parapsilosis, C. auris CYP51s compared with PCZ (Table 4), with a >19-fold increase in resistance owing to the CpCYP51 Y132F mutation for **14**, **23** and **24**, however resistance increased by only 2-fold for **22** (Table 4). Similarly, the CauCYP51 Y132F and K143R mutant enzymes conferred only a 2- to 2.5-fold increase in resistance to **22** compared with wild-type CauCYP51. Where tested, compounds **14**, **22**, **23**, **24,** and **26** showed activity as good as, or superior to PCZ against recombinant yeast strains expressing recombinant CgCYP51, CpCYP51, CnCYP51, and CauCYP51 (Table 4).

The test compounds were effluxed by strains expressing CgCDR1B or CauCDR1, in common with most azole antifungals. Strain Y2649 expressing the R. arrhizus CYP51 F1 isoform gave good susceptibilities to most of the test compounds. In contrast, strain Y2651 expressing the RaCYP51 F5 isoform responsible for innate azole resistance to short-tailed azoles, but not the long-tailed azole PCZ, gave dramatically reduced susceptibilities (>1000 nM), probably owing to the presence of the Y129F and V289A substitutions. Strain Y2747 expressing the A. fumigatus CYP51B isoform exhibited good susceptibility to each test compound, with **14** conferring the weakest susceptibility with an MIC_80_ of 300 nM. In contrast, strain Y2746 expressing the AfCYP51A isoform was poorly susceptible to each test compound (MIC_80_ > 1000 nM). The AfCYP51A isoform, which contains the T289A substitution responsible for innate resistance to FLC, oteseconazole, and difenoconazole, showed the expected susceptibility to PCZ.^43^

## Conclusions

Antifungal activity obtained, considering activity against both wild-type and resistant strains, allows for preliminary structure–activity studies. For derivatives containing the tertiary hydroxy group the biphenyl derivatives (**21**–**24**) were optimal followed by the simple naphthyl (**14**) and quinolone (**15**) derivatives, the diamide **26** was also promising, however the simple phenyl and thiazole derivatives (**11**–**13**), although promising against the wild-type strain showed loss of inhibitory activity against resistant strains (Figures 1, S1 and S2). Derivatives lacking the tertiary hydroxy group (**37**–**40** and **45**–**48**) showed significant loss of inhibitory activity against resistant strains using the agarose diffusion assay, with the exception of the diamide **44**, which retained modest inhibitory activity comparable with PCZ (Figures S3 and S4). MIC determination of compounds **14**, **22**, **23**, **24**, and **26** identified compound **22** as the most promising lead with respect to retention of inhibitory activity against model and clinical resistant strains (Tables 1 and 4). The improved antifungal activity (and broad-spectrum activity) of the biphenyl derivatives, in general, may be attributed to these compounds interacting with an additional target(s). Some evidence for this has been described for hybrid FLC–COX inhibitors, specifically a FLC-flurbiprofen hybrid, which contains a biphenyl moiety from the flurbiprofen segment, with antifungal activity mainly through inhibition of CYP51 and a second mechanism of action attributed to the COX-inhibiting segment.^21^

The physicochemical properties of compound **22** compared with FLC, VT-1161 (oteseconazole), and PCZ are shown in Table 5. From the properties: *c*log*P* (Crippen’s fragmentation^44^), molecular weight (*M*_w_), number of H-bond acceptors (*n*_ON_), H-bond donors (*n*_OHNH_), rotatable bonds (*n*_rot_), along with the topological polar surface area (TPSA) and molecular volume (MV) (Molinspiration^45^), compound **22** most closely resembles oteseconazole with both just outside of Lipinski’s Ro5 for *M*_w_ and *c*Log*P*, however, **22** shows improved properties related to drug-likeness compared with PCZ.

**Table 5:** Physicochemical Properties of **22** Compared with Those of Clinical Azole Antifungals^a^

compound	*M*_w_	*c*Log*P*	*n*_ON_/*n*_OHNH_	*n*_rot_	TPSA (Å^–2^)	MV (Å^3^)	*n*_Viol_
**22**	*518.4*	*5.39*	7/2	9	89.3	415.0	2
FLC	306.3	0.87	7/1	5	81.7	249.0	0
VT-1161	*527.4*	*5.20*	7/1	9	86.0	401.7	2
PCZ	*700.8*	*5.74*	12/1	12	115.7	623.4	3

a *n*_ON_, H-bond acceptor; *n*_OHNH_, H-bond donor; *n*_rot_, number of rotatable bonds; TPSA, topological polar surface area; MV, molecular volume; *n*_Viol_, number of Lipinsky violations (violations are italicized).

Agarose diffusion screens found that compound **22** had clear advantages over the other compounds tested. Compound **22** showed high potency against the *S. cerevisiae* host strain, the expected visible reduction in potency owing to overexpression of recombinant wild-type ScCYP51, an unaffected inhibition zone size with ScCYP51 Y140F/H mutants, and no effect on its potency owing to the expression of the CaMDR1a drug efflux pump. As found for all azole compounds tested, expression of the CaCDR1B drug efflux pump conferred resistance to **22**. The only other compound with a profile similar to that of **22** was **26**. Compound **26** contains a longer linker between the two phenyl groups in its long arm amide extension and therefore is likely to have different or additional interactions with the entry substrate channel. MIC determinations confirmed key observations made using agarose diffusion experiments. They showed compound **22** potently inhibited recombinant Saccharomyces, Candida, and Cryptococcus CYP51 enzymes expressed in *S. cerevisiae*, but not the A. fumigatus CYP51A isoform that confers resistance to FLC and the R. arrhizus CYP51 F5 isoform known to confer resistance to azole drugs except PCZ. A crystal structure of ScCYP51 revealed that it bound only the *S*-enantiomer of **22.** This binding mode involved coordination of the haem iron via the triazole nitrogen and through a water-mediated hydrogen bond network involving the tertiary hydroxyl and amide NH of **22**, a haem propionate, and Tyr140. The binding was strengthened by van der Waals interactions observed at the neck of the substrate entry channel. The water-mediated hydrogen bond network is particularly important in defining CYP51 affinity for triazole drugs such as FLC and VCZ, the tetrazole VT-1161 (oteseconazole), but not PCZ, which lacks a tertiary hydroxyl group. We have demonstrated that the mutation Y140F/H in S. cerevisiae,^18,25,46^ Y132F in C. auris^47^ and C. parapsilosis^48^ confer significant resistance to FLC, VCZ, oteseconazole but not PCZ. It is of considerable interest that the Y140F mutation in *S. cerevisiae* does not confer resistance to **22**, owing in part to the ability of the amide NH to form a compensatory hydrogen bond with the bridging water to the haem propionate. A hydrogen bond between the oxygen of the terminal trifluoromethoxy group of *S-* **22** and the His381 side chain of *S. cerevisiae* CYP51 must also contribute significantly to its binding and stabilization in the presence of the *S. cerevisiae* CYP51 Y140F/H, C. parapsilosis, and C. auris CYP51 Y132F mutations and the C. auris K143R mutation. The A. fumigatus CYP51A isoform containing a T289A substitution in helix I and the R. arrhizus CYP51 F5 isoform containing Y129F BC loop V291A helix I substitutions confer strong innate resistance to **22**, in contrast to the *S. cerevisiae* Y140F, C. auris Y132F, and C. parapsilosis CYP51 Y132F mutations in the BC-loop. This suggests that innate helix I substitutions in these mold CYP51 isoforms appear sufficient to confer resistance to **22**.

## Experimental Section

### Chemistry General Information

All chemicals, reagents, and solvents were purchased from Sigma-Aldrich, Alfa Aesar, VWR, Acros, and Fluka. Solvents were dried prior to use over molecular sieves (4 Å). For column chromatography, a glass column was slurry-packed in the appropriate eluent with silica gel (Fluka Kieselgel 60), and column chromatography was performed with the aid of a bellow. Analytical thin layer chromatography (TLC) was carried out on precoated silica plates (ALUGRAM SIL G/UV254) with UV light (254 nm) visualization. Melting points were determined using an electrothermal instrument (Gallenkamp melting point apparatus) and were uncorrected. ^1^H, ^19^F, and ^13^C (APT) NMR spectra were recorded on a Bruker Advance DP500 spectrometer operating at 500, 470, and 125 MHz, respectively, and auto-calibrated to the deuterated solvent reference peak and DMSO-*d*_6_ as the NMR solvent. Chemical shifts are given in parts per million (ppm) relative to the internal standard tetramethylsilane (Me_4_Si); coupling constants (*J*) are given in Hertz (Hz). High-performance liquid chromatography (HPLC)/high resolution mass spectra (HRMS) were performed by the University of Bath, Bath, UK, using an Infinity II 1260 HPLC coupled to a 6545 QTOF Mass spectrometer with electrospray ionization (Agilent). HPLC conditions were as follows: on a Zorbax Eclipse Plus C18 Rapid Resolution 2.1 × 50 mm column, 1.8 μm particle size, using a 7.5 min gradient method, mobile phase A was 0.1% formic acid in HPLC water, and B was 0.1% formic acid in methanol. The flow rate was 0.5 mL/min. The column temperature was at 50 °C, injection volume was 10 μL. Starting gradient at 5% B, 0.5 min begin gradient to 100% B, at 2.5 min hold at 100% B for 1 min, at 3.5 to 3.6 min fast gradient to 5% B hold until 7.5 min. Elemental analysis was performed by MEDAC Ltd., Chobham (UK).

Synthetic procedures and analytical data for all intermediate compounds, NMR spectra, and HPLC or elemental analysis for final compounds and computational methods can be found in the Supporting Information. All compounds are >95% pure by HPLC analysis.

#### General Method for Preparation of Amides **11**, **12**, **14–16**, **21–24**, and **37–40**

To a solution of carboxylic acid (1.0–1.5 m equiv) in dry DMF (5 mL/mmol) was added CDI (1.5 m equiv), and the reaction was stirred at room temperature for 1 h. Then, a solution of amine (1.0–1.2 m equiv) in dry DMF (5 mL/0.5 mmol) was added, and the reaction was stirred at room temperature overnight. The reaction mixture was quenched with ice/cooled H_2_O (25 mL), then the residue was extracted with EtOAc (50 mL), washed with brine (25 mL × 2), and dried (MgSO_4_). The organic layer was evaporated under reduced pressure, and the crude product was purified by gradient column chromatography.

##### 3-Acetyl-N-(2-(2,4-difluorophenyl)-2-hydroxy-3-(1H-1,2,4-triazol-1-yl)­propyl)­benzamide (**11**)

The product was prepared from 3-acetylbenzoic acid (**5**) (0.19 g, 1.17 mmol) and 1-amino-2-(2,4-difluorophenyl)-3-(1*H*-1,2,4-triazol-1-yl)­propan-2-ol (**4**) (0.2 g, 0.78 mmol). Purified using gradient chromatography eluting with CH_2_Cl_2_-MeOH 97:3 v/v to afford the product as a white semi-solid: Yield: 0.17 g (54%); TLC: CH_2_Cl_2_-MeOH 95:5 v/v, *R*_f_ 0.35; HPLC: 100% at R.T. 4.26 min. ^1^H NMR (DMSO-*d*_6_) δ: 8.37 (t, *J* = 6.0 Hz, 1H, NH), 8.34 (s, 1H, triaz), 8.30 (s, 1H, Ar), 8.09 (d, *J* = 7.8 Hz, 1H, Ar), 8.00 (d, *J* = 8.3 Hz, 1H, Ar), 7.75 (s, 1H, triaz), 7.61 (t, *J* = 7.8 Hz, 1H, Ar), 7.41 (dd, *J* = 9.0, 15.9 Hz, 1H, Ar), 7.21–7.16 (m, 1H, Ar), 6.93 (ddd, *J* = 2.6, 8.5, 11.0 Hz, 1H, Ar), 6.24 (s, 1H, OH, ex), 4.73 (d, *J* = 14.4 Hz, 1H, C*Ha*Hb-triaz), 4.60 (d, *J* = 14.4 Hz, 1H, CHa*Hb*-triaz), 3.84 (d, *J* = 14.0 Hz, 1H, C*Ha*Hb-NH), 3.7 (d, *J* = 14.0 Hz, 1H, CHa*Hb*-NH), 2.61 (s, 3H, CH_3_). ^13^C NMR (DMSO-*d*_6_) δ: 197.99 (C, C=O), 167.32 (C, C=O), 162.28 (dd,^3^ *J*_CF_ = 12.6 Hz,^1^ *J*_CF_ = 246.0 Hz, C, C2–Ar),159.60 (dd,^3^ *J*_CF_ = 12.5 Hz,^1^ *J*_CF_ = 247.3 Hz, C, C4–Ar), 150.99 (CH, triaz), 145.44 (CH, triaz), 137.20 (C, Ar), 134.79 (C, Ar), 132.34 (CH, Ar), 131.46 (CH Ar), 130.44 (dd,^3^ *J*_CF_ = 6.1 Hz,^3^ *J*_CF_ = 9.6 Hz, CH, C6–Ar), 129.29 (CH, Ar), 127.31 (CH, Ar), 125.20 (dd,^4^ *J*_CF_ = 3.5 Hz,^2^ *J*_CF_ = 13.23 Hz, C, C1–Ar), 111.15 (dd,^4^ *J*_CF_ = 3.1 Hz,^2^ *J*_CF_ = 20.6 Hz, CH, C5–Ar), 104.41 (t,^2^ *J*_CF_ = 26.2 Hz, CH, C3–Ar), 75.57 (C–OH), 55.53 (CH_2_–triaz), 47.09 (CH_2_–NH_2_), 27.31 (CH_3_). ^19^F NMR (DMSO-*d*_6_) δ: −106. 77 (*para*–F-Ar), and −112.08 (*ortho*–F-Ar). HRMS (ESI) *m*/*z* Calculated: 401.1425 [M + H]^+^, Found: 401.1425 [M + H]^+^.

##### 2-Chloro-N-(2-(2,4-difluorophenyl)-2-hydroxy-3-(1H-1,2,4-triazol-1-yl)­propyl)­thiazole-4-carboxamide (**12**)

The product was prepared from 2-chlorothiazole-4-carboxylic acid (**6**) (0.19 g, 1.17 mmol) and 1-amino-2-(2,4-difluorophenyl)-3-(1*H*-1,2,4-triazol-1-yl)­propan-2-ol (**4**) (0.2 g, 0.78 mmol). Purified using gradient chromatography, eluting with CH_2_Cl_2_-MeOH 98:2 v/v to afford the product as a white solid: Yield: 0.20 g (65%); mp 182–184 °C; TLC: CH_2_Cl_2_-MeOH 95:5 v/v, *R*_f_ 0.42; HPLC: 97.1% at R.T. 4.42 min. ^1^H NMR (DMSO-*d*_6_) δ: 8.32 (s, 1H, thiazole), 8.30 (t, *J* = 6.2 Hz, 1H, NH), 8.24 (s, 1H, triaz), 7.73 (s, 1H, triaz), 7.38 (dd, *J* = 9.0, 15.9 Hz, 1H, Ar), 7.19–7.15 (m, 1H, Ar), 6.93 (ddd, *J* = 2.4, 8.3, 10.9 Hz, 1H, Ar), 6.24 (s, 1H, OH, ex), 4.66 (d, *J* = 14.4 Hz, 1H, C*Ha*Hb-triaz), 4.54 (d, *J* = 14.4 Hz, 1H, CHa*Hb*-triaz), 3.85 (dd, *J* = 6.9, 14.0 Hz, 1H, C*Ha*Hb-NH), 3.74 (dd, *J* = 5.8, 13.9 Hz, 1H, CHa*Hb*-NH). ^13^C NMR (DMSO-*d*_6_) δ: 162.30 (dd,^3^ *J*_CF_ = 12.7 Hz,^1^ *J*_CF_ = 246.1 Hz, C, C2–Ar), 160.34 (C, C=O), 159.68 (dd,^3^ *J*_CF_ = 12.5 Hz,^1^ *J*_CF_ = 247.3 Hz, C, C4–Ar), 151.42 (C, thiazole), 151.01 (CH, triaz), 147.72 (C, thiazole), 145.46 (CH, triaz), 130.50 (dd,^3^ *J*_CF_ = 6.1 Hz,^3^ *J*_CF_ = 9.6 Hz, CH, C6–Ar), 128.10 (CH, Ar), 125.12 (dd,^4^ *J*_CF_ = 3.5 Hz,^2^ *J*_CF_ = 13.4 Hz, C, C1–Ar), 111.19 (dd, ^4^ *J*_CF_ = 3.0 Hz,^2^ *J*_CF_ = 20.4 Hz, CH, C5–Ar), 104.43 (t,^2^ *J*_CF_ = 27.9 Hz, CH, C3–Ar), 75.02 (C–OH), 55.57 (CH_2_–triaz), 46.16 (CH_2_–NH_2_). ^19^F NMR (DMSO-*d*_*6*_) δ: −106. 88 (*para*–F-Ar), −111.95 (*ortho*–F-Ar). HRMS (ESI) *m*/*z* Calculated: 422.0265 [M + Na]^+^, Found: 422.0262 [M + Na]^+^.

##### N-(2-(2,4-Difluorophenyl)-2-hydroxy-3-(1H-1,2,4-triazol-1-yl)­propyl)-6-fluoro-2-naphthamide (**14**)

The product was prepared from 6-fluoro-2-naphthoic acid (**8**) (0.22 g, 1.17 mmol) and 1-amino-2-(2,4-difluorophenyl)-3-(1*H*-1,2,4-triazol-1-yl)­propan-2-ol (**4**) (0.2 g, 0.78 mmol). Purified using gradient chromatography, eluting with CH_2_Cl_2_-MeOH 97.5:2.5 v/v to afford the product as a white solid: Yield: 0.20 g (60%); mp 195–197 °C; TLC: CH_2_Cl_2_-MeOH 95:5 v/v, *R*_f_ 0.37; HPLC: 96% at R.T. 4.59 min. ^1^H NMR (DMSO-*d*_*6*_) δ: 8.69 (t, *J* = 6.0 Hz, 1H, NH), 8.42 (s, 1H, Ar), 8.35 (s, 1H, triaz), 8.10 (dd, *J* = 5.8, 9.1 Hz 1H, Ar), 7.96 (d, *J* = 9.0 Hz, 1H, Ar), 7.88 (d, *J* = 8.8 Hz, 1H, Ar), 7.77 (dd, *J* = 2.6, 19.2 Hz, 1H, Ar), 7.75 (s, 1H, triaz), 7.51 (ddd, *J* = 2.7, 8.9, 11.5 Hz, 1H, Ar), 7.43 (dd, *J* = 9.0, 15.9 Hz, 1H, Ar), 7.21- 7.17 (m, 1H, Ar), 6.94 (ddd, *J* = 2.8, 8.7, 11.3 Hz, 1H, Ar), 6.32 (s, 1H, OH, ex), 4.74 (d, *J* = 14.4 Hz, 1H, C*Ha*Hb-triaz), 4.62 (d, *J* = 14.4 Hz, 1H, CHa*Hb*-triaz), 3.86 (dd, *J* = 6.2, 14.1 Hz, 1H, C*Ha*Hb-NH), 3.82 (dd, *J* = 5.8, 14.1 Hz, 1H, CHa*Hb*-NH). ^13^C NMR (DMSO-*d*_6_) δ: 167.97 (C, C = O), 162.39 (d,^1^ *J*_CF_ = 246.2 Hz, C, C6’-Ar), 160.9 (dd,^3^ *J*_CF_ = 12.7 Hz,^1^ *J*_CF_ = 245.4 Hz, C, C2–Ar), 159.21 (dd,^3^ *J*_CF_ = 12.5 Hz,^1^ *J*_CF_ = 247.4 Hz, C, C4–Ar), 150.9 (CH, triaz), 145.44 (CH, triaz), 135.62 (d,^3^ *J*_CF_ = 9.9 Hz, C, C10’-Ar), 132.38 (dd,^3^ *J*_CF_ = 9.3 Hz, CH, C8’-Ar), 131.22 (d,^4^ *J*_CF_ = 2.7 Hz, C, C9’-Ar), 130.53 (dd,^3^ *J*_CF_ = 6.1 Hz,^3^ *J*_CF_ = 9.5 Hz, CH, C6–Ar), 129.67 (C, C2’-Ar), 128.27 (CH, Ar), 127. 82 (d,^4^ *J*_CF_ = 5.3 Hz, CH, C4’-Ar), 125.67 (CH, Ar), 125.25 (dd,^4^ *J*_CF_ = 3.3 Hz,^2^ *J*_CF_ = 12.8 Hz, C, C4–Ar), 117.51 (d,^2^ *J*_CF_ = 25.4 Hz, CH, C7’-Ar), 111.26 (d, ^2^ *J*_CF_ = 20.7 Hz, CH, C5′-Ar) 111.18 (dd,^4^ *J*_CF_ = 3.1 Hz,^2^ *J*_CF_ = 20.7 Hz, CH, C5–Ar), 104.42 (t,^2^ *J*_CF_ = 27.1 Hz, CH, C3–Ar), 75.62 (C–OH), 55.57 (CH_2_–triaz), 47.20 (CH_2_–NH_2_). ^19^F NMR (DMSO-*d*_*6*_) δ: −106.76 (*para*–F-Ar), −112.09 (2F, *ortho*–F-Ar and naph-F). HRMS (ESI) *m*/*z* Calculated: 449.1201 [M + Na]^+^, Found: 449.1199 [M + Na]^+^.

##### N-(2-(2,4-Difluorophenyl)-2-hydroxy-3-(1H-1,2,4-triazol-1-yl)­propyl)­quinoline-3-carboxamide (**15**)

The product was prepared from quinoline-3-carboxylic acid (**9**) (0.20 g, 1.17 mmol) and 1-amino-2-(2,4-difluorophenyl)-3-(1*H*-1,2,4-triazol-1-yl)­propan-2-ol (**4**) (0.2 g, 0.78 mmol). Purified using gradient chromatography eluting with CH_2_Cl_2_-MeOH 97:3 v/v to afford the product as a white solid: Yield: 0.19 g (61%); mp 193–195; TLC: CH_2_Cl_2_-MeOH 95:5 v/v, *R*_f_ 0.30; HPLC: 100% at R.T. 4.34 min. ^1^H NMR (DMSO-*d*_*6*_) δ: 9.18 (d, *J* = 2.2 Hz, 1H, Ar), 8.87 (t, *J* = 6.1 Hz, 1H, NH), 8.75 (d, *J* = 2.2 Hz, 1H, Ar), 8.35 (s, 1H, triaz), 8.07 (d, *J* = 9.8 Hz, 2H, Ar), 7.88- 7.85 (m, 1H, Ar), 7.76 (s, 1H, triaz), 7.71–7.68 (m, 1H, Ar), 77.44 (dd, *J* = 9.0, 15.9 Hz, 1H, Ar), 7.22–7.17 (m, 1H, Ar), 6.94 (ddd, *J* = 2.5, 8.7, 10.9 Hz, 1H, Ar), 6.22 (s, 1H, OH, ex), 4.77 (d, *J* = 14.4 Hz, 1H, C*Ha*Hb-triaz), 4.65 (d, *J* = 14.4 Hz, 1H, CHa*Hb*-triaz), 3.86 (dd, *J* = 6.2, 13.8 Hz, 1H, C*Ha*Hb-NH), 3.83 (dd, *J* = 6.2, 13.8 Hz, 1H, CHa*Hb*-NH). ^13^C NMR (DMSO-*d*_6_) δ: 165.51 (C, C = O), 162.32 (dd,^3^ *J*_CF_ = 12.6 Hz,^1^ *J*_CF_ = 245.9 Hz, C, C2–Ar), 159.6 (dd,^3^ *J*_CF_ = 12.5 Hz,^1^ *J*_CF_ = 247.7 Hz, C, C4–Ar), 151.0 (CH, triaz), 149.32 (CH, Ar), 148.92 (C, Ar), 145.44 (CH, triaz), 136.16 (CH, Ar), 131.72 (CH, Ar), 130.53 (dd,^3^ *J*_CF_ = 6.1 Hz,^3^ *J*_CF_ = 9.5 Hz, CH, C6–Ar), 129.54 (CH, Ar), 129.21 (CH, Ar), 127. 90 (CH, Ar), 127.22 (C, Ar), 126.87 (C, Ar), 125.16 (dd,^4^ *J*_CF_ = 3.5 Hz,^2^ *J*_CF_ = 13.0 Hz, C, C1–Ar), 111.19 (dd,^4^ *J*_CF_ = 2.6 Hz,^2^ *J*_CF_ = 20.5 Hz, CH, C5–Ar), 104.44 (t,^2^ *J*_CF_ = 28.0 Hz, CH, C3–Ar), 75.55 (C–OH), 55.46 (CH_2_–triaz), 46.97 (CH_2_–NH_2_). ^19^F NMR (DMSO-*d*_*6*_) δ: −106.77 (*para*–F-Ar), −112.05 (*ortho*–F-Ar). HRMS (ESI) *m*/*z* Calculated: 410.1428 [M + H]^+^, Found: 410.1427 [M + H]^+^.

##### 7-Chloro-N-(2-(2,4-difluorophenyl)-2-hydroxy-3-(1H-1,2,4-triazol-1-yl)­propyl)-2-methylquinoline-3-carboxamide (**16**)

The product was prepared from 7-chloro-2-methylquinoline-3-carboxylic acid (**10**) (0.17 g, 0.78 mmol) and 1-amino-2-(2,4-difluorophenyl)-3-(1*H*-1,2,4-triazol-1-yl)­propan-2-ol (**4**) (0.2 g, 0.78 mmol). Purified using gradient chromatography eluting with CH_2_Cl_2_-MeOH 97:3 v/v to afford the product as a white solid: Yield: 0.22 g (63%); mp 145–146 °C; TLC: CH_2_Cl_2_-MeOH 95:5 v/v, *R*_f_ 0.45; HPLC: 100% at R.T. 4.51 min. ^1^H NMR (DMSO-*d*_6_) δ: 8.62 (t, *J* = 6.0 Hz, 1H, NH), 8.36 (s, 1H, triaz), 8.20 (s, 1H, Ar), 7.99 (d, *J* = 11.2 Hz, 1H, Ar), 7.98 (s, 1H, Ar), 7.79 (s, 1H, triaz), 7.62 (dd, *J* = 2.2, 9.0 Hz, 1H, Ar), 77.46 (q, *J* = 9.0 Hz, 1H, Ar), 7.23–7.18 (m, 1H, Ar), 7.00 (ddd, *J* = 2.5, 8.5, 11.0 Hz, 1H, Ar), 6.17 (s, 1H, OH, ex), 4.74 (d, *J* = 14.4 Hz, 1H, C*Ha*Hb-triaz), 4.66 (d, *J* = 14.4 Hz, 1H, CHa*Hb*-triaz), 4.00 (dd, *J* = 7.0, 13.9 Hz, 1H, C*Ha*Hb-NH), 3.67 (dd, *J* = 5.3, 13.9 Hz, 1H, CHa*Hb*-NH). ^13^C NMR (DMSO-*d*_6_) δ: 168.74 (C, C=O), 162.43 (dd,^3^ *J*_CF_ = 12.9 Hz,^1^ *J*_CF_ = 245.8 Hz, C, C2–Ar), 159.71 (dd,^3^ *J*_CF_ = 12.0 Hz,^1^ *J*_CF_ = 247.8 Hz, C, C4–Ar), 151.07 (CH, triaz), 147.72 (C, Ar), 145.50 (CH, triaz), 135.39 (C, Ar), 135.09 (CH, Ar), 130.99 (C, Ar), 130.78 (dd,^3^ *J*_CF_ = 6.0 Hz,^3^ *J*_CF_ = 9.7 Hz, CH, C6–Ar), 130.48 (CH, Ar), 127. 53 (CH, Ar), 127.24 (CH, Ar), 125.02 (dd,^4^ *J*_CF_ = 3.6 Hz,^2^ *J*_CF_ = 13.0 Hz, C, C1–Ar), 111.11 (dd,^4^ *J*_CF_ = 3.0 Hz,^2^ *J*_CF_ = 20.4 Hz, CH, C5–Ar), 104.44 (t,^2^ *J*_CF_ = 27.8 Hz, CH, C3–Ar), 75.31 (C–OH), 55.50 (CH_2_–triaz), 46.51 (CH_2_–NH_2_), 23.50 (CH_3_). ^19^F NMR (DMSO-*d*_6_) δ: −106.69 (*para*–F-Ar), −112.07 (*ortho*–F-Ar). HRMS (ESI) *m*/*z* Calculated: 480.1014 [M + Na]^+^, Found: 480.1013 [M + Na]^+^.

##### N-(2-(2,4-Difluorophenyl)-2-hydroxy-3-(1H-1,2,4-triazol-1-yl)­propyl)-4’-(trifluoromethyl)-[1,1’-biphenyl]-4-carboxamide (**21**)

The product was prepared from 4’-(trifluoromethyl)-[1,1’-biphenyl]-4-carboxylic acid (**17**) (0.2 g, 0.78 mmol) and 1-amino-2-(2,4-difluorophenyl)-3-(1*H*-1,2,4-triazol-1-yl)­propan-2-ol (**4**) (0.15 g, 0.58 mmol). Purified using gradient chromatography eluting with CH_2_Cl_2_-MeOH 97.5:2.5 v/v to afford the product as a white solid: Yield: 0.30 g (76%); mp 211–213 °C; TLC: CH_2_Cl_2_-MeOH 95:5 v/v, *R*_f_ 0.37; HPLC: 100% at R.T. 4.76 min. ^1^H NMR (DMSO-*d*_6_) δ: 8.61 (t, *J* = 6.0 Hz, 1H, NH), 8.35 (s, 1H, triaz), 7.94 (d, *J* = 8.1 Hz, 2H, Ar), 7.89 (d, *J* = 8.9 Hz, 2H, Ar), 7.83 (dd, *J* = 5.7, 7.8 Hz, 4H, Ar), 7.75 (s, 1H, triaz), 7.42 (dd, *J* = 9.0, 15.9 Hz, 1H, Ar), 7.21–7.16 (m, 1H, Ar), 6.93 (ddd, *J* = 2.7, 8.6, 11.1 Hz, 1H, Ar), 6.29 (s, 1H, OH, ex), 4.73 (d, *J* = 14.4 Hz, 1H, C*Ha*Hb-triaz), 4.60 (d, *J* = 14.4 Hz, 1H, CHa*Hb*-triaz), 3.85 (dd, *J* = 6.5, 14.0 Hz, 1H, C*Ha*Hb-NH), 3.79 (dd, *J* = 5.7, 14.0 Hz, 1H, CHa*Hb*-NH). ^13^C NMR (DMSO-*d*_*6*_) δ: 167.61 (C, C=O), 162.28 (dd,^3^ *J*_CF_ = 12.1 Hz,^1^ *J*_CF_ = 245.5 Hz, C, C2–Ar), 159.60 (dd,^3^ *J*_CF_ = 12.2 Hz,^1^ *J*_CF_ = 247.2 Hz, C, C4–Ar), 150.9 (CH, triaz), 145.57 (CH, triaz), 143.57 (C, Ar), 141.77 (C, Ar), 134.05 (C, Ar), 130.51 (dd,^3^ *J*_CF_ = 6.2 Hz,^3^ *J*_CF_ = 9.6 Hz, CH, C6–Ar), 128.82 (d,^2^ *J*_CF3_ = 31.9 Hz, C, C4”-Ar), 128.69 (2 × CH, Ar), 128.57 (2 × CH, Ar), 127. 42 (2 × CH, Ar), 126.30 (q,^3^ *J*_CF3_ = 3.5 Hz, 2 × CH, C3”and C5”-Ar), 125.22 (dd,^4^ *J*_CF_ = 3.4 Hz,^2^ *J*_CF_ = 12.9 Hz, C, C1–Ar), 124.64 (q,^1^ *J*_CF3_ = 256.18 Hz, C, CF_3_), 111.16 (dd, ^4^ *J*_CF_ = 2.4 Hz,^2^ *J*_CF_ = 20.6 Hz, CH, C5–Ar), 104.41 (t,^2^ *J*_CF_ = 26.3 Hz, CH, C3–Ar), 75.64 (C–OH), 55.57 (CH_2_–triaz), 47.08 (CH_2_–NH_2_). ^19^F NMR (DMSO-*d*_6_) δ: −60.96 (CF_3_), −106. 79 (*para*–F-Ar), and −112.15 (*ortho*–F-Ar). HRMS (ESI) *m*/*z* Calculated: 503.1506 [M + H]^+^, Found: 503.1503 [M + H]^+^.

##### N-(2-(2,4-Difluorophenyl)-2-hydroxy-3-(1H-1,2,4-triazol-1-yl)­propyl)-4’-(trifluoromethoxy)-[1,1’-biphenyl]-4-carboxamide (**22**)

The product was prepared from 4’-(trifluoromethoxy)-[1,1’-biphenyl]-4-carboxylic acid (**18**) (0.22 g, 0.78 mmol) and 1-amino-2-(2,4-difluorophenyl)-3-(1*H*-1,2,4-triazol-1-yl)­propan-2-ol (**4**) (0.2 g, 0.78 mmol). Purified using gradient chromatography eluting with CH_2_Cl_2_-MeOH 97.5:2.5 v/v to afford the product as a white solid: Yield: 0.26 g (65%); mp 208–210 °C; TLC: CH_2_Cl_2_-MeOH 95:5 v/v, *R*_f_ 0.35; HPLC: 100% at R.T. 4.78 min. ^1^H NMR (DMSO-*d*_*6*_) δ: 8.59 (t, *J* = 6.0 Hz, 1H, NH), 8.35 (s, 1H, triaz), 7.85 (q, *J* = 8.7 Hz, 4H, Ar), 7.77 (d, *J* = 8.7 Hz, 2H, Ar), 7.75 (s, 1H, triaz), 7.47 (d, *J* = 7.9 Hz, 2H, Ar), 7.42 (dd, *J* = 9.0, 15.9 Hz, 1H, Ar), 7.21- 7.16 (m, 1H, Ar), 6.93 (ddd, *J* = 2.4, 8.3, 10.9 Hz, 1H, Ar), 6.30 (s, 1H, OH, ex), 4.72 (d, *J* = 14.4 Hz, 1H, C*Ha*Hb-triaz), 4.60 (d, *J* = 14.4 Hz, 1H, CHa*Hb*-triaz), 3.84 (dd, *J* = 6.5, 14.0 Hz, 1H, C*Ha*Hb-NH), 3.79 (dd, *J* = 5.7, 14.0 Hz, 1H, CHa*Hb*-NH). ^13^C NMR (DMSO-*d*_6_) δ: 167.70 (C, C=O), 162.29 (dd,^3^ *J*_CF_ = 12.4 Hz,^1^ *J*_CF_ = 245.3 Hz, C, C2–Ar), 159.60 (dd,^3^ *J*_CF_ = 12.7 Hz,^1^ *J*_CF_ = 247.2 Hz, C, C4–Ar), 150.9 (CH, triaz), 148.72 (C, Ar), 145.46 (CH, triaz), 141.94 (C, Ar), 138.88 (C, Ar), 133.81 (C, Ar), 130.51 (dd,^3^ *J*_CF_ = 6.0 Hz,^3^ *J*_CF_ = 9.5 Hz, CH, C6–Ar), 129.30 (2 × CH, Ar), 128.51 (2 × CH, Ar), 127. Fifteen (2 × CH, Ar), 125.23 (dd,^4^ *J*_CF_ = 3.5 Hz,^2^ *J*_CF_ = 13.3 Hz, C, C1–Ar), 123.20 (q,^1^ *J*_CF3_ = 256.23 Hz, C, CF_3_), 121.97 (2 × CH, Ar), 111.16 (dd, ^4^ *J*_CF_ = 2.8 Hz,^2^ *J*_CF_ = 20.7 Hz, CH, C5–Ar), 104.40 (t,^2^ *J*_CF_ = 28.1 Hz, CH, C3–Ar), 75.61 (C–OH), 55.57 (CH_2_–triaz), 47.09 (CH_2_–NH_2_). ^19^F NMR (DMSO-*d*_*6*_) δ: −56.69 (CF_3_), −106. 81 (*para*–F-Ar), and −112.12 (*ortho*–F-Ar). HRMS (ESI) *m*/*z* Calculated: 519.1455 [M + H]^+^, Found: 519.1453 [M + H]^+^.

##### N-(2-(2,4-Difluorophenyl)-2-hydroxy-3-(1H-1,2,4-triazol-1-yl)­propyl)-4’-(trifluoromethyl)-[1,1’-biphenyl]-3-carboxamide (**23**)

The product was prepared from 4’-(trifluoromethyl)-[1,1’-biphenyl]-3-carboxylic acid (**19**) (0.24 g, 0.90 mmol) and 1-amino-2-(2,4-difluorophenyl)-3-(1*H*-1,2,4-triazol-1-yl)­propan-2-ol (**4**) (0.23 g, 0.90 mmol). Purified using gradient chromatography eluting with CH_2_Cl_2_-MeOH 97:3 v/v to afford the product as a white solid: Yield: 0.34 g (75%); mp 92–94 °C; TLC: CH_2_Cl_2_-MeOH 95:5 v/v, *R*_f_ 0.42; HPLC: 100% at R.T. 4.77 min. ^1^H NMR (DMSO-*d*_6_) δ: 8.72 (t, *J* = 6.0 Hz, 1H, NH), 8.35 (s, 1H, triaz), 8.09 (s, 1H, Ar), 7.94 (d, *J* = 8.1 Hz, 2H, Ar), 7.90 (d, *J* = 7.7 Hz, 1H, Ar), 7.86 (d, *J* = 8.2 Hz, 2H, Ar), 7.81 (d, *J* = 8.2 Hz, 1H, Ar), 7.75 (s, 1H, triaz), 7.59 (t, *J* = 7.7 Hz, 1H, Ar), 7.43 (dd, *J* = 9.0, 15.9 Hz, 1H, Ar), 7.21–7.17 (m, 1H, Ar), 6.94 (ddd, *J* = 2.5, 8.4, 10.9 Hz, 1H, Ar), 6.27 (s, 1H, OH, ex), 4.74 (d, *J* = 14.4 Hz, 1H, C*Ha*Hb-triaz), 4.61 (d, *J* = 14.4 Hz, 1H, CHa*Hb*-triaz), 3.85 (d, *J* = 6.0, 13.9 Hz, 1H, C*Ha*Hb-NH), 3.79 (d, *J* = 6.0, 13.9 Hz, 1H, CHa*Hb*-NH). ^13^C NMR (DMSO-*d*_*6*_) δ: 167.87 (C, C=O), 162.34 (dd,^3^ *J*_CF_ = 12.3 Hz,^1^ *J*_CF_ = 245.6 Hz, C, C2–Ar), 159.65 (dd,^3^ *J*_CF_ = 12.2 Hz, ^1^ *J*_CF_ = 247.6 Hz, C, C4–Ar), 151.01 (CH, triaz), 145.72 (CH, triaz), 143.92 (C, Ar), 139.01 (C, Ar), 135.25 (C, Ar), 130.50 (dd,^3^ *J*_CF_ = 6.0 Hz,^3^ *J*_CF_ = 9.7 Hz, CH, C6–Ar), 130.40 (CH, Ar), 129.69 (CH, Ar), 128.63 (d,^2^ *J*_CF3_ = 31.8 Hz, C, C4”-Ar), 128.11 (3 × CH, Ar), 127. 95 (CH, Ar), 126.31 (q,^3^ *J*_CF3_ = 3.3 Hz, 2 × CH, C3”and C5”-Ar), 125.24 (dd,^4^ *J*_CF_ = 3.3 Hz,^2^ *J*_CF_ = 13.2 Hz, C, C1–Ar), 124.66 (q,^1^ *J*_CF3_ = 256.4 Hz, C, CF_3_), 111.18 (dd,^4^ *J*_CF_ = 2.8 Hz,^2^ *J*_CF_ = 20.7 Hz, CH, C5–Ar), 104.42 (t,^2^ *J*_CF_ = 28.0 Hz, CH, C3–Ar), 75.64 (C–OH), 55.52 (CH_2_–triaz), 47.12 (CH_2_–NH_2_). ^19^F NMR (DMSO-*d*_*6*_) δ: −60.90 (CF_3_), −106. 80 (*para*–F-Ar), and −112.08 (*ortho*–F-Ar). HRMS (ESI) *m*/*z* Calculated: 503.1506 [M + H]^+^, Found: 503.1505 [M + H]^+^.

##### N-(2-(2,4-Difluorophenyl)-2-hydroxy-3-(1H-1,2,4-triazol-1-yl)­propyl)-4’-(trifluoromethoxy)-[1,1’-biphenyl]-3-carboxamide (**24**)

The product was prepared from 4’-(trifluoromethoxy)-[1,1’-biphenyl]-3-carboxylic acid (**20**) (0.22 g, 0.78 mmol) and 1-amino-2-(2,4-difluorophenyl)-3-(1*H*-1,2,4-triazol-1-yl)­propan-2-ol (**4**) (0.23 g, 0.90 mmol). Purified using gradient chromatography, eluting with CH_2_Cl_2_-MeOH 98:2 v/v to afford the product as a pale yellow oil: Yield: 0.27 g (72%); TLC: CH_2_Cl_2_-MeOH 95:5 v/v, *R*_f_ 0.40; HPLC: 97% at R.T. 4.79 min. ^1^H NMR (DMSO-*d*_6_) δ: 8.69 (t, *J* = 6.1 Hz, 1H, NH), 8.35 (s, 1H, triaz), 8.03 (s,1H, Ar), 7.83 (d, *J* = 8.9 Hz, 3H, Ar), 7.77 (d, *J* = 8.4 Hz, 1H, Ar), 7.75 (s, 1H, triaz), 7.56 (t, *J* = 7.7 Hz, 1H, Ar), 7.49 (d, *J* = 8.0 Hz, 2H, Ar), 7.42 (dd, *J* = 9.0, 15.9 Hz, 1H, Ar), 7.21–7.17 (m, 1H, Ar), 6.94 (ddd, *J* = 2.7, 8.6, 11.2 Hz, 1H, Ar), 6.28 (s, 1H, OH, ex), 4.73 (d, *J* = 14.4 Hz, 1H, C*Ha*Hb-triaz), 4.60 (d, *J* = 14.4 Hz, 1H, CHa*Hb*-triaz), 3.84 (d, *J* = 6.2, 14.3 Hz, 1H, C*Ha*Hb-NH), 3.78 (d, *J* = 5.7, 14.3 Hz, 1H, CHa*Hb*-NH). ^13^C NMR (DMSO-*d*_6_) δ: 167.95 (C, C=O), 162.29 (dd,^3^ *J*_CF_ = 12.7 Hz,^1^ *J*_CF_ = 245.9 Hz, C, C2–Ar), 159.60 (dd,^3^ *J*_CF_ = 12.0 Hz,^1^ *J*_CF_ = 247.0 Hz, C, C4–Ar), 150.9 (CH, triaz), 148.55 (C, Ar), 145.44 (CH, triaz), 139.27 (C, Ar), 139.16 (C, Ar), 135.13 (C, Ar), 130.50 (dd,^3^ *J*_CF_ = 6.2 Hz,^3^ *J*_CF_ = 9.7 Hz, CH, C6–Ar), 130.18 (CH, Ar), 129.58 (CH, Ar), 129.24 (2 × CH, Ar), 127. 40 (CH, Ar), 126.17 (CH, Ar), 125.24 (dd,^4^ *J*_CF_ = 3.6 Hz,^2^ *J*_CF_ = 13.3 Hz, C, C1–Ar), 123.04 (q,^1^ *J*_CF3_ = 256.2 Hz, C, CF_3_), 121.99 (2 × CH, Ar), 111.18 (dd,^4^ *J*_CF_ = 2.7 Hz,^2^ *J*_CF_ = 20.6 Hz, CH, C5–Ar), 104.42 (t,^2^ *J*_CF_ = 28.1 Hz, CH, C3–Ar), 75.65 (C–OH), 55.52 (CH_2_–triaz), 47.20 (CH_2_–NH_2_). ^19^F NMR (DMSO-*d*_*6*_) δ: −56.74 (CF_3_), −106. 81 (*para*–F-Ar), and −112.09 (*ortho*–F-Ar). HRMS (ESI) *m*/*z* Calculated: 519.1455 [M + H]^+^, Found: 519.1453 [M + H]^+^.

##### N-([1,1’-Biphenyl]-4-ylmethyl)-2-(2,4-difluorophenyl)-3-(1H-1,2,4-triazol-1-yl)­propenamide (**37**)

The product was prepared from 2-(2,4-difluorophenyl)-3-(1*H*-1,2,4-triazol-1-yl)­propanoic acid (**32**) (0.15 g, 0.59 mmol) and [1,1’-biphenyl]-4-yl-methanamine (**33**) (0.13 g, 0.71 mmol). Purified using gradient chromatography eluting with CH_2_Cl_2_-MeOH 97.5:2.5 v/v to afford the product as a white solid: Yield: 0.16 g (66%); mp 194–195 °C; TLC: CH_2_Cl_2_-MeOH 95:5 v/v, *R*_f_ 0.37; HPLC: 100% at R.T. 4.69 min. ^1^H NMR (DMSO-*d*_6_) δ: 8.77 (t, *J* = 5.9 Hz, 1H, NH), 8.35 (s, 1H, triaz), 7.98 (s, 1H, triaz), 7.63 (d, *J* = 7.2 Hz, 2H, Ar), 7.58 (dd, *J* = 8.7, 15.3 Hz, 1H, Ar), 7.54 (d, *J* = 8.4 Hz, 2H, Ar), 7.45 (t, *J* = 7.3 Hz, 2H, Ar), 7.35 (ttt, *J* = 1.2, 1.7, 1.3 Hz, 1H, Ar), 7.23 (ddd, *J* = 2.6, 9.3, 10.4 Hz, 1H, Ar), 7.12 (d, *J* = 8.5 Hz, 2H, Ar), 7.11 (ddd, *J* = 2.5, 8.4, 10.9 Hz, 1H, Ar), 4.81 (dd, *J* = 7.8, 12.7 Hz, 1H, C*Ha*Hb-triaz), 4.52–4.44 (m, 2H, C*H*CHa*Hb*-triaz), 4.31 (dd, *J* = 6.2, 15.4 Hz, 1H, C*Ha*Hb-NH), 4.20 (dd, *J* = 5.6, 15.4 Hz, 1H, CHa*Hb*-NH). ^13^C NMR (DMSO-*d*_6_) δ: 169.60 (C, C=O), 162.29 (dd,^3^ *J*_CF_ = 12.2 Hz, ^1^ *J*_CF_ = 189.9 Hz, C, C2–Ar), 160.32 (dd,^3^ *J*_CF_ = 12.6 Hz, ^1^ *J*_CF_ = 191.6 Hz, C, C4–Ar), 152.03 (CH, triaz), 145.06 (CH, triaz), 140.34 (C, Ar), 139.14 (C, Ar), 138.61 (C, Ar), 130.96 (dd,^3^ *J*_CF_ = 5.2 Hz,^3^ *J*_CF_ = 9.9 Hz, CH, C6–Ar), 129.36 (CH × 2, Ar), 127.94 (CH × 2, Ar), 127.78 (CH, Ar), 127.01 (CH × 2, Ar), 126.95 (CH × 2, Ar), 120.66 (dd,^4^ *J*_CF_ = 3.9 Hz,^2^ *J*_CF_ = 15.0 Hz, CH, C1–Ar), 112.017 (dd,^4^ *J*_CF_ = 3.5 Hz,^2^ *J*_CF_ = 21.0 Hz, CH, C5–Ar), 104.38 (t,^2^ *J*_CF_ = 26.3 Hz, CH, C3–Ar), 50.53 (CH_2_-triaz), 44.06 (CH), 42.32 (CH_2_–NH). ^19^F NMR (DMSO-*d*_*6*_) δ: −111.07 (*para*–F-Ar), −112.92 (*ortho*–F-Ar). HRMS (ESI) *m*/*z* Calculated: 441.1502 [M + Na]^+^, Found: 441.1498 [M + Na]^+^.

##### N-((4’-Chloro-[1,1’-biphenyl]-4-yl)­methyl)-2-(2,4-difluorophenyl)-3-(1H-1,2,4-triazol-1-yl)­propenamide (**38**)

The product was prepared from 2-(2,4-difluorophenyl)-3-(1*H*-1,2,4-triazol-1-yl)­propanoic acid (**32**) (0.15 g, 0.59 mmol) and (4’-chloro-[1,1’-biphenyl]-4-yl)­methanamine (**34**) (0.15 g, 0.71 mmol). Purified using gradient chromatography eluting with CH_2_Cl_2_-MeOH 98:2 v/v to afford the product as a white solid: Yield: 0.14 g (53%); mp 198–200 °C; TLC: CH_2_Cl_2_-MeOH 95:5 v/v, *R*_f_ 0.40; HPLC: 100% at R.T. 4.80 min. ^1^H NMR (DMSO-*d*_6_) δ: 8.78 (t, *J* = 5.9 Hz, 1H, NH), 8.35 (s, 1H, triaz), 7.98 (s, 1H, triaz), 7.66 (d, *J* = 8.7 Hz, 2H, Ar), 7.57 (dd, *J* = 8.7, 15.3 Hz, 1H, Ar), 7.55 (d, *J* = 8.4 Hz, 2H, Ar), 7.50 (d, *J* = 8.7 Hz, 2H, Ar), 7.23 (ddd, *J* = 2.6, 9.4, 10.5 Hz, 1H, Ar), 7.11 (d, *J* = 8.4 Hz, 2H, Ar), 7.10 (ddd, *J* = 2.4, 8.4, 10.0 Hz, 1H, Ar), 4.81 (dd, *J* = 7.8, 12.8 Hz, 1H, C*Ha*Hb-triaz), 4.52–4.44 (m, 2H, C*H*CHa*Hb*-triaz), 4.31 (dd, *J* = 6.2, 15.4 Hz, 1H, C*Ha*Hb-NH), 4.20 (dd, *J* = 5.6, 15.4 Hz, 1H, CHa*Hb*-NH). ^13^C NMR (DMSO-*d*_6_) δ: 169.62 (C, C=O), 162.29 (dd,^3^ *J*_CF_ = 12.5 Hz, ^1^ *J*_CF_ = 190.7 Hz, C, C2–Ar), 160.32 (dd,^3^ *J*_CF_ = 12.1 Hz, ^1^ *J*_CF_ = 192.6 Hz, C, C4–Ar), 152.03 (CH, triaz), 145.06 (CH, triaz), 139.13 (C, Ar), 139.06 (C, Ar), 137.76 (C, Ar), 132.65 (C, Ar), 130.96 (dd,^3^ *J*_CF_ = 5.2 Hz, ^3^ *J*_CF_ = 9.9 Hz, CH, C6–Ar), 129.30 (CH × 2, Ar), 128.76 (CH × 2, Ar), 127.99 (CH × 2, Ar), 126.90 (CH × 2, Ar), 120.66 (dd,^4^ *J*_CF_ = 3.8 Hz, ^2^ *J*_CF_ = 15.1 Hz, CH, C1–Ar), 112.017 (dd,^4^ *J*_CF_ = 3.5 Hz,^2^ *J*_CF_ = 21.1 Hz, CH, C5–Ar), 104.38 (t,^2^ *J*_CF_ = 26.5 Hz, CH, C3–Ar), 50.53 (CH_2_-triaz), 44.06 (CH), 42.29 (CH_2_–NH). ^19^F NMR (DMSO-*d*_6_) δ: −111.05 (*para*–F-Ar), −112.95 (*ortho*–F-Ar). HRMS (ESI) *m*/*z* Calculated: 451.1137 [M – H]^−^, Found: 451.1142 [M – H]^−^.

##### 2-(2,4-Difluorophenyl)-3-(1H-1,2,4-triazol-1-yl)-N-((4’-(trifluoromethyl)-[1,1’-biphenyl]-4-yl)­methyl)­propenamide (**39**)

The product was prepared from 2-(2,4-difluorophenyl)-3-(1*H*-1,2,4-triazol-1-yl)­propanoic acid (**32**) (0.15 g, 0.59 mmol) and (4’-(trifluoromethyl)-[1,1’-biphenyl]-4-yl)­methanamine (**35**) (0.20 g, 0.71 mmol). Purified using gradient chromatography eluting with CH_2_Cl_2_-MeOH 98:2 v/v to afford the product as a white solid: Yield: 0.15 g (54%); mp 197–198 °C; TLC: CH_2_Cl_2_-MeOH 95:5 v/v, *R*_f_ 0.42; HPLC: 100% at R.T. 4.79 min. ^1^H NMR (DMSO-*d*_6_) δ: 8.80 (t, *J* = 5.9 Hz, 1H, NH), 8.35 (s, 1H, triaz), 7.98 (s, 1H, triaz), 7.86 (d, *J* = 8.2 Hz, 2H, Ar), 7.80 (d, *J* = 8.2 Hz, 2H, Ar), 7.63 (d, *J* = 8.4 Hz, 2H, Ar), 7.58 (dd, *J* = 8.7, 15.3 Hz, 1H, Ar), 7.23 (ddd, *J* = 2.6, 9.4, 10.4 Hz, 1H, Ar), 7.15 (d, *J* = 8.4 Hz, 2H, Ar), 7.11 (ddd, *J* = 2.4, 8.4, 10.7 Hz, 1H, Ar), 4.81 (dd, *J* = 7.9, 12.9 Hz, 1H, C*Ha*Hb-triaz), 4.52- 4.44 (m, 2H, C*H*CHa*Hb*-triaz), 4.33 (dd, *J* = 6.2, 15.5 Hz, 1H, C*Ha*Hb-NH), 4.22 (dd, *J* = 5.6, 15.5 Hz, 1H, CHa*Hb*-NH). ^13^C NMR (DMSO-*d*_*6*_) δ: 169.65 (C, C=O), 162.29 (dd,^3^ *J*_CF_ = 12.5 Hz,^1^ *J*_CF_ = 191.1 Hz, C, C2–Ar), 160.32 (dd,^3^ *J*_CF_ = 12.6 Hz,^1^ *J*_CF_ = 193.4 Hz, C, C4–Ar), 152.02 (CH, triaz), 145.06 (CH, triaz), 144.32 (C, Ar), 139.80 (C, Ar), 137.50 (C, Ar), 130.96 (dd,^3^ *J*_CF_ = 5.0 Hz,^3^ *J*_CF_ = 10.1 Hz, CH, C6–Ar), 128.16 (d,^2^ *J*_CF3_ = 31.9 Hz, C, C4”-Ar), 128.06 (CH × 2, Ar), 127.78 (CH × 2, Ar), 127.33 (CH × 2, Ar), 126.20 (q,^3^ *J*_CF3_ = 3.7 Hz, CH × 2, C3”and C5”-Ar), 123.29 (q,^1^ *J*_CF_ = 203.1 Hz, CF_3_), 120.62 (dd,^4^ *J*_CF_ = 3.8 Hz,^2^ *J*_CF_ = 15.3 Hz, CH, C1–Ar), 112.018 (dd,^4^ *J*_CF_ = 3.6 Hz, ^2^ *J*_CF_ = 21.5 Hz, CH, C5–Ar), 104.38 (t,^2^ *J*_CF_ = 26.5 Hz, CH, C3–Ar), 50.51 (CH_2_-triaz), 44.06 (CH), 42.28 (CH_2_–NH). ^19^F NMR (DMSO-*d*_6_) δ: −60.86 (CF_3_), −111.04 (*para*–F-Ar), and −112.95 (*ortho*–F-Ar).

HRMS (ESI) *m*/*z* Calculated: 487.1557 [M + H]^+^, Found: 487.1553 [M + H]^+^.

##### 2-(2,4-Difluorophenyl)-3-(1H-1,2,4-triazol-1-yl)-N-((4’-(trifluoromethoxy)-[1,1’-biphenyl]-4-yl)­methyl)­propenamide (**40**)

The product was prepared from 2-(2,4-difluorophenyl)-3-(1*H*-1,2,4-triazol-1-yl)­propanoic acid (**32**) (0.3 g, 1.18 mmol) and (4’-(trifluoromethoxy)-[1,1’-biphenyl]-4-yl)­methanamine (**36**) (0.37 g, 1.42 mmol). Purified using gradient chromatography eluting with CH_2_Cl_2_-MeOH 97:3 v/v followed by recrystallization (EtOH) to afford the product as a white solid: Yield: 0.24 g (40%); mp 160–162 °C; TLC: CH_2_Cl_2_-MeOH 95:5 v/v, *R*_f_ 0.55; HPLC: 100% at R.T. 4.79 min. ^1^H NMR (DMSO-*d*_6_) δ: 8.79 (t, *J* = 5.9 Hz, 1H, NH), 8.36 (s, 1H, triaz), 7.98 (s, 1H, triaz), 7.76 (d, *J* = 8.9 Hz, 2H, Ar), 7.60- 7.56 (m, 1H, Ar), 7.57 (d, *J* = 8.4 Hz, 2H, Ar), 7.44 (dd, *J* = 0.9, 8.8 Hz, 2H, Ar), 7.23 (ddd, *J* = 2.7, 9.4, 10.5 Hz, 1H, Ar), 7.13 (d, *J* = 8.5 Hz, 2H, Ar), 7.11 (ddd, *J* = 2.4, 8.5, 10.8 Hz, 1H), 4.81 (dd, *J* = 7.9, 12.9 Hz, 1H, C*Ha*Hb-triaz), 4.52- 4.44 (m, 2H, C*H*CHa*Hb*-triaz), 4.32 (dd, *J* = 6.2, 15.4 Hz, 1H, C*Ha*Hb-NH), 4.21 (dd, *J* = 5.7, 15.4 Hz, 1H, CHa*Hb*-NH). ^13^C NMR (DMSO-*d*_6_) δ: 169.65 (C, C=O), 162.1 (dd,^3^ *J*_CF_ = 12.1 Hz,^1^ *J*_CF_ = 246.1 Hz, C, C2–Ar), 160.32 (dd,^3^ *J*_CF_ = 12.1 Hz,^1^ *J*_CF_ = 251.4 Hz, C, C4–Ar), 152.0 (CH, triaz), 148.21 (C, Ar), 145.1 (CH, triaz), 139.7 (C, Ar), 137.2 (C, Ar), 137.7 (C, Ar), 130.96 (dd,^3^ *J*_CF_ = 5.2 Hz,^3^ *J*_CF_ = 9.8 Hz, CH, C6–Ar), 128.9 (CH × 2, Ar), 128.0 (CH × 2, Ar), 127.1 (CH × 2, Ar), 121.9 (CH × 2, Ar), 120.7 (dd,^4^ *J*_CF_ = 3.6 Hz, ^2^ *J*_CF_ = 15.3 Hz, CH, C1–Ar), 120.58 (q,^1^ *J*_CF_ = 256.1 Hz, CF_3_), 112.18 (dd,^4^ *J*_CF_ = 3.5 Hz, ^2^ *J*_CF_ = 21.3 Hz, CH, C5–Ar), 104.39 (t,^2^ *J*_CF_ = 26.0 Hz, CH, C3–Ar), 50.5 (CH_2_-triaz), 44.1 (CH), 42.3 (CH_2_–NH). ^19^F NMR (DMSO-*d*_6_) δ: −56.76 (CF_3_), −111.08 (*para*–F-Ar), −112.92 (*ortho*–F-Ar). Anal. Calcd for C_25_H_19_F_5_N_4_O_2_ (502.45): C 59.76%, H 3.81%, N 11.15%. Found: C 59.72%, H 3.69%, N 11.18%.

##### N-(2-(2,4-Difluorophenyl)-2-hydroxy-3-(1H-1,2,4-triazol-1-yl)­propyl)-4-nitrobenzamide (**13**)

Dichloromethane (12 mL) and sat. aqueous NaHCO_3_ (24 mL) were stirred vigorously and chilled in an ice bath. 4-Nitrobenzoyl chloride (**7**) (0.90 g, 4.89 mmol) was added, followed immediately by 1-amino-2-(2,4-difluorophenyl)-3-(1*H*-1,2,4-triazol-1-yl)­propan-2-ol (**4**) (0.83 g, 3.26 mmol). Stirring was continued at room temperature overnight. The solvent was evaporated under reduced pressure, and then the suspension was extracted with EtOAc (2 × 50 mL) and dried (MgSO_4_), and the organic layer was evaporated to give the crude product, which was purified by gradient column chromatography eluting with CH_2_Cl_2_-MeOH 97:3 v/v to afford the product as a white solid: Yield: 0.86 g (65%); mp 232–234 °C; TLC: CH_2_Cl_2_-MeOH 95:5 v/v, *R*_f_ 0.45; HPLC: 100% at R.T. 4.37 min. ^1^H NMR (DMSO-*d*_6_) δ: 8.79 (t, *J* = 6.0 Hz, 1H, NH), 8.33 (s, 1H, triaz), 8.29 (d, *J* = 9.0 Hz, 2H, Ar), 7.97 (d, *J* = 9.0 Hz, 2H, Ar), 7.75 (s, 1H, triaz), 7.41 (dd, *J* = 9.0, 15.9 Hz, 1H, Ar), 7.20–7.15 (m, 1H,Ar), 6.93 (ddd, *J* = 2.5, 8.4, 10.9 Hz, 1H, Ar), 6.15 (s, 1H, OH, ex), 4.73 (d, *J* = 14.4 Hz, 1H, C*Ha*Hb-triaz), 4.61 (d, *J* = 14.4 Hz, 1H, CHa*Hb*-triaz), 3.85 (dd, *J* = 6.6, 13.9 Hz, 1H, C*Ha*Hb-NH), 3.77 (dd, *J* = 5.7, 13.8 Hz, 1H, CHa*Hb*-NH). ^13^C NMR (DMSO-*d*_*6*_) δ: 166.23 (C, C=O), 162.30 (dd,^3^ *J*_CF_ = 12.6 Hz,^1^ *J*_CF_ = 243.1 Hz, C, C2–Ar),159.63 (dd,^3^ *J*_CF_ = 12.2 Hz,^1^ *J*_CF_ = 247.7 Hz, C, C4–Ar), 151.02 (CH, triaz), 149.51 (C, C-NO_2_), 145.44 (CH, triaz), 140.22 (C, Ar), 130.47 (dd,^3^ *J*_CF_ = 5.8 Hz,^3^ *J*_CF_ = 9.7 Hz, CH, C6–Ar), 129.26 (2 × CH, Ar), 125.48 (dd,^4^ *J*_CF_ = 3.5 Hz,^2^ *J*_CF_ = 13.0 Hz, C, C1–Ar), 123.92 (2 × CH, Ar),111.14 (dd, ^4^ *J*_CF_ = 3.0 Hz,^2^ *J*_CF_ = 20.5 Hz, CH, C5–Ar), 104.41 (t,^2^ *J*_CF_ = 28.0 Hz, CH, C3–Ar), 75.40 (C–OH), 55.43 (CH_2_–triaz), 47.01 (CH_2_–NH_2_). ^19^F NMR (DMSO-*d*_6_) δ: −106.73 (*para*–F-Ar), −112.02 (*ortho*–F-Ar). HRMS (ESI) *m*/*z* Calculated: 404.1170 [M + H]^+^, Found: 404.1173 [M + H]^+^.

#### General Method for Preparation of Amides **26** and **44–48**

To a cooled (0 °C, ice bath) solution of amine (**25** or **43**) (0.25 g, 0.66 mmol) in dry pyridine (5 mL) was added acylbenzoyl chloride (0.17 g, 1.0 mmol) in portions, and then, the reaction was stirred at room temperature overnight. The solvent was evaporated, and the resulting oil was extracted with EtOAc (50 mL) and washed with 1 M aq. HCl (25 mL), H_2_O (2 × 25 mL), and dried (MgSO_4_).

The organic layer was evaporated under reduced pressure, and the crude product was purified by gradient column chromatography.

##### 4-Chloro-*N*-(4-((2-(2,4-difluorophenyl)-2-hydroxy-3-(1*H*-1,2,4-triazol-1-yl)­propyl)­carbamoyl)­phenyl)­benzamide (**26**)

The product was prepared from 4-amino-*N*-(2-(2,4-difluorophenyl)-2-hydroxy-3-(1*H*-1,2,4-triazol-1-yl)­propyl)­benzamide (**25**) (0.25 g, 0.66 mmol) and 4-chlorobenzoyl chloride (0.17 g, 1.0 mmol). Purified using gradient chromatography eluting with CH_2_Cl_2_-MeOH 96:4 v/v to afford the product as a white solid: Yield: 0.26 g (78%); mp 211–213 °C; TLC: CH_2_Cl_2_-MeOH 95:5 v/v, *R*_f_ 0.37; HPLC: 100% at R.T. 4.60 min. ^1^H NMR (DMSO-*d*_6_) δ: 10.50 (s, 1H, NH), 8.48 (t, *J* = 6.0 Hz, 1H, NH), 8.34 (s, 1H, triaz), 7.99 (d, *J* = 8.8 Hz, 2H, Ar), 7.84 (d, *J* = 9.0 Hz, 2H, Ar), 7.78 (d, *J* = 9.0 Hz, 2H, Ar), 7.75 (s, 1H, triaz), 7.62 (d, *J* = 8.8 Hz, 2H, Ar), 7.42 (dd, *J* = 9.0, 15.9 Hz, 1H, Ar), 7.21–7.16 (m, 1H,Ar), 6.93 (ddd, *J* = 2.4, 8.4, 10.9 Hz, 1H, Ar), 6.34 (s, 1H, OH, ex), 4.71 (d, *J* = 14.4 Hz, 1H, C*Ha*Hb-triaz), 4.58 (d, *J* = 14.4 Hz, 1H, CHa*Hb*-triaz), 3.81 (dd, *J* = 6.1, 14.4 Hz, 1H, C*Ha*Hb-NH), 3.77 (dd, *J* = 5.7, 14.4 Hz, 1H, CHa*Hb*-NH). ^13^C NMR (DMSO-*d*_6_) δ: 167.73 (C, C=O), 165.14 (C, C=O), 162.27 (dd,^3^ *J*_CF_ = 13.4 Hz,^1^ *J*_CF_ = 246.8 Hz, C, C2–Ar),159.56 (dd,^3^ *J*_CF_ = 12.3 Hz,^1^ *J*_CF_ = 247.1 Hz, C, C4–Ar), 150.97 (CH, triaz), 145.45 (CH, triaz), 142.32 (C, Ar), 137.11 (C, Ar), 133.76 (C, Ar), 130.53 (dd,^3^ *J*_CF_ = 6.3 Hz,^3^ *J*_CF_ = 9.5 Hz, CH, C6–Ar), 130.17 (2 × CH, Ar), 129.19 (C, Ar), 128.98 (2 × CH, Ar), 128.58 (2 × CH, Ar), 125.29 (dd,^4^ *J*_CF_ = 3.4 Hz,^2^ *J*_CF_ = 13.0 Hz, C, C1–Ar), 119.91 (2 × CH, Ar),111.17 (dd, ^4^ *J*_CF_ = 3.3 Hz,^2^ *J*_CF_ = 20.7 Hz, CH, C5–Ar), 104.39 (t,^2^ *J*_CF_ = 27.9 Hz, CH, C3–Ar), 75.66 (C–OH), 55.59 (CH_2_–triaz), 47.17 (CH_2_–NH). ^19^F NMR (DMSO-*d*_6_) δ: −106.86 (*para*–F-Ar), −112.12 (*ortho*–F-Ar). HRMS (ESI) *m*/*z* Calculated: 511.1223/513.1223 (^35^Cl/^37^Cl) [M + H]^+^, Found: 512.1304/514.1284 (^35^Cl/^37^Cl) [M + H]^+^.

##### 4-Chloro-*N*-(4-((2-(2,4-difluorophenyl)-3-(1*H*-1,2,4-triazol-1-yl)­propanamido)­methyl)­phenyl)­benzamide (**44**)

The product was prepared from *N*-(4-aminobenzyl)-2-(2,4-difluorophenyl)-3-(1*H*-1,2,4-triazol-1-yl)­propenamide (**43**) (0.12 g, 0.33 mmol) and 4-chlorobenzoyl chloride (0.06 mL, 0.50 mmol). Purified using gradient chromatography eluting with CH_2_Cl_2_-MeOH 97:3 v/v to afford the product as a white solid: Yield: 0.11 g (68%); mp 206–208 °C; TLC: CH_2_Cl_2_-MeOH 95:5 v/v, *R*_f_ 0.50; HPLC: 100% at R.T. 4.57 min. ^1^H NMR (DMSO-*d*_6_) δ: 10.26 (s, 1H, NH), 8.73 (t, *J* = 5.9 Hz, 1H, NH), 8.36 (s, 1H, triaz), 7.98 (d, *J* = 8.8 Hz, 2H, Ar), 7.95 (s, 1H, triaz), 7.63 (d, *J* = 8.5 Hz, 2H, Ar), 7.60 (d, *J* = 8.8 Hz, 2H, Ar), 7.56 (dd, *J* = 8.7, 15.3 Hz, 1H, Ar), 7.23 (dd, *J* = 2.7, 9.4, 10.5 Hz, 1H, Ar), 7.10 (ddd, *J* = 2.4, 8.4, 11.5 Hz, 1H, Ar), 6.99 (d, *J* = 8.5 Hz, 2H, Ar), 4.81 (dd, *J* = 7.3, 12.3 Hz, 1H, C*Ha*Hb-triaz), 4.50–4.43 (m, 2H, C*H*CHa*Hb*-triaz), 4.25 (dd, *J* = 6.1, 15.2 Hz, 1H, C*Ha*Hb-NH), 4.14 (dd, *J* = 5.6, 15.2 Hz, 1H, CHa*Hb*-NH). ^13^C NMR (DMSO-*d*_6_) δ: 169.51 (C, C=O), 164.70 (C, C=O), 162.28 (dd,^3^ *J*_CF_ = 12.4 Hz,^1^ *J*_CF_ = 188.7 Hz, C, C2–Ar), 160.31 (dd,^3^ *J*_CF_ = 12.5 Hz, ^1^ *J*_CF_ = 190.4 Hz, C, C4–Ar), 151.98 (CH, triaz), 145.05 (CH, triaz), 138.06 (C, Ar), 136.82 (C, Ar), 134.76 (C, Ar), 134.01 (C, Ar), 130.95 (dd,^3^ *J*_CF_ = 5.2 Hz,^3^ *J*_CF_ = 9.54 Hz, CH, C6–Ar), 130.04 (CH × 2, Ar), 128.90 (CH × 2, Ar), 127.58 (CH × 2, Ar), 120.71­(CH × 2, Ar), 120.67 (dd,^4^ *J*_CF_ = 2.26 Hz, ^2^ *J*_CF_ = 12.2 Hz, CH, C1–Ar), 112.06 (dd,^4^ *J*_CF_ = 3.4 Hz, ^2^ *J*_CF_ = 21.0 Hz, CH, C5–Ar), 104.37 (t,^2^ *J*_CF_ = 26.0 Hz, CH, C3–Ar), 50.51 (CH_2_-triaz), 44.06 (CH), 42.22 (CH_2_–NH). ^19^F NMR (DMSO-*d*_6_) δ: −111.09 (*para*–F-Ar), −112.92 (*ortho*–F-Ar). HRMS (ESI) *m*/*z* Calculated: 518.1171 [M + Na]^+^, Found: 518.1169 [M + Na]^+^.

##### *N*-(4-((2-(2,4-Difluorophenyl)-3-(1*H*-1,2,4-triazol-1-yl)­propanamido)­methyl)­phenyl)-4-methoxybenzamide (**45**)

The product was prepared from *N*-(4-aminobenzyl)-2-(2,4-difluorophenyl)-3-(1*H*-1,2,4-triazol-1-yl)­propenamide (**43**) (0.15 g, 0.41 mmol) and 4-methoxybenzoyl chloride (0.08 mL, 0.62 mmol). Purified using gradient chromatography eluting with CH_2_Cl_2_-MeOH 97:3 v/v to afford the product as a white solid: Yield: 0.13 g (72%); mp 222–224 °C; TLC: CH_2_Cl_2_-MeOH 95:5 v/v, *R*_f_ 0.40; HPLC: 100% at R.T. 4.44 min. ^1^H NMR (DMSO-*d*_6_) δ: 10.04 (s, 1H, NH), 8.72 (t, *J* = 5.9 Hz, 1H, NH), 8.36 (s, 1H, triaz), 7.99–7.93 (m, 3H, triaz + Ar), 7.63 (d, *J* = 8.6 Hz, 2H, Ar), 7.57 (dd, *J* = 8.7, 15.3 Hz, 1H, Ar), 7.23 (ddd, *J* = 2.7, 9.4, 10.5 Hz, 1H, Ar), 7.10 (ddd, *J* = 2.4, 8.4, 10.7 Hz, 1H, Ar), 7.05 (d, *J* = 8.9 Hz, 2H, Ar), 6.97 (d, *J* = 8.5 Hz, 2H, Ar), 4.81 (dd, *J* = 7.2, 12.1 Hz, 1H, C*Ha*Hb-triaz), 4.50- 4.43 (m, 2H, C*H*CHa*Hb*-triaz), 4.24 (dd, *J* = 6.1, 15.2 Hz, 1H, C*Ha*Hb-NH), 4.14 (dd, *J* = 5.6, 15.2 Hz, 1H, CHa*Hb*-NH), 3.84 (s, 3H, CH_3_). ^13^C NMR (DMSO-*d*_6_): δ 169.49 (C, C=O), 165.17 (C, C=O), 162.32 (C, Ar), 162.28 (dd,^3^ *J*_CF_ = 12.2 Hz,^1^ *J*_CF_ = 188.0 Hz, C, C2–Ar), 160.32 (dd,^3^ *J*_CF_ = 12.4 Hz,^1^ *J*_CF_ = 190.0 Hz, C, C4–Ar), 151.98 (CH, triaz), 145.05 (CH, triaz), 138.46 (C, Ar), 134.29 (C, Ar), 130.96 (dd,^3^ *J*_CF_ = 5.0 Hz,^3^ *J*_CF_ = 9.8 Hz, CH, C6–Ar), 129.99 (CH × 2, Ar), 127.53 (CH × 2, Ar), 127.34 (C, Ar), 120.70 (dd,^4^ *J*_CF_ = 3.6 Hz,^2^ *J*_CF_ = 14.8 Hz, CH, C1–Ar), 120.62 (CH × 2, Ar), 114.03 (CH × 2, Ar), 112.14 (dd,^4^ *J*_CF_ = 3.4 Hz,^2^ *J*_CF_ = 21.1 Hz, CH, C5–Ar), 104.36 (t,^2^ *J*_CF_ = 26.5 Hz, CH, C3–Ar), 55.88 (OCH_3_), 50.52 (CH_2_-triaz), 44.07 (CH), 42.23 (CH_2_–NH). ^19^F NMR (DMSO-*d*_6_) δ: −111.10 (*para*–F-Ar), −112.92 (*ortho*–F-Ar). HRMS (ESI) *m*/*z* Calculated: 514.1666 [M + Na]^+^, Found: 514.1660 [M + Na]^+^.

##### 4-Cyano-*N*-(4-((2-(2,4-difluorophenyl)-3-(1*H*-1,2,4-triazol-1-yl)­propanamido)­methyl) phenyl)­benzamide (**46**)

The product was prepared from *N*-(4-aminobenzyl)-2-(2,4-difluorophenyl)-3-(1*H*-1,2,4-triazol-1-yl)­propenamide (**43**) (0.16 g, 0.44 mmol) and 4-cyanobenzoyl chloride (0.11 g, 0.67 mmol. Purified using gradient chromatography eluting with CH_2_Cl_2_-MeOH 97:3 v/v to afford the product as a white solid: Yield: 0.17 g (80%); mp 200–201 °C; TLC: CH_2_Cl_2_-MeOH 95:5 v/v, *R*_f_ 0.32; HPLC: 100% at R.T. 4.38 min. ^1^H NMR (DMSO-*d*_6_) δ: 10.44 (s, 1H, NH), 8.73 (t, *J* = 5.9 Hz, 1H, NH), 8.36 (s, 1H, triaz), 8.10 (d, *J* = 8.7 Hz, 2H, Ar), 8.02 (d, *J* = 8.7 Hz, 2H, Ar), 7.64 (d, *J* = 8.6 Hz, 2H, Ar), 7.57 (dd, *J* = 8.7, 15.2 Hz, 1H, Ar), 7.23 (ddd, *J* = 2.7, 9.4, 10.5 Hz, 1H, Ar), 7.10 (ddd, *J* = 2.4, 8.4, 11.6 Hz, 1H, Ar), 7.00 (d, *J* = 8.7 Hz, 2H, Ar), 4.81 (dd, *J* = 7.4, 12.4 Hz, 1H, C*Ha*Hb-triaz), 4.50- 4.43 (m, 2H, C*H*CHa*Hb*-triaz), 4.25 (dd, *J* = 6.1, 15.2 Hz, 1H, C*Ha*Hb-NH), 4.14 (dd, *J* = 5.6, 15.2 Hz, 1H, CHa*Hb*-NH). ^13^C NMR (DMSO-*d*_6_): δ 169.53 (C, C=O), 164.42 (C, C=O), 162.28 (dd,^3^ *J*_CF_ = 12.5 Hz, ^1^ *J*_CF_ = 188.8 Hz, C, C2–Ar), 160.32 (dd,^3^ *J*_CF_ = 12.7 Hz, ^1^ *J*_CF_ = 191.4 Hz, C, C4–Ar), 151.98 (CH, triaz), 145.06 (CH, triaz), 139.33 (C, Ar), 137.83 (C, Ar), 135.08 (C, Ar), 132.91 (CH × 2, Ar), 130.96 (dd,^3^ *J*_CF_ = 5.1 Hz, ^3^ *J*_CF_ = 9.8 Hz, CH, C6–Ar), 128.95 (CH × 2, Ar), 127.63 (CH × 2, Ar), 120.76 (CH × 2, Ar), 120.67 (dd,^4^ *J*_CF_ = 5.4 Hz, ^2^ *J*_CF_ = 16.4 Hz, CH, C1–Ar), 118.78 (CN), 114.27 (C, Ar), 112.15 (dd,^4^ *J*_CF_ = 3.4 Hz, ^2^ *J*_CF_ = 21.0 Hz, CH, C5–Ar), 104.37 (t,^2^ *J*_CF_ = 26.3 Hz, CH, C3–Ar), 50.52 (CH_2_-triaz), 44.07 (CH), 42.21 (CH_2_–NH). ^19^F NMR (DMSO-*d*_6_) δ: −111.08 (*para*–F-Ar), −112.92 (*ortho*–F-Ar). HRMS (ESI) *m*/*z* Calculated: 509.1513 [M + Na]^+^, Found: 509.1509 [M + Na]^+^.

##### *N*-(4-((2-(2,4-Difluorophenyl)-3-(1*H*-1,2,4-triazol-1-yl)­propanamido)­methyl)­phenyl)­nicotinamide (**47**)

The product was prepared from *N*-(4-aminobenzyl)-2-(2,4-difluorophenyl)-3-(1*H*-1,2,4-triazol-1-yl)­propenamide (**43**) (0.14 g, 0.39 mmol) and nicotynoyl chloride hydrochloride (0.10 g, 0.58 mmol). Purified using gradient chromatography eluting with CH_2_Cl_2_-MeOH 96:4 v/v to afford the product as a white solid: Yield: 0.14 g (77%); mp 204–206 °C; TLC: CH_2_Cl_2_-MeOH 95:5 v/v, *R*_f_ 0.27; HPLC: 100% at R.T. 4.23 min. ^1^H NMR (DMSO-*d*_6_) δ: 10.39 (s, 1H, NH), 9.10 (d, *J* = 2.3 Hz, 1H, CH-pyridine), 8.76 (d, *J* = 1.6, 4.7 Hz, 1H, CH-pyridine), 8.73 (t, *J* = 5.9 Hz, 1H, NH), 8.36 (s, 1H, triaz), 8.28 (tt, *J* = 1.7, 2.3 Hz, 1H, CH-pyridine), 7.95 (s, 1H, triaz), 7.64 (d, *J* = 8.6 Hz, 2H, Ar), 7.60–7.55 (m, 2H, Ar), 7.23 (ddd, *J* = 2.7, 9.4, 10.4 Hz, 1H, Ar), 7.10 (ddd, *J* = 2.4, 8.4, 10.9 Hz, 1H, Ar), 7.01 (d, *J* = 8.6 Hz, 2H, Ar), 4.81 (dd, *J* = 7.3, 12.3 Hz, 1H, C*Ha*Hb-triaz), 4.51–4.43 (m, 2H, C*H*CHa*Hb*-triaz), 4.25 (dd, *J* = 6.6, 15.2 Hz, 1H, C*Ha*Hb-NH), 4.15 (dd, *J* = 5.6, 15.2 Hz, 1H, CHa*Hb*-NH). ^13^C NMR (DMSO-*d*_6_): δ 169.53 (C, C=O), 164.33 (C, C=O), 162.29 (dd,^3^ *J*_CF_ = 12.3 Hz, ^1^ *J*_CF_ = 188.7 Hz, C, C2–Ar), 160.32 (dd,^3^ *J*_CF_ = 12.6 Hz, ^1^ *J*_CF_ = 190.6 Hz, C, C4–Ar), 152.54 (CH, pyridine), 151.98 (CH, triaz), 149.10 (CH, pyridine), 145.06 (CH, triaz), 137.93 (C, Ar), 135.86 (CH, pyridine), 134.93 (C, Ar), 130.96 (dd,^3^ *J*_CF_ = 5.2 Hz, ^3^ *J*_CF_ = 9.9 Hz, CH, C6–Ar), 130.95 (C, Ar), 127.63 (CH × 2, Ar), 123.93 (CH, pyridine), 120.69 (dd,^4^ *J*_CF_ = 3.9 Hz, ^2^ *J*_CF_ = 10.0 Hz, CH, C1–Ar), 120.68 (CH × 2, Ar), 112.14 (dd,^4^ *J*_CF_ = 3.4 Hz, ^2^ *J*_CF_ = 21.0 Hz, CH, C5–Ar), 104.37 (t,^2^ *J*_CF_ = 26.2 Hz, CH, C3–Ar), 50.52 (CH_2_-triaz), 44.07 (CH), 42.21 (CH_2_–NH). ^19^F NMR (DMSO-*d*_*6*_) δ: −111.09 (*para*–F-Ar), −112.92 (*ortho*–F-Ar). HRMS (ESI) *m*/*z* Calculated: 463.1703 [M + H]^+^, Found: 463.1693 [M + H]^+^.

##### *N*-(4-((2-(2,4-Difluorophenyl)-3-(1*H*-1,2,4-triazol-1-yl)­propanamido)­methyl)­phenyl)­pyrazine-2-carboxamide (**48**)

The product was prepared from *N*-(4-aminobenzyl)-2-(2,4-difluorophenyl)-3-(1*H*-1,2,4-triazol-1-yl)­propenamide (**43**) (0.15 g, 0.41 mmol) and pyrazine-2-carbonyl chloride (0.8 mmol), prepared *in situ* by addition of SOCl_2_ (0.11 mL, 1.61 mmol) to an ice-cooled solution of pyrazine-2-carboxylic acid (0.1 g, 0.8 mmol) in CH_2_Cl_2_ (10 mL) followed by heating at 40 °C for 4 h. Purified using gradient chromatography eluting with CH_2_Cl_2_-MeOH 97:3 v/v to afford the product as a white solid: Yield: 0.13 g (68%); mp 202–204 °C; TLC: CH_2_Cl_2_-MeOH 95:5 v/v, *R*_f_ 0.40; HPLC: 100% at R.T. 4.29 min. ^1^H NMR (DMSO-*d*_6_) δ: 10.68 (s, 1H, NH), 9.29 (d, *J* = 1.5 Hz, 1H, CH-pyrazine), 8.93 (d, *J* = 2.5 Hz, 1H, CH-pyrazine), 8.81 (dd, *J* = 1.5, 2.5 Hz, 1H, CH-pyrazine), 8.73 (t, *J* = 5.9 Hz, 1H, NH), 8.36 (s, 1H, triaz), 7.95 (s, 1H, triaz), 7.77 (d, *J* = 8.6 Hz, 2H, Ar), 7.56 (dd, *J* = 8.7, 15.3 Hz, 1H, Ar), 7.23 (ddd, *J* = 2.7, 9.4, 10.4 Hz, 1H, Ar), 7.10 (ddd, *J* = 2.4, 8.4, 10.9 Hz, 1H, Ar), 7.02 (d, *J* = 8.6 Hz, 2H, Ar), 4.81 (dd, *J* = 6.5, 11.5 Hz, 1H, C*Ha*Hb-triaz), 4.50- 4.44 (m, 2H, C*H*CHa*Hb*-triaz), 4.25 (dd, *J* = 6.1, 15.2 Hz, 1H, C*Ha*Hb-NH), 4.15 (dd, *J* = 5.6, 15.2 Hz, 1H, CHa*Hb*-NH). ^13^C NMR (DMSO-*d*_6_): δ 169.53 (C, C=O), 162.29 (dd,^3^ *J*_CF_ = 12.6 Hz, ^1^ *J*_CF_ = 187.1 Hz, C, C2–Ar), 162.02 (C, C=O), 160.32 (dd,^3^ *J*_CF_ = 12.1 Hz, ^1^ *J*_CF_ = 189.8 Hz, C, C4–Ar), 151.98 (CH, triaz), 148.13 (CH, pyrazine), 145.51 (C, Ar), 145.05 (CH, triaz), 144.47 (CH, pyrazine), 143.67 (CH, pyrazine), 137.26 (C, Ar), 135.22 (C, Ar), 130.96 (dd,^3^ *J*_CF_ = 5.1 Hz,^3^ *J*_CF_ = 9.6 Hz, CH, C6–Ar), 127.67 (CH × 2, Ar), 120.85 (CH × 2, Ar), 120.70 (dd,^4^ *J*_CF_ = 2.26 Hz, ^2^ *J*_CF_ = 12.2 Hz, CH, C1–Ar), 112.14 (dd,^4^ *J*_CF_ = 3.4 Hz, ^2^ *J*_CF_ = 21.2 Hz, CH, C5–Ar), 104.36 (t,^2^ *J*_CF_ = 26.4 Hz, CH, C3–Ar), 50.51 (CH_2_-triaz), 44.07 (CH), 42.24 (CH_2_–NH). ^19^F NMR (DMSO-*d*_6_) δ: −111.09 (*para*–F-Ar), −112.93 (*ortho*–F-Ar). HRMS (ESI) *m*/*z* Calculated: 464.1646 [M + H]^+^, Found: 464.1642 [M + H]^+^.

### Antifungal Assays

Details for the *Saccharomyces cerevisiae* and Candida laboratory strains used in this study are provided in Table S1.

#### Disk Diffusion Assay

The susceptibilities of *S. cerevisiae* and Candida species strains to azole compounds were observed as zones of growth inhibition in agarose diffusion assays.^49^ The disk diffusion assays were carried out as described by Keniya et al.^50^ Complete supplement mixture (CSM) agarose (0.6% agarose [wt/vol]; 20 mL of synthetic defined medium (SD); pH 6.8–7) was solidified in a rectangular Petri dish which was overlaid with CSM agarose (0.6% [wt/vol]; 5 mL; pH 6.8–7) seeded with yeast cells at an optical density (OD600) of 0.008 (118,000 cells per 1 mL of overlay). Azole compounds (10 nmol/disk) were applied to sterile BBL paper disks (Becton Dickinson Co., Sparks, MD) and placed on solidified overlays. Cell growth was assessed after incubation at 30 °C for 48 h.

#### MIC_80_ Determination for S. cerevisiae and C. albicans Laboratory Strains

The MIC assays were carried out using a modification of the NCLS method as described by Keniya et al.^18,50^ MIC_80_s for novel inhibitors, MCF and PCZ, were determined in 96-well microtiter plates using SD buffered to pH 6.8 for *S. cerevisiae* constructs and at pH 7 for *C. albicans*. Cells were seeded at an OD_600 nm_ of 0.005 (1.5 × 10^4^ CFU), and the plates were incubated at 30 °C with shaking at 200 rpm for 48 h for *S. cerevisiae* strains and 24 h for *C. albicans*. Cell growth was assessed by measuring the OD_600 nm_ using a Synergy 2 multimode plate reader (BioTek Instruments, VT, USA). Each MIC_80_ was determined using triplicate measurements for pools of 4 clones of each strain in three separate experiments.

### Measurement of Reconstituted CYP51 Activity

The reconstituted CYP51 assay previously described^12^ contained 1 μM CaCyp51, 2 μM *Homo sapiens* cytochrome P450 reductase and 50 μM lanosterol in a final volume of 500 μL. After the addition of azole in DMSO (2.5 μL), reactions were incubated at 37 °C for 10 min prior to initiation by the addition of 100 μL 20 mM β-NADPH-Na_4_. Sterol substrates and products were extracted with ethyl acetate, derivatized with N,O-bis­(trimethylsilyl)­trifluoroacetamide (BSTFA)–trimethylchlorosilane (TMCS) (99:1) in the presence of anhydrous pyridine, and analyzed by GC/MS as described previously.^51^ IC_50_ determinations were performed in duplicate. Nonlinear regression of inhibitor concentration vs normalized response was calculated by GraphPad Prism 10.4.0 for Windows (GraphPad Software, Boston, Massachusetts, USA, www.graphpad.com).

### Viability (CellTiter Blue) Assay

Human epithelial breast cells, MCF-10A, were routinely cultured in DMEM/F-12 (Fisher Scientific, Loughborough, UK) containing 5% heat-inactivated horse serum (ThermoFisher Scientific, UK), 20 ng/mL hEGF (Merck Life Science, Dorset, UK), 500 ng/mL hydrocortisone (Merck Life Science, Dorset, UK) and 10 μg/mL insulin (Merck Life Science, Dorset, UK) under tissue culture conditions (37 °C, 5% CO_2_ in a humidified incubator). Cells were passaged upon reaching confluency using 0.25% Trypsin/EDTA (Fisher Scientific, Loughborough, UK) for a maximum of 15 passages from defrosting. (Merck Life Science, Dorset, UK).

Cells were seeded in 100 μL of complete medium per well in a black, flat-bottomed, 96-well tissue-culture-treated plate (Fisher Scientific, Loughborough, UK) at a density of 4000 cells/well and incubated under tissue culture conditions for 24 h. Cells were then treated by mixing in an additional 100 μL of drug/diluent control in complete medium to give final concentrations of 10 μM–1 pM. DMSO was used as a vehicle control, and as a positive toxic control, staurosporine was added at a final concentration of 10 μM. Cells were then incubated for 48 h under tissue culture conditions before the treatments were replaced with fresh media before performing the CellTiter Blue assay^38^ (Promega, Southampton, UK) was performed following the manufacturer's instructions. Fluorescence intensity was measured using a plate reader (Tecan, Theale, UK), and the data were displayed using GraphPad Prism. Three independent experiments were performed, with data points for each experiment performed in quadruplicate.

#### X-ray Crystallography of ScCYP51 in Complex with Compound **22**

Ni-NTA affinity and SEC-purified ScCyp51–6× His was cocrystallized with **22** using a hanging-drop vapor diffusion method at 18 °C.^52^ The reservoir solution contained 44% PEG 400 (Sigma-Aldrich) and 0.1 M glycine-NaOH buffer pH 9.5. The drop volume was 1 or 2 μL in a 1:1 ratio of reservoir solution and 20 to 30 mg/mL of protein in SEC buffer with 40 μM of racemic compound **22**. Crystals were picked by using an appropriately sized nylon loop (MiTeGen, Ithaca, NY, USA) and flash-cooled in liquid nitrogen. Data sets were collected on the MX2 beamline at the Australian Synchrotron using a Dectris EIGER 16 M detector. The data were indexed and integrated using XDS^53^ and scaled using AIMLESS in the CCP4 program suite.^54^ Molecular replacement was carried out using Phaser-MR^55^ from Phenix^56^ using the structure of ScCYP51 in complex with lanosterol (PDB ID 4LXJ) as the template. Structure refinement and modeling were carried out in Phenix.refine^56^ and Coot,^57^ respectively. The ligand *S-* **22** was generated from the Grade Web Server^58^ and was modeled into the appropriate density in the active site. Water molecules were added to densities if at least one hydrogen bond was detected (2.5 to 3.3 Å). Figures were generated by using PyMOL (Schrodinger). See the Supporting Information (Table S2) for data collection and refinement statistics.

## Supplementary Material













## Notes

Table of Saccharomyces cerevisiae recombinant strains and Candida clinical isolates used in this research (Table S1); additional figures illustrating susceptibilities of yeast constructs and clinical isolates to compounds (Figures S1–S4); data collection and refinement statistics for ScCYP51 in complex with **22** (Table S2); omit map showing only the *S-* **22** enantiomer (white) complexed with ScCYP51 (Figure S5). IC_50_ determinations of fluconazole (FLC) and compound **22** against wild type and resistant Candida strains (Figure S6); distortion of the haem after 150 ns molecular dynamics simulation of the CaCYP51 Y132H–K143R protein; 3D image of *R*-**22** and *S*-**22** in CaCYP51 wild-type after 150 ns MD simulation as well as 2D ligand-binding interaction graphs and 3D images showing interactions of *S*-**22** over the 150 ns MD simulation with wild-type and mutant Candida strains (Figures S9 and S10); methods for the synthesis of intermediate compounds and computational studies; and ^1^H, ^13^C, ^19^F NMR and HPLC or elemental analysis of final compounds (PDF)
Molecular formula strings of compounds (CSV)
CaCYP51_Y132H_S22_after MD (PDB)
CaCYP51_Y132H_K143R_S22_after MD (PDB)
CaCYP51_Y132F_S22_after MD (PDB)
CaCYP51_K143R_S22_after MD (PDB)

## Notes

PDB code for ScCYP51 with bound (*S*)-**22** is 8VK6.

## ⊥

Department of Pharmacognosy & Pharmaceutical Chemistry, College of Pharmacy, Taibah University, Medina 42353, Saudi Arabia

## #

Center for Discovery and Innovation, Hackensack Meridian Health, Nutley, New Jersey 07110, United States

## ∇

Department of Biological Science, University of California Los Angeles, Los Angeles, California 90095, United States

## Abbreviations Used

(CaCYP51): C. albicans CYP51
(CDI): carbonyldiimidazole
(CPME): cyclopentylmethyl ether
(DMF): *N*,*N*-dimethylformamide
(FLC): fluconazole
(IFI): invasive fungal infection
(ITC): itraconazole
(MCF): micafungin
(MIC): minimum inhibitory concentration
(MD): molecular dynamics
(OE): overexpressing
(PCZ): posaconazole
(ScCYP51): *S. cerevisiae* CYP51
(CYP51): sterol 14α-demethylase
(TMSOI): tetramethylsulfoxonium iodide
(VCZ): voriconazole
