# Supplementary material for: Exploring Long Arm Amide-Linked Side Chains in the Design of Antifungal Azole Inhibitors of Sterol 14α-Demethylase (CYP51)
Source: J Med Chem. 2025 May 22;68(11):10781–99. doi: 10.1021/acs.jmedchem.4c02922 (PMC12169614; doi:10.1021/acs.jmedchem.4c02922)
Supplement: Supplementary file 1 [file jm4c02922_si_001.pdf]

Supporting information

Exploring long arm amide-linked side chains in the design of antifungal azole inhibitors of sterol 14α-demethylase (CYP51)

Marwa Alsulaimany[a†], Mikhail V. Keniya[b†], Rehab S. Alanazi[a], Yasmeen N. Ruma[b†], Carwyn S. Hughes[a], Arwyn T. Jones[a], Joel D. A. Tyndall[c], Josie E. Parker[d], Brian C. Monk[b], Claire Simons[a*]

[a]*School of Pharmacy and Pharmaceutical Sciences, Cardiff University, King Edward VII Avenue, Cardiff CF10 3NB, UK.*
[b]*Faculty of Dentistry, Sir John Walsh Research Institute, University of Otago, Dunedin 9016, New Zealand*
[c]*School of Pharmacy, University of Otago, Dunedin 9054, New Zealand*
[d]*School of Biosciences, Cardiff University, Museum Avenue, Cardiff CF10 3AX, UK*

Corresponding Author *E-mail: simonsc@cardiff.ac.uk

**Table S1. (A)** *Saccharomyces cerevisiae* recombinant strains and (**B**) *Candida* clinical isolates	S2
**Figure S1**. Susceptibilities of yeast constructs and clinical isolates to compounds **11-16**	S3
**Figure S2**. Susceptibilities of yeast constructs and clinical isolates to compounds **21-24** and **26**	S4
**Figure S3**. Susceptibilities of yeast constructs and clinical isolates to compounds **37-40**	S5
**Figure S4**. Susceptibilities of yeast constructs and clinical isolates to compounds **44-48**	S6
**Table S2.** Data collection and refinement statistics for ScCYP51 in complex with **22**.	S7
**Figure S5**: Omit map showing only the *S*-**22** enantiomer (white) complexed with ScCYP51	S8
**Figure S6**. IC_{50} determinations of fluconazole (FLC) and compound **22**. IC_{50}s were determined for wild type CaCYP51, CaCYP51 Y132F and CaCYP51 Y132H + K143R with FLC (squares) and compound **22** (circles). Assays were performed in duplicate. Data are presented as normalised mean points with standard error of the mean error bars.	S9
**Figure S7.** Distortion of the haem after 150 ns molecular dynamics simulation of the CaCYP51 Y132H-K143R protein. The Fe (brown sphere) moves out of plane of the protoporphyrin ring (grey), resulting in the loss of two hydrogen bonds between the Fe and two of the four pyrroles in the protoporphyrin ring.	S10
**Figure S8**. Three-dimensional image of *R*-**22** and *S*-**22** in CaCYP51 wild-type [Haem in orange, H_2O shown as red spheres] after 150 ns MD simulation.	S11
**Figure S9**. 2D ligand interactions after 150 ns MD simulation and ligand-binding interaction graph showing interactions of *S*-**22** over the 150 ns MD simulation with (a) CaCYP51 WT, single amino acid mutant (b) CaCYP51 Y132F (c) CaCYP51 Y132H (d) CaCYP51 K143R, and double mutant (e) CaCYP51 Y132H K143R.	S12
**Figure S10**. 3D image of interactions of *S*-**22** with (a) CaCYP51 WT, single amino acid mutant (b) CaCYP51 Y132F (c) CaCYP51 Y132H (d) CaCYP51 K143R, and double mutant (e) CaCYP51 Y132H + K143R, after 150 ns MD simulation	S13
Methods for synthesis of intermediates and computational modelling	S14-S27
^{1}H, ^{13}C (APT), ^{19}F NMR and HPLC or elemental analysis of final compounds	S28-S48
References	S49-S50

Table S1. (A) *Saccharomyces cerevisiae* recombinant strains and (**B**) *Candida* clinical isolates

(A) ***S. cerevisiae*** **strains**

MBL Yeast Collection #	Genotype	Ref
**Host strains**		
Y85 AD1-8	*MAT-α PDR1-3, ura3, his1, Δyor1::hisG, Δsnq2::hisG, Δpdr10::hisG, Δpdr11::hisG, Δycf::hisG, Δpdr3::hisG, Δpdr15::hisG, Δpdr5::hisG*	1
Y663 ADΔ	*MAT-α PDR1-3, Δura3::dpl200, his1, Δyor1::hisG, Δsnq2::hisG, Δpdr10::hisG, Δpdr11::hisG, Δycf::hisG, Δpdr3::hisG, Δpdr15::hisG, Δpdr5::hisG*	2
Y1857 ADΔΔ	*MAT-α PDR1-3, Δura3::dpl200, Δhis1::dpl200, Δyor1::hisG, Δsnq2::hisG, Δpdr10::hisG, Δpdr11::hisG, Δycf::hisG, Δpdr3::hisG, Δpdr15::hisG, Δpdr5::hisG*	3
**Strains used in study**		
Y2411	AD2Δ, *Δpdr5::ura3*	6
Y2300	AD2Δ *Δpd5::ScErg11-6xHis ura3, ΔScERG11::his1*	3
Y2301	AD2Δ *Δpd5::ScErg11-6xHis Y140F ura3, ΔScERG11::his1*	4
Y2513	*AD2Δ Δpd5::ScErg11-6xHis Y140H ura3, ΔScERG11::his1*	5
Y2374	*ADΔ Δpd5::CgERG11-6xHis ura3, ΔScERG11:his1*	6
Y2459	AD2Δ *Δpdr5::CaERG11-6xHis, Δpdr15::CaNCP1A-6xHIS, ΔERG11::LoxP-ura3*	6
Y2711	AD2Δ *Δpdr5::CnCYP51-6xHis; Δpdr15::PDR5 prom-CnCPR-ura3, ΔScERG11::his1*	7
Y2716	AD2Δ *Δpdr5::CpCYP51-6×His Y132F, Δpdr15::PDR5 prom-CpCPR-6×His, ΔScERG11::his1*	8
Y2721	AD2Δ *Δpdr5::CpCYP51-6×His, Δpdr15::PDR5 prom-CpCPR-6×His, ΔScCYP51::HIS1*	8
Y2765	AD2Δ *Δpdr5::CauMDR1-6xHis his1*	9
Y2766	AD2Δ *Δpdr5::CauCDR1-6xHis his1*	9
Y2767	AD2Δ *Δpdr5::CauERG11-6xHis his1, ΔScERG11::ura3*	9
Y2768	AD2Δ *Δpdr5::CauERG11 Y132Fx6His his1, ΔScERG11::ura3*	9
Y2769	AD2Δ *Δpdr5::CauERG11::K143R-6xHis his1, ΔScERG11::ura3*	9
Y433	AD1-8 *Δpdr5::CgCDR1-1B*	10
Y525	AD1-8 *Δpdr5::CaMDR1*	1
Y570	AD1-8 *Δpdr5::CaCaCDR1*	1
Y2649	AD2Δ *Δpdr5::RaCYP51-6xHIS-F1 isoform URA3; Δpdr15::PDR5 prom-RaCPR-6xHIS, ΔScERG11::LoxP-HIS1*	11
Y2651	AD2Δ *Δpdr5::RaCYP51-6xHIS -F5 isoform URA3; Δpdr15::RaCPR-6xHIS, ΔScERG11::LoxP-HIS1*	11
Y2746	*AD2Δ pdr5::AfCyp51A-6xHis-URA3, PDR15::PDR5prom-AfCprA2-LoxP, ΔERG11:: AfErg6xFLAG-LoxP HIS1*	12
Y2747	*AD2Δ pdr5::AfCyp51B-6xHis-URA3, PDR15::PDR5prom-AfCprA2-LoxP, ΔERG11:: AfErg6xFLAG-LoxP HIS1*	12

(B) ***Candida*** **clinical isolates**

C. albicans	**Published designations**	**Ref**
Y71	SC5314	13
Y1		
Y610	FHB1 (TL1)	14
Y611	FHB3 (TL3)	14
C. glabrata		
Y99	CBS138	15
Y853	ATCC90030	

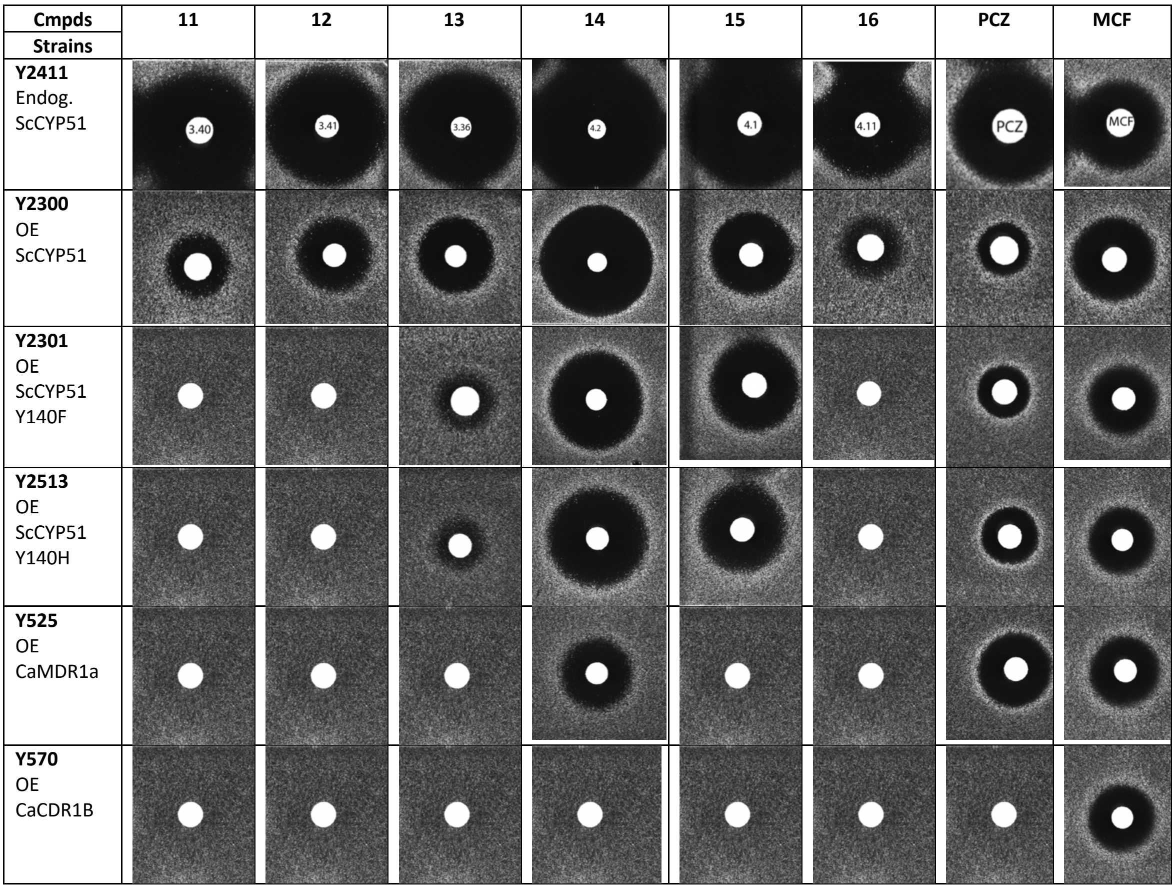

Figure S1. Susceptibilities of yeast constructs and clinical isolates to compounds **11-16**
(Y2411 – azole sensitive, Y2300 ERG11 overexpressed, Y2301 Y140F mutant, Y2513 Y140H mutant, Y525 Ca MDR1a MFS, Y570 CaCDR1B ABC. PCZ posaconazole, MCF micafungin.)

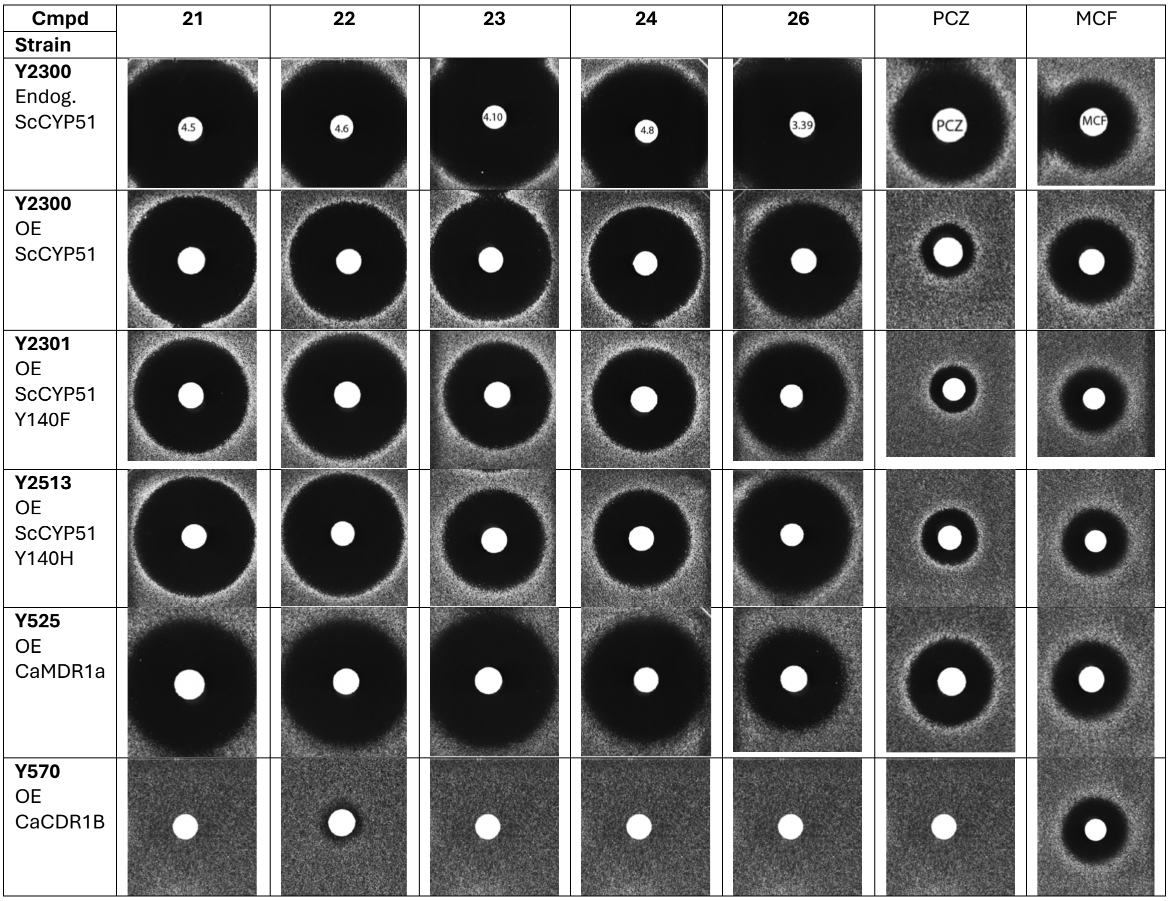

Figure S2. Susceptibilities of yeast constructs and clinical isolates to compounds **21-24** and **26** (Y2411 – azole sensitive, Y2300 ERG11 overexpressed, Y2301 Y140F mutant, Y2513 Y140H mutant, Y525 Ca MDR1a MFS, Y570 CaCDR1B ABC. PCZ posaconazole, MCF micafungin.)

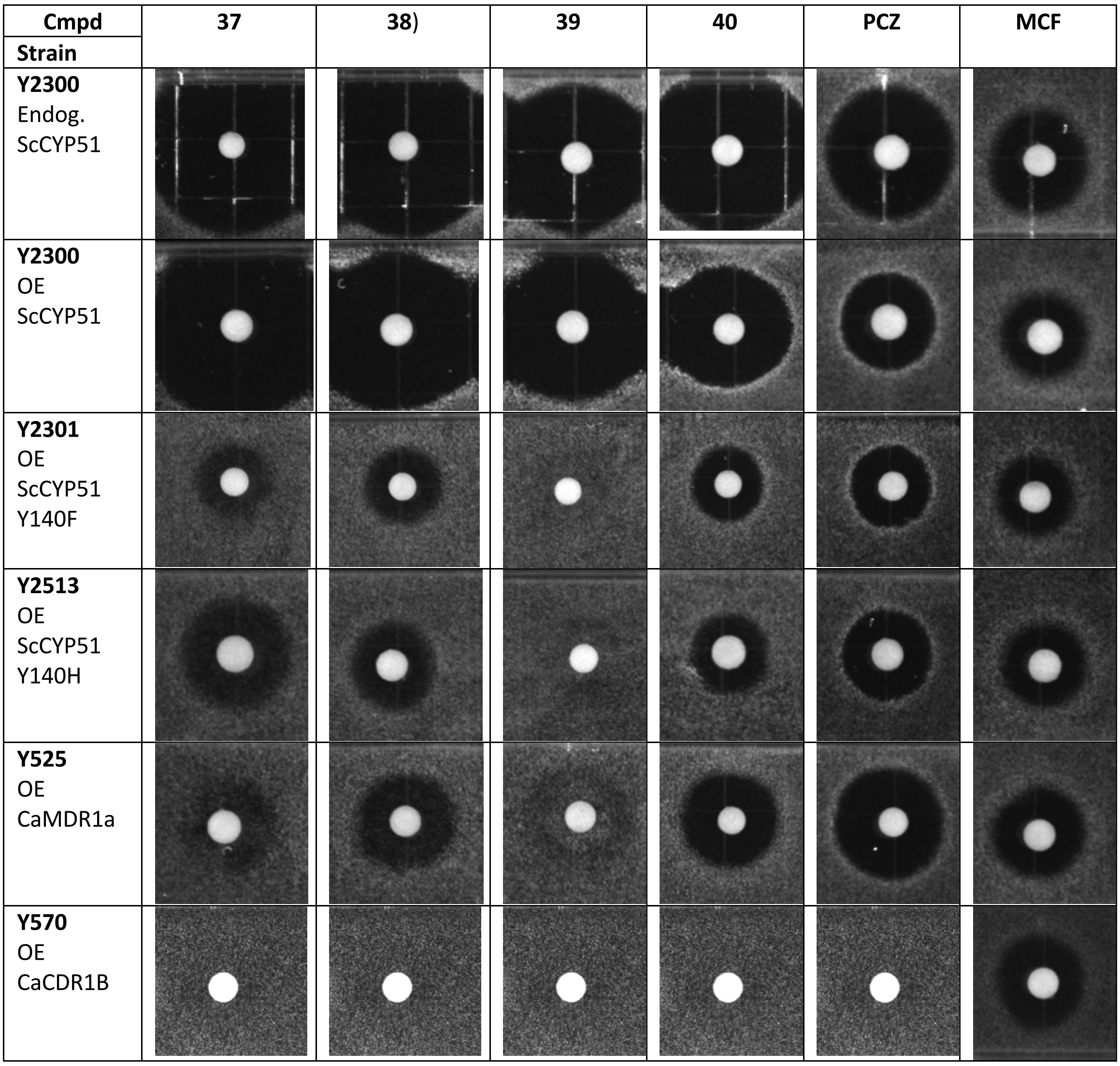

Figure S3. Susceptibilities of yeast constructs and clinical isolates to compounds **37-40** (Y2411 – azole sensitive, Y2300 ERG11 overexpressed, Y2301 Y140F mutant, Y2513 Y140H mutant, Y525 Ca MDR1a MFS, Y570 CaCDR1B ABC. PCZ posaconazole, MCF micafungin.)

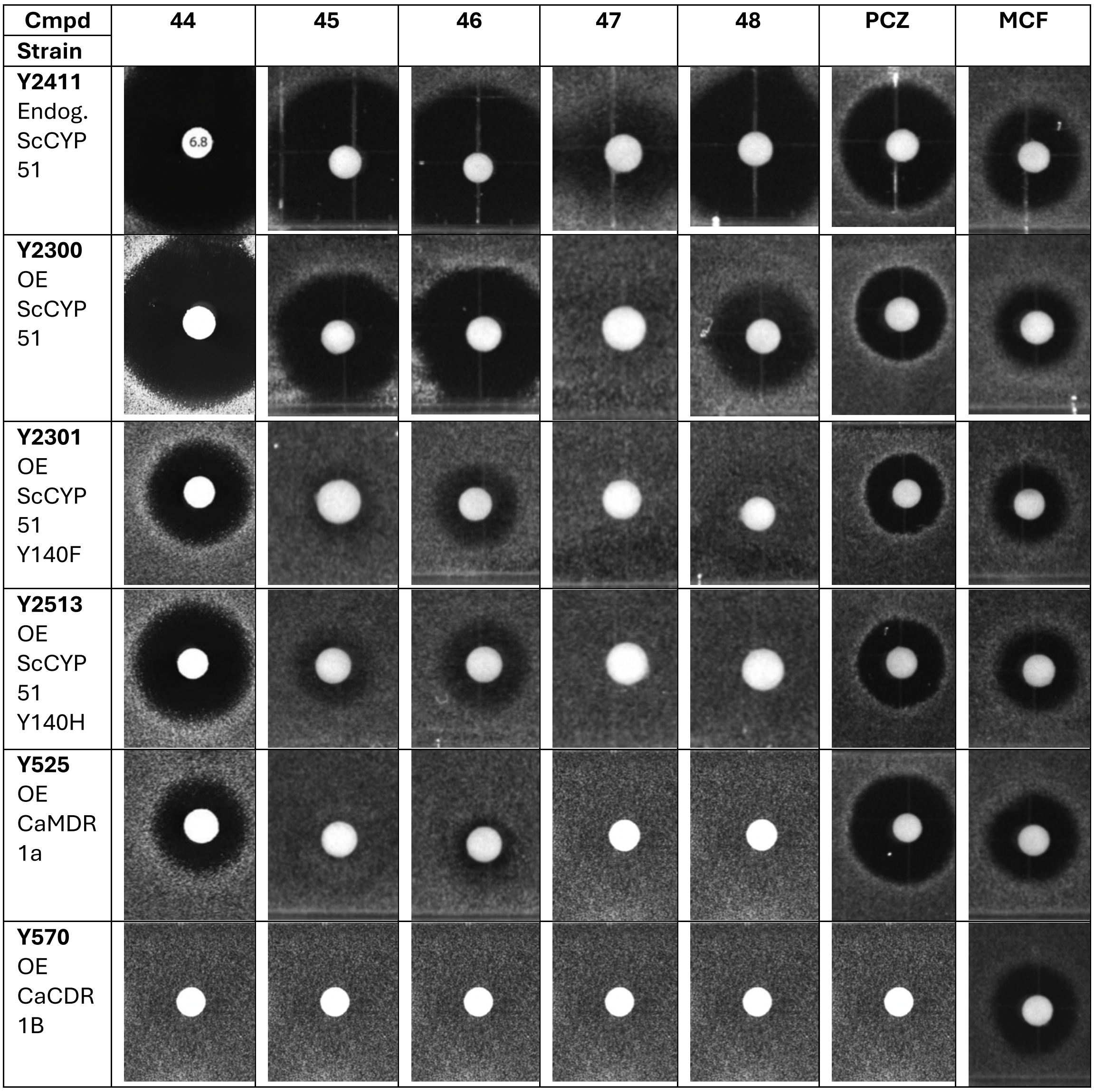

Figure S4. Susceptibilities of yeast constructs and clinical isolates to compounds **44-48**
(Y2411 – azole sensitive, Y2300 ERG11 overexpressed, Y2301 Y140F mutant, Y2513 Y140H mutant, Y525 Ca MDR1a MFS, Y570 CaCDR1B ABC. PCZ posaconazole, MCF micafungin.)

Table S2. Data collection and refinement statistics for ScCYP51 in complex with **22**.

**PDB ID**	8VK6
Wavelength (Å)	0.9537
Resolution range (Å)	44.11 - 1.89 (1.93 - 1.89)
Space group	P 1 2_1 1
Unit cell (Å)	76.69, 65.54, 80.96,
	90, 98.50, 90
Total reflections	121946 (11609)
Unique reflections	61571 (5923)
Multiplicity	2.0 (2.0)
Completeness (%)	96.61 (93.46)
Mean I/sigma(I)	7.0 (0.6)
Wilson B-factor	38.62
R_{merge}	0.02878 (0.8067)
R_{meas}	0.04069 (1.141)
R_{pim}	0.02878 (0.8067)
$CC_{1/2}$	0.999 (0.559)
CC*	1 (0.847)
Reflections used in refinement	61500 (5900)
Reflections used for R-free	2004 (191)
R_{work}	0.1954 (0.3487)
R_{free}	0.2250 (0.3508)
CC(work)	0.951 (0.742)
CC(free)	0.930 (0.615)
Number of non-hydrogen atoms	4532
macromolecules	4301
ligands	94
solvent	137
RMS(bonds)	0.008
RMS(angles)	0.86
Ramachandran favored (%)	97.14
Ramachandran allowed (%)	2.67
Ramachandran outliers (%)	0.19
Rotamer outliers (%)	1.30
Clashscore	2.97

Statistics for the highest-resolution shell are shown in parentheses. This research was undertaken on the MX2 beamline at the Australian Synchrotron.

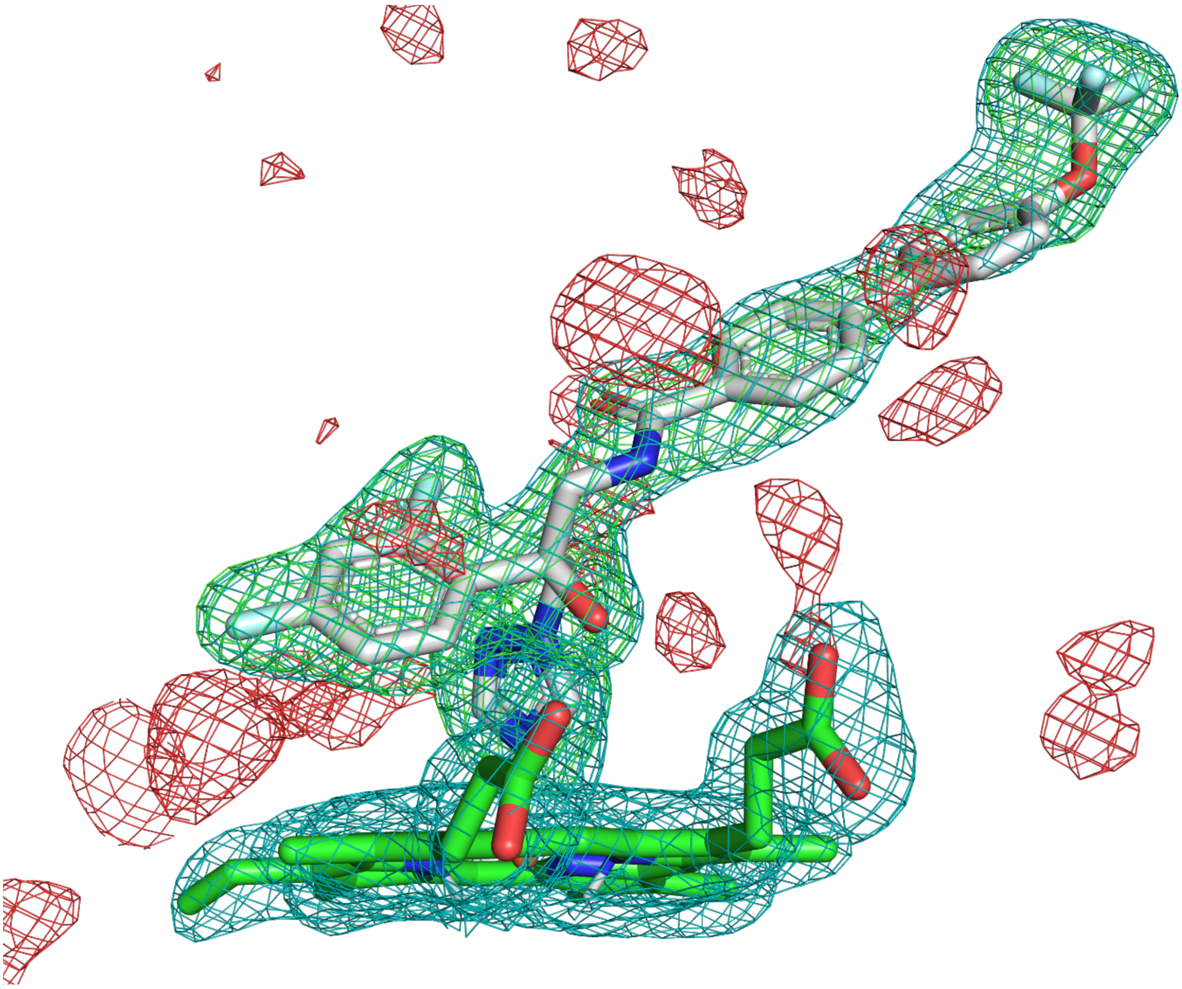

Figure S5: Omit map showing only the *S*-**22** enantiomer (white) complexed with ScCYP51. The 2*Fo* − *Fc* electron density map (blue) is contoured at 1σ, and the *Fo* − *Fc* maps are contoured at 3σ (green) or -3σ (red). Maps (ccp4) were generated by Phenix for visualization in PyMOL. Maps were calculated using Fcalc refined from coordinates without ligand present at the active site.

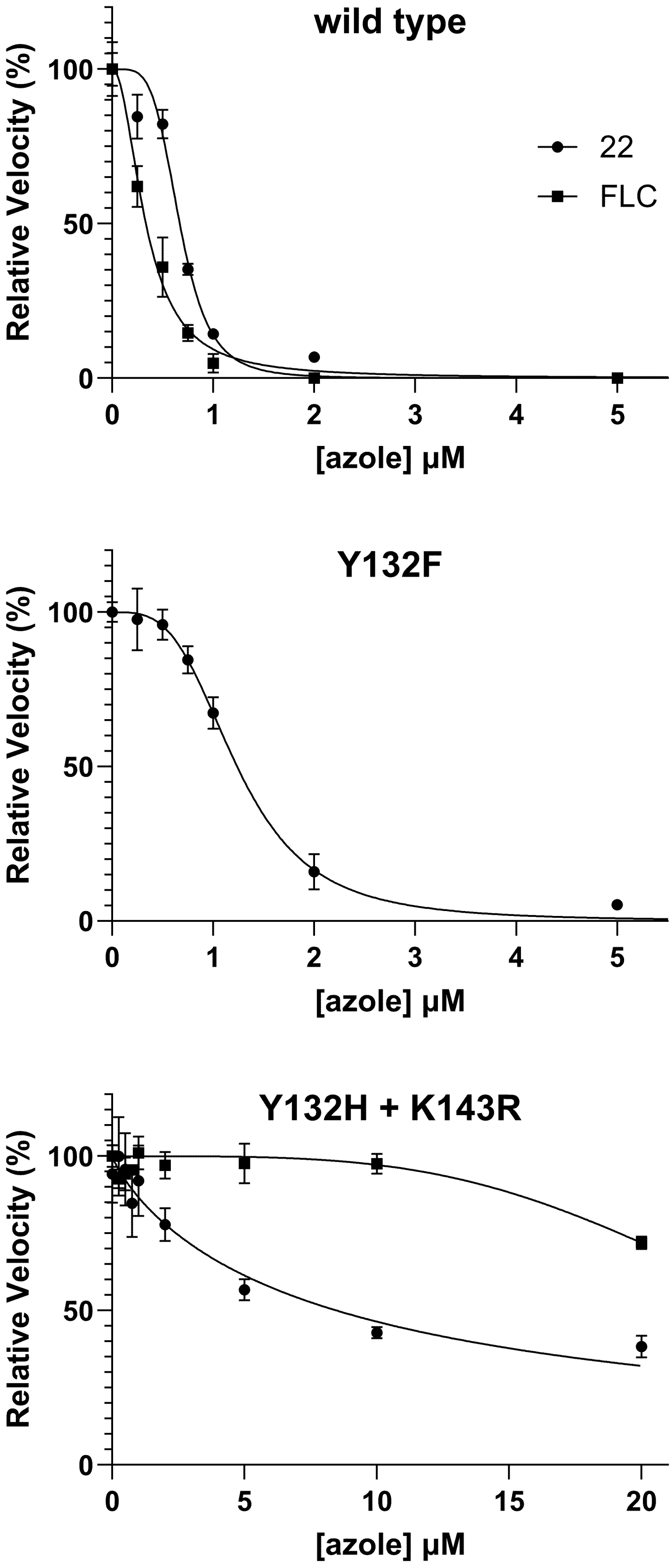

Figure S6. IC_{50} determinations of fluconazole (FLC) and compound **22**. IC_{50}s were determined for wild type CaCYP51, CaCYP51 Y132F and CaCYP51 Y132H + K143R with FLC (squares) and compound **22** (circles). Assays were performed in duplicate. Data are presented as normalised mean points with standard error of the mean error bars.

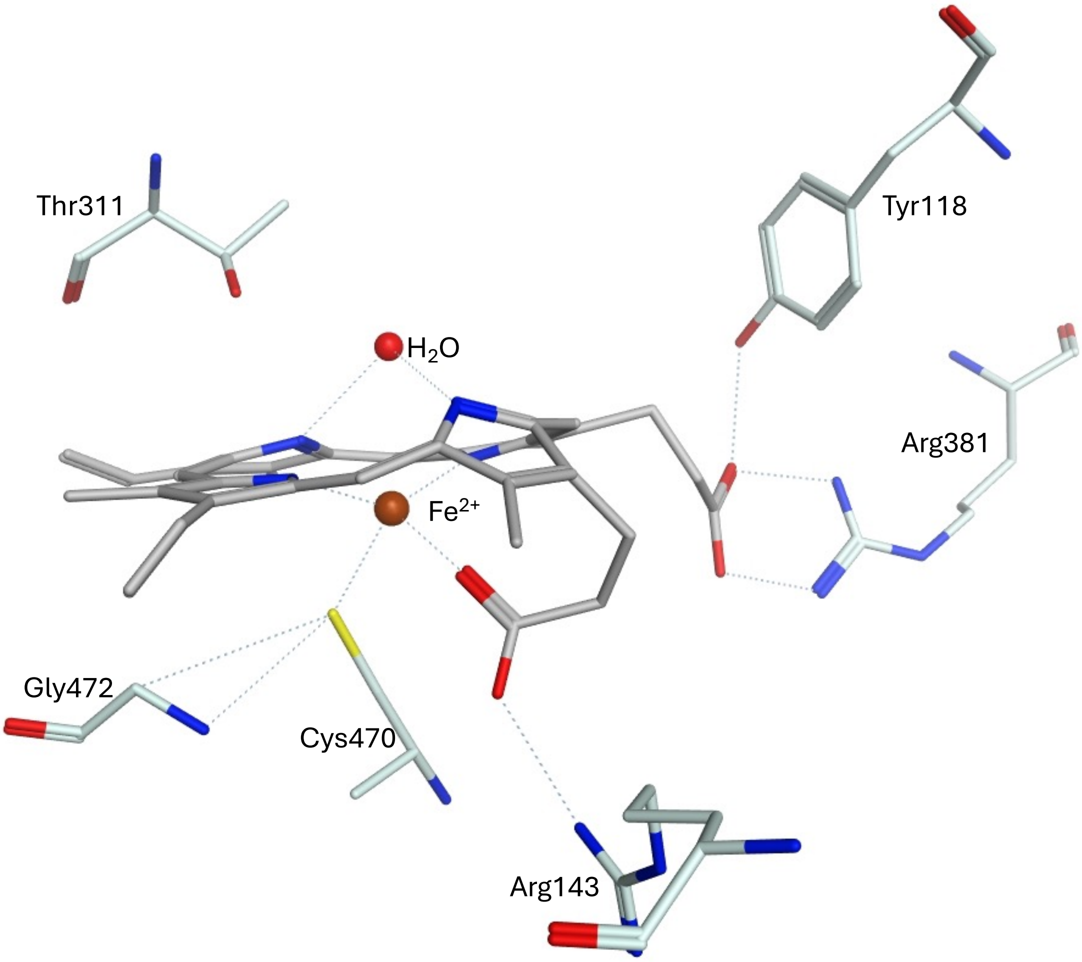

Figure S7. Distortion of the haem after 150 ns molecular dynamics simulation of the CaCYP51 Y132H-K143R protein. The Fe (brown sphere) moves out of plane of the protoporphyrin ring (grey), resulting in the loss of two hydrogen bonds between the Fe and two of the four pyrroles in the protoporphyrin ring.

R-**22**	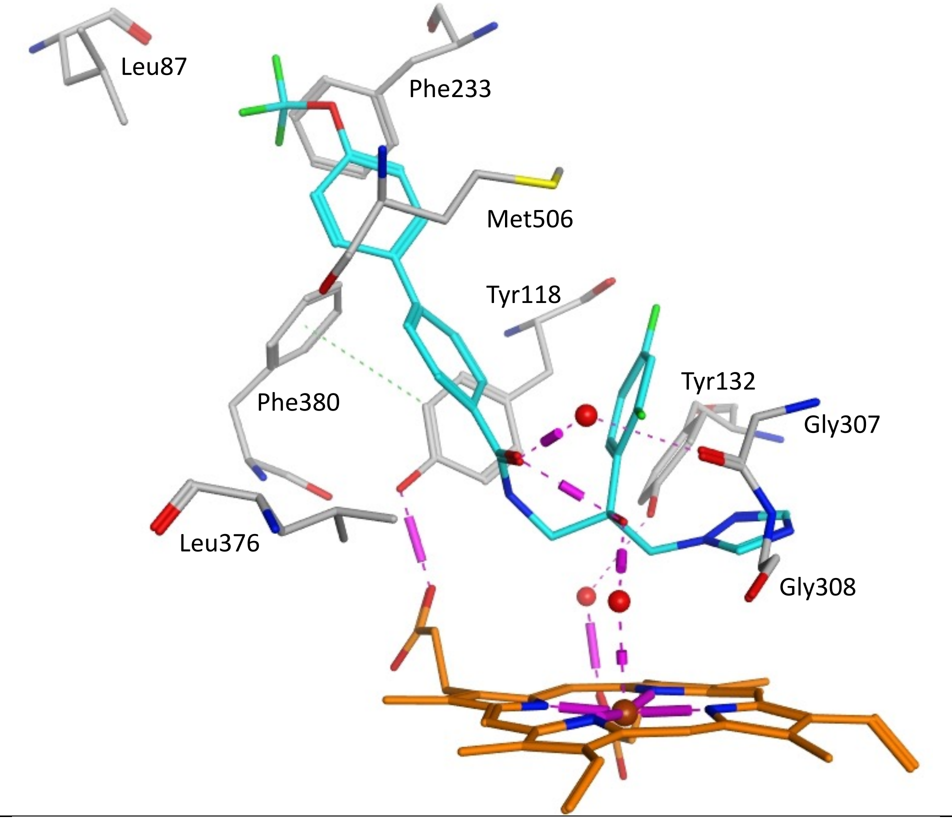
S-**22**	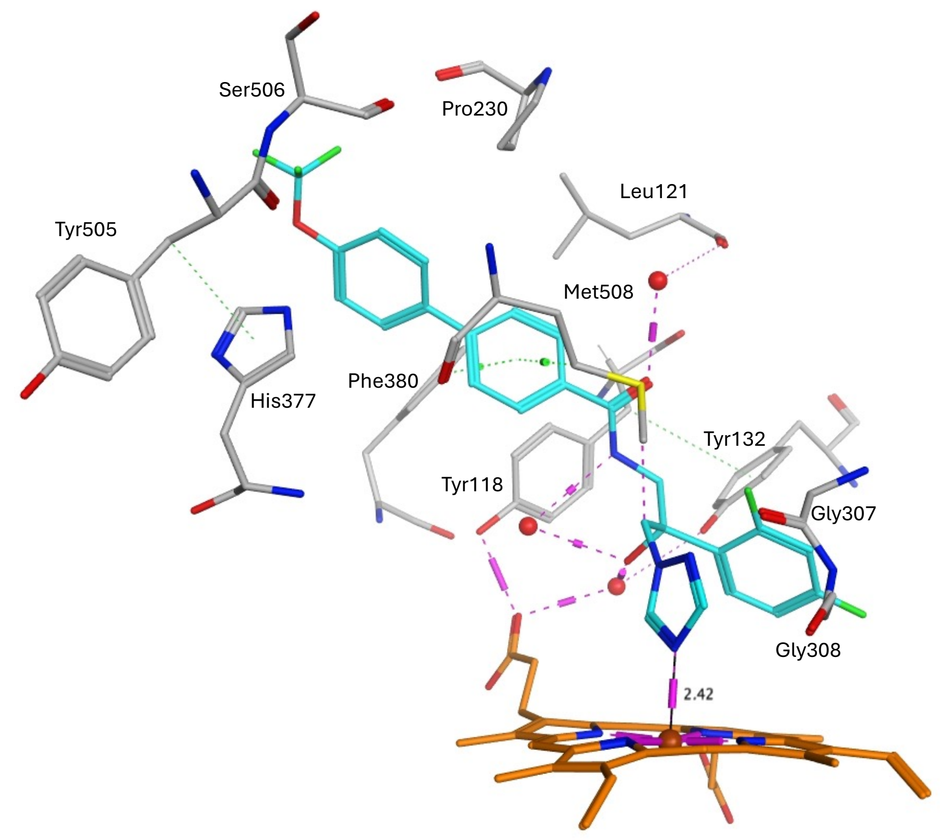

Figure S8. 3D image of *R*-**22** and *S*-**22** in CaCYP51 wild-type [Haem in orange, H_2O shown as red spheres] after 150 ns MD simulation.

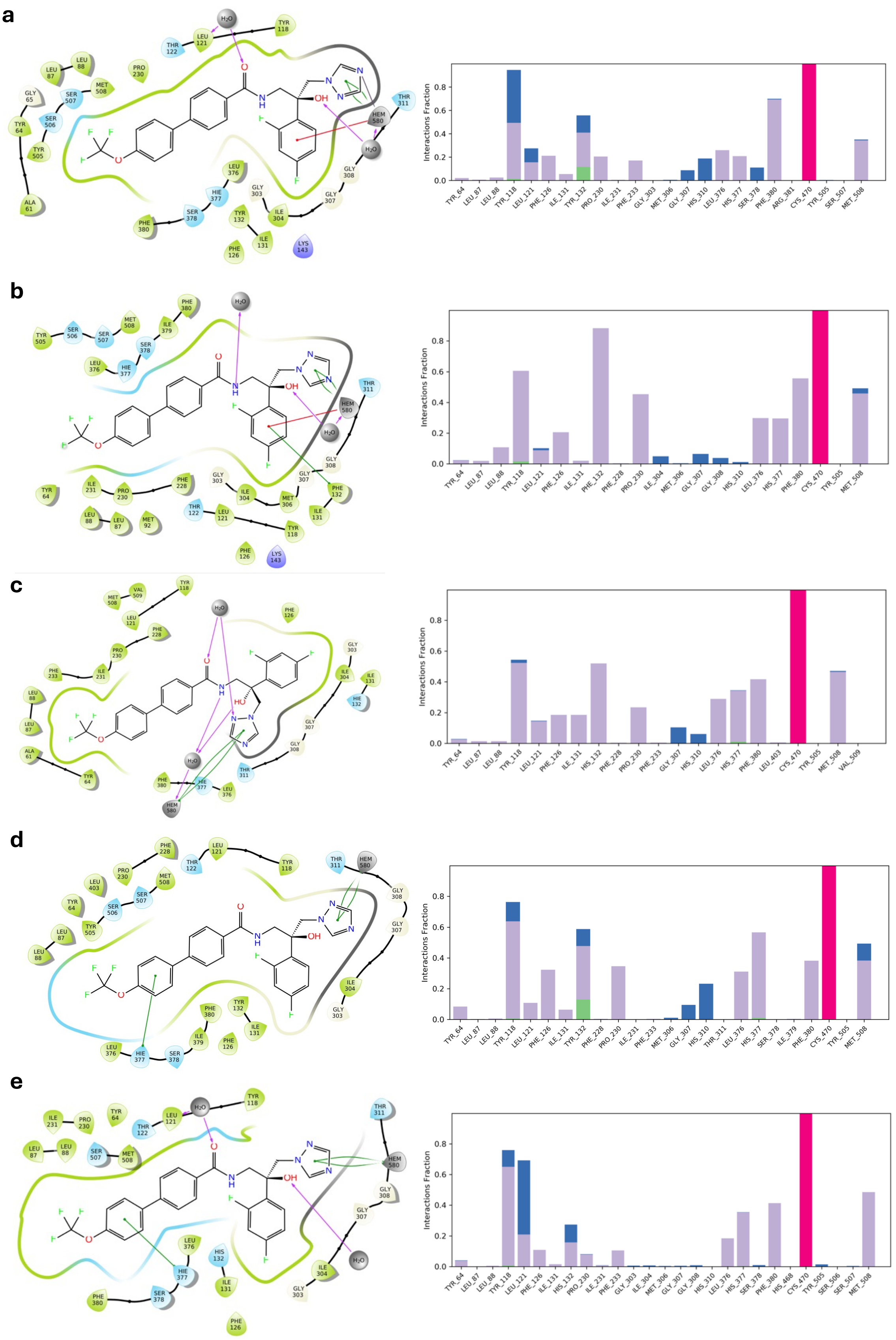

Figure S9. 2D ligand interactions after 150 ns MD simulation and ligand-binding interaction graph showing interactions of *S*-**22** over the 150 ns MD simulation with (a) CaCYP51 WT, single amino acid mutant (b) CaCYP51 Y132F (c) CaCYP51 Y132H (d) CaCYP51 K143R, and double mutant (e) CaCYP51 Y132H + K143R.

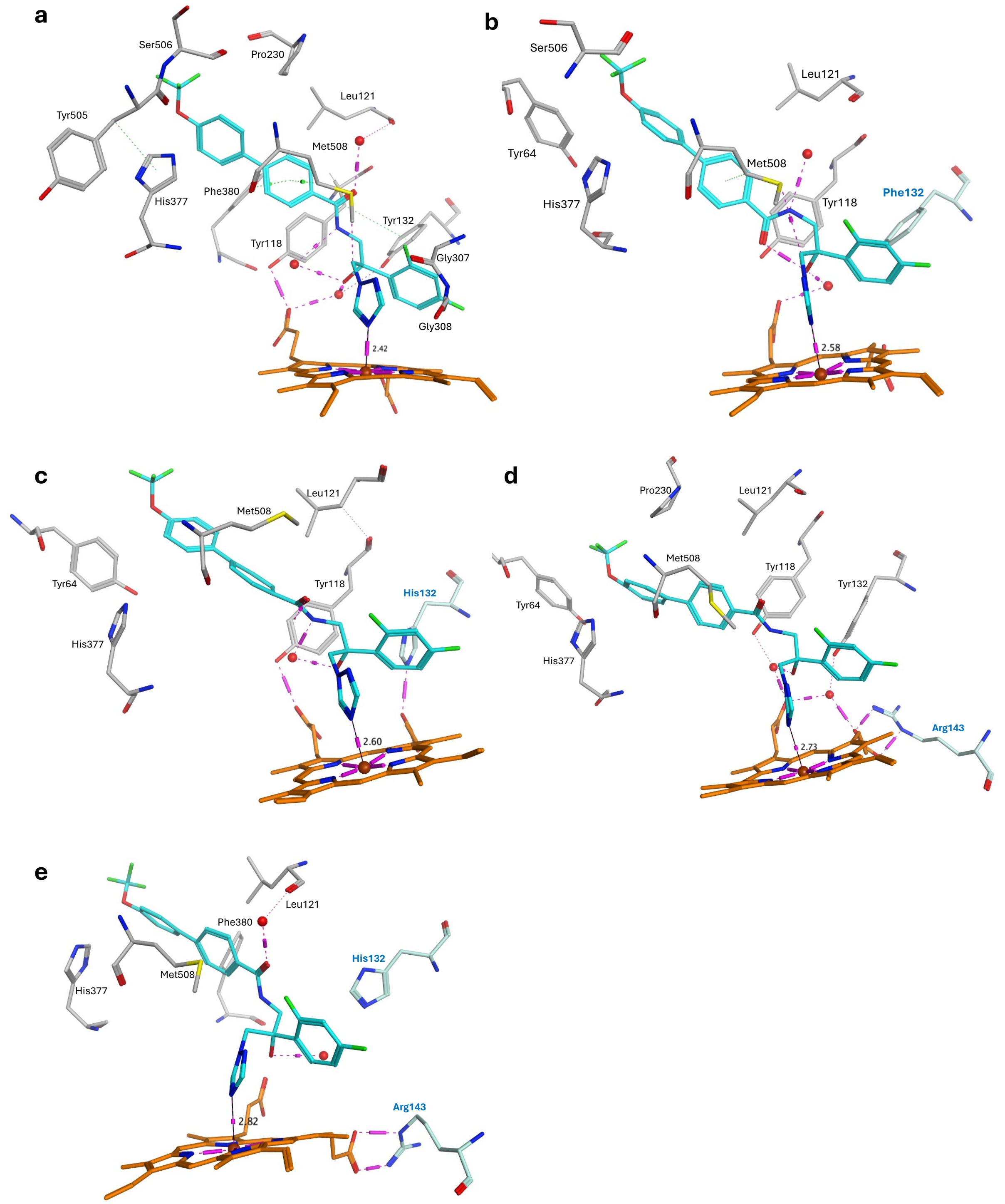

Figure S10. 3D image of interactions of *S*-**22** with (a) CaCYP51 WT, single amino acid mutant (b) CaCYP51 Y132F (c) CaCYP51 Y132H (d) CaCYP51 K143R, and double mutant (e) CaCYP51 Y132H + K143R, after 150 ns MD simulation

METHODS FOR SYNTHESIS OF INTERMEDIATES

1-((2-(2,4-difluorophenyl) oxiran-2-yl) methyl)-1*H*-1,2,4-triazole (2)

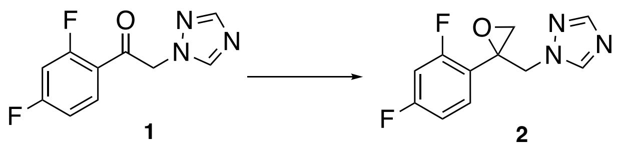

To a solution of 1(2,4-difluorophenyl)-2-(1*H*-1,2,4-triazol-1-yl)ethan-1-one (**1**) (3.3 g, 14.8 mmol) in toluene (165 mL) was added trimethyl sulfoxonium iodide (TMSOI) (6.51 g, 29.6 mmol) and the reaction heated at 60 °C for 6 h. The reaction was diluted with H_2O (50 mL) and EtOAc (100 mL). The aqueous layer was extracted with EtOAc (2 x 50 mL), then the combined organic extracts washed with H_2O (2 x 50 mL), brine (50 mL) and dried ($MgSO_4$) and concentrated under reduced pressure to give the crude product as a dark orange oil. Yield: 3.04 g (88 %); TLC: (epoxide not visible at 254 or 360 nm, reaction monitored by disappearance of **1** on TLC). ^{1}H NMR (DMSO-d_6) δ: 8.39 (s, 1H, triaz), 7.91 (s, 1H, triaz), 7.28 (dt, J = 2.5, 10.8 Hz, 1H, H-3), 7.21 (dd, J = 6.0, 8.6 Hz, 1H, H-6), 7.02 (dt, J = 2.6, 8.6 Hz, 1H, H-5), 4.77 (d, J = 14.9 Hz, 1H, CH_2-triaz), 4.58 (d, J = 14.9 Hz, 1H, CH_2-triaz), 3.10 (d, J = 4.8 Hz, 1H, CH_2-epoxide), 2.96 (d, J = 4.8 Hz, 1H, CH_2-epoxide).

1-Azido-2-(2,4-difluorophenyl)-3-(1*H*-1,2,4-triazol-1-yl) propan-2-ol (3)

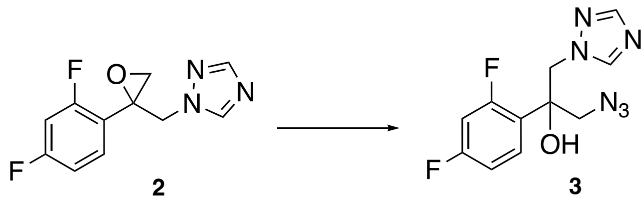

To a solution of crude (**2**) (3.04 g, 12.8 mmol) in dry DMF (35 mL) was added NaN_3 (1.62 g, 25 mmol) followed by NH_4Cl (0.82 g, 15.4 mmol) and the reaction heated at 60 °C for 2 h then overnight at room temperature. Then, the reaction was quenched in an ice bath by addition of $NaHCO_3$ (100 mL). The aqueous layer was back extracted with EtOAc (3 x 50 mL) then the combined organic extracts washed with H_2O (100 mL) and brine (100 mL), dried ($MgSO_4$) and

concentrated under reduced pressure to give a dark orange syrup. The residue was purified by gradient column chromatography (petroleum ether – EtOAc 50:50 v/v) to give the pure product as a dark orange syrup. Yield: 2.59 g (72 %); TLC: petroleum ether – EtOAc 1:2 v/v, R_f 0.5. ^{1}H NMR (DMSO-d_6) δ: 8.31 (s, 1H, triaz), 7.80 (s, 1H, triaz), 7.45 (dd, J = 9.1, 15.9 Hz, 1H, H-6), 7.21 (dt, J = 2.6, 12.0 Hz, 1H, H-5), 7.03 (dt, J = 2.6, 8.5 Hz, 1H, H-3), 6.39 (br. s, 1H, OH), 4.56 (s, 2H, CH_2-triaz), 3.73 (d, J = 13.3 Hz, 1H, CH_2-N_3), 3.63 (d, J = 13.2 Hz, 1H, CH_2-N_3).

1-Amino-2-(2,4-difluorophenyl)-3-(1*H*-1,2,4-triazol-1-yl)propan-2-ol (4)

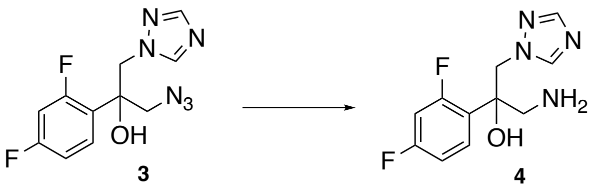

To a solution of 1-azido-2-(2,4-difluorophenyl)propan-2-ol **(3)** (1.2 g , 4.28 mmol) in EtOH (14 mL) was added 10% Pd/C (120 mg). The solution was stirred at room temperature for 5 h under a hydrogen atmosphere (35-48 psi, Paar hydrogenator), then filtered through celite. The filtrate was concentrated, and the residue was washed with Et_2O, then dried at 40 °C in a vacuum oven to give the product as a white solid. Yield: 0.74 g (69 %); mp 98-100 °C; TLC: CH_2Cl_2-MeOH 9:1 v/v, R_f 0.37. ^{1}H NMR (DMSO-d_6) δ: 8.28 (s, 1H, triaz), 7.73 (s, 1H, triaz), 7.38 (dd, J = 9.0, 16.0 Hz, 1H, H-6), 7.16 (dt, J = 2.6, 12.0 Hz, 1H, H-5), 6.95 (dt, J = 2.6, 8.6 Hz, 1H, H-3), 4.53 (s, 2H, CH_2-triaz), 2.92 (s, 2H, CH_2-NH_2).

4'-(Trifluoromethyl)-[1,1'-biphenyl]-4-carboxylic acid (17)

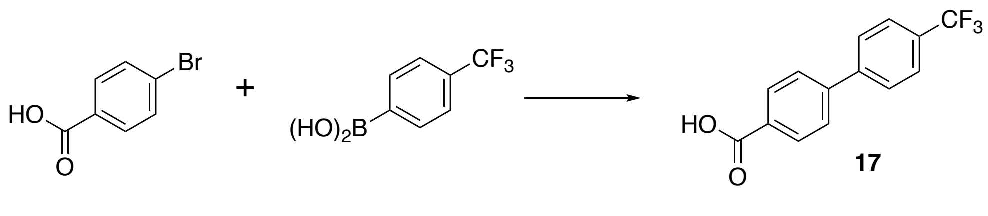

Method: Under nitrogen atmosphere, 4-bromobenzoic acid (0.3g, 1.49 mmol), 4-trifluoromethyl phenylboronic acid (0.42g, 2.23 mmol) and 1 mol % of $Pd(PPh_3)_4$ (0.17 mg)

were dissolved in a solution of dioxane/H_2O (10:1 v/v). Then, K_2CO_3 (0.41 g, 2.93 mmol) was added and the mixture heated under reflux for 6 h. The reaction mixture was cooled to room temperature and the dioxane was evaporated. To the resulting residue, H_2O (20 mL) was added and the solution adjusted to pH 1-3 with 2N aqueous HCl, then extracted with EtOAc (3 x 50 mL) and dried ($MgSO_4$). The organic layer was evaporated under reduced pressure to give a crude light gray semisolid. The residue was purified by gradient column chromatography and the desired product was eluted with 2% MeOH in CH_2Cl_2.as a white solid. Yield: 0.32g (82 %); mp 240-243°; TLC: CH_2Cl_2-MeOH 95:5 v/v, R_f 0.42. ^{1}H NMR (DMSO-d_6) δ: 13.07 (s, 1H, OH), 8.06 (d, J = 8.7 Hz, 2H, Ar), 7.87 (dd, J = 5.2, 8.7 Hz, 4H, Ar), 7.71 (d, J = 8.7 Hz, 2H, Ar). ^{19}F NMR (DMSO-d_6): δ -60.92 (CF_3).

Using this procedure, the following compounds were prepared:

4'-(Trifluoromethoxy)-[1,1'-biphenyl]-4-carboxylic acid (18)

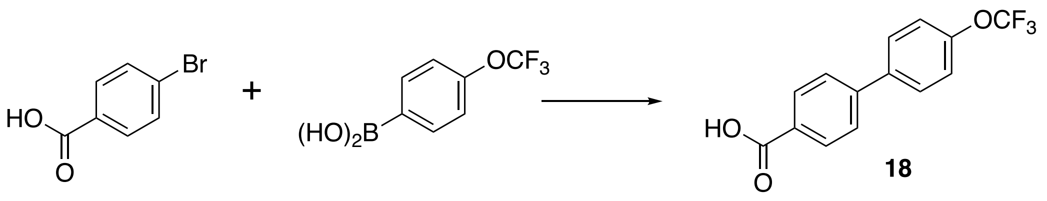

18

Prepared from 4-bromobenzoic acid (0.3 g, 1.49 mmol) and 4-trifluoromethoxy phenylboronic acid (0.46 g, 2.23 mmol). Yield: 0.36g (85 %) as a white solid; mp 251-253°; TLC: CH_2Cl_2-MeOH 95:5 v/v, R_f 0.47. ^{1}H NMR (DMSO-d_6) δ: 13.03 (s, 1H, OH), 8.03 (d, J = 8.7 Hz, 2H, Ar), 7.86 (d, J = 8.9 Hz, 2H, Ar), 7.82 (d, J = 8.7 Hz, 2H, Ar), 7.49 (d, J = 8.0 Hz, 2H, Ar). ^{19}F NMR (DMSO-d_6): δ -56.74 (CF_3).

4'-(Trifluoromethyl)-[1,1'-biphenyl]-3-carboxylic acid (19)

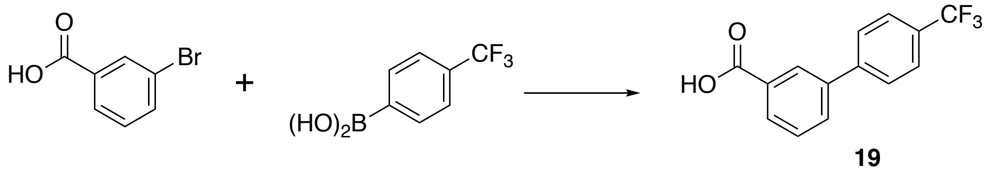

19

Prepared from 3-bromobenzoic acid (0.3 g, 1.49 mmol) and 4-trifluoromethyl phenylboronic acid (0.42 g, 2.23 mmol). The residue was purified by gradient column chromatography and the desired product was eluted with 2.5 % MeOH in CH_2Cl_2 as a white solid. Yield: 0.35 g (90 %); mp 198-200°; TLC: CH_2Cl_2-MeOH 95:5 v/v, R_f 0.45. ^{1}H NMR (DMSO-d_6) δ: 13.18 (s, 1H, OH), 8.24 (s, 1H, Ar), 8.00 (m, 2H, Ar), 7.94 (d, J = 8.0 Hz, 2H, Ar), 7.84 (d, J = 8.1 Hz, 2H, Ar), 7.65 (t, J = 7.7 Hz, 1H, Ar). ^{19}F NMR (DMSO-d_6): δ -60.96 (CF_3).

4'-(Trifluoromethoxy)-[1,1'-biphenyl]-3-carboxylic acid (20)

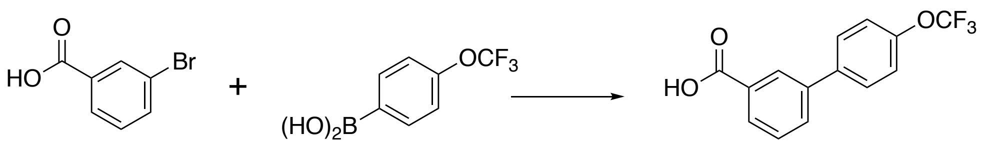

20

Prepared from 3-bromobenzoic acid (0.3 g, 1.49 mmol) and 4-trifluoromethoxy phenylboronic acid (0.46 g, 2.23 mmol). The residue was purified by gradient column chromatography and the desired product was eluted with 2.5 % MeOH in CH_2Cl_2 as a light brown solid. Yield: 0.35 g (83 %); mp 158-160°; TLC: CH_2Cl_2-MeOH 95:5 v/v, R_f 0.35. ^{1}H NMR (DMSO-d_6) δ: 13.13 (s, 1H, OH), 8.19 (s, 1H, Ar), 7.95 (dd, J = 7.7, 15.9 Hz, 2H, Ar), 7.84 (d, J = 8.9 Hz, 2H, Ar), 7.62 (t, J = 7.7 Hz, 1H, Ar), 7.47 (d, J = 7.9 Hz, 2H, Ar). ^{19}F NMR (DMSO-d_6): δ -56.74 (CF_3). ^{13}C NMR (DMSO-d_6): δ 167.55 (C, C=O), 148.60 (C, Ar), 139.53 (C, Ar), 139.02 (C, Ar), 132.06 (C, Ar), 131.69 (CH, Ar), 129.89 (CH, Ar), 129.24 (2 x CH, Ar), 129.11 (CH), 127.89 (CH), 122.00 (2 x CH, Ar), 120.56 (q, J_{CF_3} = 256.24 Hz, C, CF_3). HPLC: 100 %, RT = 4.81 min. HRMS (ESI) m/z: theoretical mass: 281.0426 $[M-H]^-$, observed mass: 281.0431 $[M-H]^-$.

4-Amino-*N*-(2-(2,4-difluorophenyl)-2-hydroxy-3-(1*H*-1,2,4-triazol-1-yl)propyl) benzamide (25)

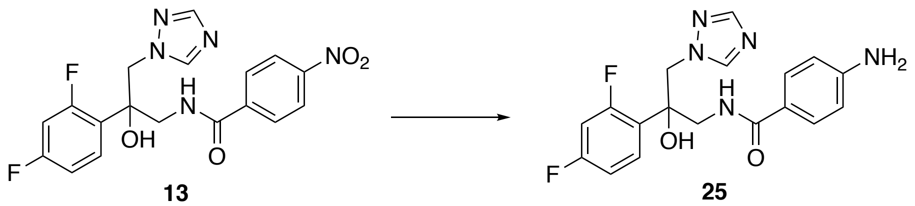

13 → 25

To a solution of *N*-(2-(2,4-difluorophenyl)-2-hydroxy-3-(1*H*-1,2,4-triazol-1-yl)propyl)-4-nitrobenzamide (**13**) (0.48 g, 1.19 mmol) in dry MeOH (15 mL) was added 10 % Pd/C (44 mg). Then, the reaction atmosphere was filled with H_2 using H_2 balloons, and the mixture was stirred at room temperature for 3 h. The suspension was filtered through a pad of celite and the solvent evaporated under reduced pressure to give the crude product, which was purified by column chromatography and the desired compound eluted at 2.5% MeOH in CH_2Cl_2. Yield: 0.44 g (100 %) as an off-white semisolid; TLC: CH_2Cl_2-MeOH 95:5 v/v, R_f 0.35. ^{1}H NMR (DMSO-*d*$_6$) δ: 8.33 (s, 1H, triaz), 8.20 (t, *J* = 6.0 Hz, 1H, NH), 7.73 (s, 1H, triaz), 7.50 (d, *J* = 8.7 Hz, 2H, Ar), 7.40 (dd, *J* = 9.0, 15.9 Hz, 1H, Ar), 7.17 (m, 1H, Ar), 6.93 (t, *J* = 8.3 Hz, 1H, Ar), 6.67 (s, 1H, OH, ex), 5.69 (s, 2H, NH_2), 4.64 (d, *J* = 14.4 Hz, 1H, CH_2-triaz), 4.53 (d, *J* = 14.3 Hz, 1H, CH_2-triaz), 3.73 (dd, *J* = 5.5, 14.2 Hz, 1H, CH_2-NH), 3.70 (dd, *J* = 6.3, 14.3 Hz, 1H, CH_2-NH). ^{13}C NMR (DMSO-*d*$_6$): δ 168.90 (C, C=O), 162.23 (dd, $^3J_{CF}$ = 12.5 Hz, $^1J_{CF}$ =245.4 Hz, C, C2-Ar), 159.46 (dd, $^3J_{CF}$ = 12.5 Hz, $^1J_{CF}$ =247.5 Hz, C, C4-Ar), 152.58 (C, C-NH_2), 151.02 (CH, triaz), 145.44 (CH, triaz), 140.22 (C, Ar), 130.44 (dd, $^3J_{CF}$ = 6.2 Hz, $^3J_{CF}$ =9.6 Hz, CH, C6-Ar), 129.50 (2 X CH, Ar), 125.48 (dd, $^4J_{CF}$ = 3.5 Hz, $^2J_{CF}$ =13.0 Hz, C, C1-Ar), 120.17 (C, Ar), 112.89 (2 x CH, Ar), 111.19 (d, $^2J_{CF}$ = 17.7 Hz, CH, C5-Ar), 104.36 (t, $^2J_{CF}$ = 27.8 Hz, CH, C3-Ar), 75.85 (C-OH), 55.77 (CH_2-triaz), 47.47 (CH_2-NH_2). HPLC: 100 %, RT = 4.04 min. HRMS (ESI) m/z: theoretical mass: 374.1428 $[M+H]^+$, observed mass: 374.1430 $[M+H]^+$.

Methyl 2-(2,4-difluorophenyl) acetate (28)

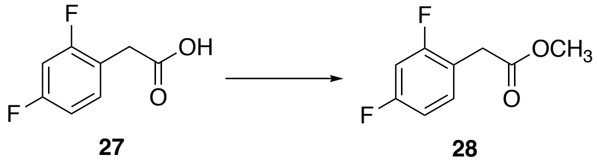

To a colourless solution of 2-(2,4-difluorophenyl) acetic acid (**27**) (1.5 g, 8.71 mmol) in dry MeOH (15 mL) was added $SOCl_2$ (1.26 mL, 17.42 mmol) dropwise while cooling in an ice-bath. The mixture was heated to 60 °C for 3 h. After evaporation, the residue was dissolved in EtOAc (70 mL), washed with aqueous $NaHCO_3$ (3 x 35 mL), H_2O (3 x 35 mL) and brine (35 mL), dried ($MgSO_4$), and concentrated under vacuum to give the product that was used in the next step without further purification. Yield: 1.48 g (91 %) as yellow oil; TLC: petroleum ether-EtOAc 3:1 v/v, R_f 0.65. ^{1}H NMR (DMSO-d_6) δ: 8.33 (s, 1H, triaz), 7.42 (q, J = 8.7 Hz, 1H, Ar), 7.23 (t, J = 10.3 Hz, 1H, Ar), 7.06 (t, J = 7.5 Hz, 1H, Ar), 3.73 (s, 2H, CH_2), 3.63 (s, 3H, CH_3). ^{19}F NMR (DMSO-d_6): δ -106.76 (*para*-F-Ar), -112.09 (*ortho*-F-Ar).

Methyl 2-(2,4-difluorophenyl)-3-hydroxypropanoate (29)

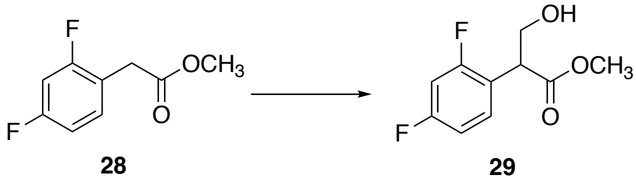

To a stirred solution of methyl 2-(2,4-difluorophenyl) acetate (**28**) (1.39 g, 7.46 mmol) in dry DMSO (20 mL) was added sodium methoxide (0.02 g, 0.37 mmol) at 0 °C. Paraformaldehyde (0.23 g, 7.84 mmol) was then added and the reaction mixture stirred at room temperature for 4 h. The reaction mixture was diluted with EtOAc (70 mL), washed with water (3 x 35 mL), brine (35 mL), dried ($MgSO_4$) and evaporated *in vacuo* to afford the crude product. The crude material was purified by gradient column chromatography eluting with petroleum ether-EtOAc 60:40 v/v. Yield: 1.26 g (78 %) as a colourless oil; TLC: petroleum ether-EtOAc 1:1 v/v, R_f 0.50. ^{1}H NMR (DMSO-d_6) δ: 7.44 (q, J = 8.7 Hz, 1H, Ar), 7.23 (t, J = 10.6 Hz, 1H, Ar), 7.08

(t, J = 8.5 Hz, 1H, Ar), 5.06 (t, J = 5.5 Hz, 1H, OH-ex), 3.98 (dd, J = 7.4, 13.7 Hz, 1H, CH_2), 3.93 (dd, J = 4.7, 10.4 Hz, 1H, CH_2), 3.68 (m, 1H, CH), 3.62 (s, 3H, CH_3). ^{13}C NMR (DMSO-d_6): δ 172.08 (C, C=O), 162.23 (dd, $^3J_{CF}$ = 12.6 Hz, $^1J_{CF}$ = 160.0 Hz, C, C2-Ar), 160.27 (dd, $^3J_{CF}$ = 12.2 Hz, $^1J_{CF}$ = 161.8 Hz, C, C4-Ar), 131.32 (dd, $^3J_{CF}$ = 5.5 Hz, $^3J_{CF}$ = 9.6 Hz, CH, C6-Ar), 120. 61 (dd, $^4J_{CF}$ = 4.1 Hz, $^2J_{CF}$ = 15.3 Hz, CH, C1-Ar), 112.00 (dd, $^4J_{CF}$ = 3.5 Hz, $^2J_{CF}$ = 21.0 Hz, CH, C5-Ar), 104.27 (t, $^2J_{CF}$ = 26.7 Hz, CH, C3-Ar), 62.29 ($\underline{C}H_2$), 52.41($\underline{C}H_3$), 46.51 ($\underline{C}$H). HPLC: 100 %, RT = 4.23 min. HRMS (ESI) m/z: theoretical mass: 239.0495 $[M+Na]^+$, observed mass: 239.0499 $[M+Na]^+$.

Methyl 2-(2,4-difluorophenyl)-3-((methylsulfonyl)oxy) propanoate (30)

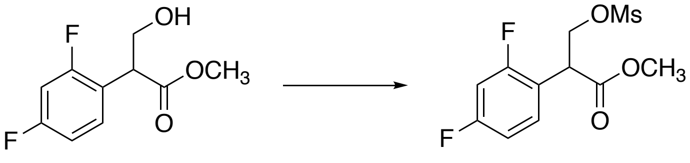

To an ice-cooled suspension of methyl 2-(2,4-difluorophenyl)-3-hydroxypropanoate (**29**) (2.94 g, 13.59 mmoL) in dry CH_2Cl_2 (25 mL), was added Et_3N (2.84 mL, 20.39 mmoL) followed by methane sulfonyl chloride (2.63 mL, 33.99 mmoL) dropwise. The reaction was stirred at 0 °C for 1 h then at room temperature overnight. After evaporation, the reaction was diluted with EtOAc (100 mL), washed with H_2O (2 x 50 mL), dried ($MgSO_4$) and evaporated under vacuum. The product was purified by gradient column chromatography and the desired product was eluted with 40 % EtOAc in petroleum ether. Yield: 3.39 g (84 %) as a colourless oil; TLC: petroleum ether-EtOAc 1:1 v/v, R_f 0.55. ^{1}H NMR (DMSO-d_6) δ: 7.48 (q, J = 8.7 Hz, 1H, Ar), 7.31 (t, J = 8.1 Hz, 1H, Ar), 7.13 (t, J = 8.5 Hz, 1H, Ar), 4.70 (dd, J = 6.8, 10.1 Hz, 1H, CH_2), 4.50 (dd, J = 7.0, 10.1 Hz, 1H, CH_2), 4.41 (t, J = 6.9 Hz, 1H, CH), 3.66 (s, 3H, OCH_3), 3.16 (s, 3H, CH_3). ^{13}C NMR (DMSO-d_6): δ 170.34 (C, C=O), 162.57 (dd, $^3J_{CF}$ = 12.6 Hz, $^1J_{CF}$ = 206.7 Hz, C, C2-Ar), 160.60 (dd, $^3J_{CF}$ = 12.6 Hz, $^1J_{CF}$ = 207.9 Hz, C, C4-Ar), 131.94 (dd, $^3J_{CF}$ = 5.3 Hz, $^3J_{CF}$ = 9.63 Hz, CH, C6-Ar), 118.88 (dd, $^4J_{CF}$ = 3.7 Hz, $^2J_{CF}$ = 15.1 Hz, CH, C1-Ar), 112.40

(dd, $^4J_{CF}$ = 3.5 Hz, $^2J_{CF}$ = 21.2 Hz, CH, C5-Ar), 104.71 (t, $^2J_{CF}$ = 26.2 Hz, CH, C3-Ar), 69.10 ($\underline{C}H_2$), 52.96 (O$\underline{C}H_3$), 43.56 ($\underline{C}$H), 37.03 ($\underline{C}H_3$). HPLC: 92.6 %, RT = 4.29 min. HRMS (ESI) m/z: theoretical mass: 317.0271 $[M+Na]^+$, observed mass: 317.0269 $[M+Na]^+$.

Methyl 2-(2,4-difluorophenyl)-3-(1*H*-1,2,4-triazol-1-yl) propanoate (31)

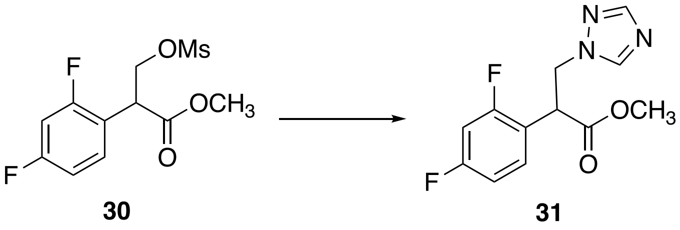

To a stirred solution of triazole (0.17 g, 2.54 mmoL) in dry CH_3CN (2 mL) was added K_2CO_3 (0.35 g, 2.54 mmoL), and the mixture was heated for 1 h at 45 °C. After cooling to room temperature, methyl 2-(2,4-difluorophenyl)-3-((methylsulfonyl)oxy) propanoate (**30**) (0.5 g, 1.69 mmoL) was added and the reaction was heated at 70 °C for 4 h then stirred at room temperature overnight. The solvent was evaporated under vacuum and the residue was extracted with EtOAc (50 mL), washed with brine (3 x 25 mL) and H_2O (3 x 25 mL). The organic layer was dried ($MgSO_4$) and evaporated under vacuum to give the crude product, which was purified by gradient column chromatography and the desired product was eluted with 2 % MeOH in CH_2Cl_2. Yield: 0.38 g (85 %) as a yellow oil; TLC: CH_2Cl_2-MeOH 95:5 v/v, R_f 0.52. ^{1}H NMR (DMSO-d_6) δ: 8.35 (s, 1H, triaz), 7.90 (s, 1H, triaz), 7.33 (q, J = 8.7 Hz, 1H, Ar), 7.23 (t, J = 8.1Hz, 1H, Ar), 7.05 (t, J = 6.0 Hz, 1H, Ar), 4.84 (dd, J = 6.8, 13.7 Hz, 1H, CH_2), 4.62 (dd, J = 8.4, 13.7 Hz, 1H, CH_2), 4.53 (t, J = 6.8 Hz, 1H, CH), 3.62 (s, 3H, OCH_3). ^{13}C NMR (DMSO-d_6) δ:170.93 (C, C=O), 170.93 (C, C=O), 162.47 (dd, $^3J_{CF}$ = 12.1 Hz, $^1J_{CF}$ = 198.9 Hz, C, C2-Ar), 160.50 (dd, $^3J_{CF}$ = 12.2 Hz, $^1J_{CF}$ = 200.1 Hz, C, C4-Ar), 152.01 (CH, triaz), 145.07 (CH, triaz), 131.77 (dd, $^3J_{CF}$ = 5.4 Hz, $^3J_{CF}$ = 9.57 Hz, CH, C6-Ar), 119.48 (dd, $^4J_{CF}$ = 3.7 Hz, $^2J_{CF}$ = 15.0 Hz, CH, C1-Ar), 112.28 (dd, $^4J_{CF}$ = 3.6 Hz, $^2J_{CF}$ = 21.1 Hz, CH, C5-Ar), 104.48 (t, $^2J_{CF}$ = 26.2 Hz, CH, C3-Ar), 52.91 ($\underline{C}H_3$), 49.47 ($\underline{C}H_2$), 44.37 ($\underline{C}$H). HPLC:

100 %, RT = 4.20 min. HRMS (ESI) m/z: theoretical mass: 268.0897 $[M+H]^+$, observed mass: 268.0903 $[M+H]^+$.

2-(2,4-Difluorophenyl)-3-(1*H*-1,2,4-triazol-1-yl) propanoic acid (32)

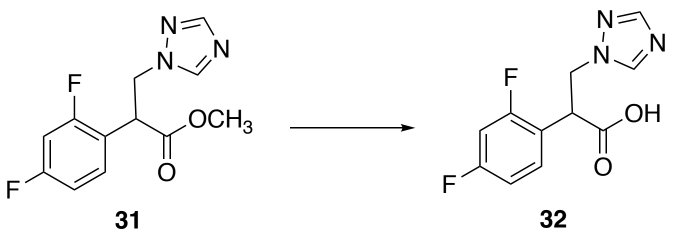

To a stirred solution of methyl 2-(2,4-difluorophenyl)-3-(1*H*-1,2,4-triazol-1-yl) propanoate (**31**) (0.7 g, 2.65 mmoL) in dry THF (8 mL) was added a solution of LiOH • H_2O (0.28 g, 6.62 mmoL) in H_2O (2.4 mL) dropwise at 0 °C and the resulting mixture stirred at room temperature for 1 h. The reaction mixture was acidified (pH 3) with 2N aqueous HCl and extracted with EtOAc (3 x 50 mL). The combined organic layers were washed with brine (3 x 25 mL), water (25 mL), dried ($MgSO_4$) and evaporated under vacuum to give a white powder which was washed with Et_2O. Yield: 0.49 g (73 %) as a white solid; mp 193-195°; TLC: CH_2Cl_2-MeOH 95:5 v/v, R_f 0.12. ^{1}H NMR (DMSO-d_6) δ: 13.02 (brs, 1H, OH), 8.35 (s, 1H, triaz), 7.90 (s, 1H, triaz), 7.34 (q, J = 8.6Hz, 1H, Ar), 7.22 (t, J = 10.5 Hz, 1H, Ar), 7.04 (t, J = 8.5 Hz, 1H, Ar), 4.81 (dd, J = 6.8, 13.8 Hz, 1H, CH_2), 4.55 (dd, J = 8.5, 13.7 Hz, 1H, CH_2), 4.42 (t, J = 8.4 Hz, 1H, CH). ^{13}C NMR (DMSO-d$_6$) δ: 171.89 (C, C=O), 162.42 (dd, $^3J_{CF}$ = 12.2 Hz, $^1J_{CF}$ = 172.8 Hz, C, C2-Ar), 160.45 (dd, $^3J_{CF}$ = 12.6 Hz, $^1J_{CF}$ = 174.5 Hz, C, C4-Ar), 151.93 (CH, triaz), 144.99 (CH, triaz), 131.64 (dd, $^3J_{CF}$ = 5.5 Hz, $^3J_{CF}$ = 9.6 Hz, CH, C6-Ar), 120.15 (dd, $^4J_{CF}$ = 3.7 Hz, $^2J_{CF}$ =15.1 Hz, CH, C1-Ar), 112.14 (dd, $^4J_{CF}$ = 3.5 Hz, $^2J_{CF}$ = 21.4 Hz, CH, C5-Ar), 104.49 (t, $^2J_{CF}$ = 26.1 Hz, CH, C3-Ar), 49.66 ($\underline{C}H_2$), 44.63 ($\underline{C}$H). HPLC: 100 %, RT = 3.97 min. HRMS (ESI) m/z: theoretical mass: 254.0741 $[M+H]^+$, observed mass: 254.0740 $[M+H]^+$.

(4'-(Trifluoromethoxy)-[1,1'-biphenyl]-4-yl)methanamine (36)

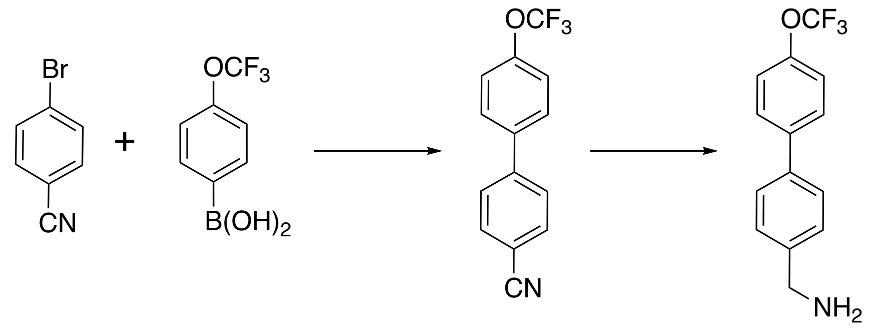

36

Under nitrogen atmosphere, 4-bromobenzonitrile (0.5g, 2.74 mmol), (4-(trifluoromethoxy)phenyl)boronic acid (0.67g, 3.29 mmol) and 1 mol % of $Pd(PPh_3)_4$ (31 mg) were dissolved in a mixture solution of dioxane/H_2O (10:1 v/v). Then, K_2CO_3 (0.75g, 5.48 mmol) was added and the mixture heated under reflux for 6 h. The reaction mixture was cooled to room temperature and the dioxane was evaporated. After that, H_2O (30 mL) was added and the solution was adjusted to pH 1-3 with 2M aqueous HCl, then extracted with EtOAc (3 x 50 mL). The combined organic layers were dried ($MgSO_4$) and concentrated under reduced pressure. The crude product was purified by gradient column chromatography and the desired compound eluted with 20 % EtOAc in petroleum ether to give the intermediate 4'-(trifluoromethoxy)-[1,1'-biphenyl]-4-carbonitrile as a yellow solid. Yield: 0.67 g (93%); mp 53-54 °C (lit. m.p. 50-52°C)[16]; TLC: petroleum ether-EtOAc 3:1 v/v, R_f 0.75. ^{1}H NMR (DMSO-d_6) δ: 7.93 (d, J = 8.7 Hz, 2H, Ar), 7.90 (d, J = 9.2 Hz, 2H, Ar), 7.89 (d, J = 9.0 Hz, 2H, Ar), 7.51 (d, J = 8.0 Hz, 2H, Ar). $LiAlH_4$ in 1M THF (6.83 mL, 6.83 mmol) was added to a solution of 4'-(trifluoromethoxy)-[1,1'-biphenyl]-4-carbonitrile (0.6 g, 2.27 mmol) in anhydrous THF (30 mL) while stirring at -40 °C for 30 min. The reaction mixture was left at 0 °C for 5 h, then H_2O (1 mL) was added to decompose any remaining $LiAlH_4$. Then, 1M aqueous NaOH (0.25 mL) was added and the mixture stirred for 30 min at room temperature. The reaction mixture was filtered through a pad of celite, washed with EtOAc and the filtrate was

evaporated *in vacuo*. H_2O (25 mL) was added to the concentrated mixture which was extracted with EtOAc (3 x 50 mL), dried ($MgSO_4$) and evaporated under reduced pressure. The crude white solid of (4'-(trifluoromethoxy)-[1,1'-biphenyl]-4-yl)methanamine (**36**) was used in the next step without further purification. Yield: 0.50 g (83% crude yield); mp 142-144 °C; TLC: CH_2Cl_2-MeOH 9.5:0.5 v/v 95:5 v/v, R_f 0.17. ^{1}H NMR (DMSO-*d$_6$*) δ: 8.60 (s, 2H, NH_2), 7.91 (d, *J* = 8.5 Hz, 2H, Ar), 7.85 (d, *J* = 9.0 Hz, 2H, Ar), 7.78 (d, *J* = 8.9 Hz, 2H, Ar), 7.67 (d, *J* = 8.4 Hz, 2H, Ar), 4.86 (s, 2H, C$\underline{H_2}$).

2-(2,4-Difluorophenyl)-*N*-(4-nitrobenzyl)-3-(1*H*-1,2,4-triazol-1-yl)propenamide (42)

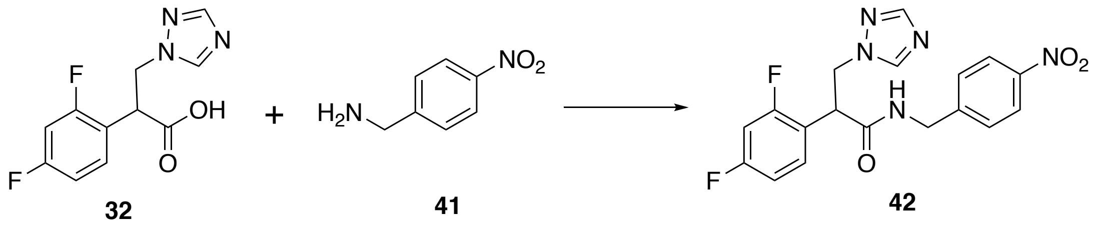

Tris(2,2,2-trifluoroethyl)borate ($B(OCH_2CF_3)_3$) (0.40 mL, 1.80 mmol) was added to a solution of 2-(2,4-difluorophenyl)-3-(1*H*-1,2,4-triazol-1-yl)propanoic acid (**32**) (0.23 g, 0.90 mmol) and 4-nitrobenzylamine (**41**) (0.15 g, 0.99 mmol) in CPME (4 mL). The resulting mixture was then stirred at 100 °C overnight. Upon completion the reaction mixture was diluted with EtOAc (8 mL) and H_2O (1 mL). Amberlyst A-26(OH) (300 mg), Amberlyst 15 (300 mg) and Amberlite IRA743 (300 mg) were added together to the mixture, and it was stirred for 30 min. The mixture was then dried ($MgSO_4$), filtered to remove resins and $MgSO_4$ and washed with EtOAc (3 x 20 mL). The filtrate was concentrated *in vacuo* and an orange oil was obtained. The crude product was purified by gradient column chromatography to yield a faint yellow solid with 2.5 % MeOH in CH_2Cl_2. Yield: 0.23 g (67 %) as a yellow solid; mp 178-180°; TLC: CH_2Cl_2-MeOH 95:5 v/v, R_f 0.42. ^{1}H NMR (DMSO-*d$_6$*) δ: 8.90 (t, *J* = 5.9 Hz, 1H, NH), 8.35 (s, 1H, triaz), 8.12 (d, *J* = 8.8 Hz, 2H, Ar), 8.00 (s, 1H, triaz), 7.54 (q, *J* = 8.7 Hz, 1H, Ar), 7.26 (d, *J* = 8.9 Hz, 2H, Ar), 7.25 (t, *J* = 10.0 Hz, 1H, Ar), 7.11 (t, *J* = 8.5 Hz, 1H, Ar), 4.81 (dd, *J* = 8.4, 13.2 Hz,

1H, CH_2-triaz), 4.49 (m, 2H, CH_2-NH), 4.41 (dd, J = 6.2, 16.0 Hz, 1H, CH_2-triaz), 4.26 (dd, J = 5.5, 16.2 Hz, 1H, CH). ^{13}C NMR (DMSO-d_6) δ: 169.92 (C, C=O), 162.30 (dd, $^3J_{CF}$ = 12.6 Hz, $^1J_{CF}$ = 198.9 Hz, C, C2-Ar), 160.33 (dd, $^3J_{CF}$ = 12.2 Hz, $^1J_{CF}$ = 200.3 Hz, C, C4-Ar), 152.09 (CH, triaz), 147.54 (C, Ar), 146.84 (C, Ar), 145.10 (CH, triaz), 130.92 (dd, $^3J_{CF}$ = 5.0 Hz, $^3J_{CF}$ = 9.4 Hz, CH, C6-Ar), 128.24 (CH x 2, Ar), 123.80 (CH x 2, Ar), 120.43 (dd, $^4J_{CF}$ = 3.7 Hz, $^2J_{CF}$ = 15.0 Hz, CH, C1-Ar), 112.24 (dd, $^4J_{CF}$ = 3.3 Hz, $^2J_{CF}$ = 21.1 Hz, CH, C5-Ar), 104.44 (t, $^2J_{CF}$ = 26.2 Hz, CH, C3-Ar), 50.42 ($\underline{C}H_2$-triaz), 43.99 ($\underline{C}$H), 42.21 ($\underline{C}H_2$-NH). HPLC: 100 %, RT = 4.35 min. HRMS (ESI) m/z: theoretical mass: 388.1221[M+H]$^+$, observed mass: 388.1221[M+H]$^+$.

N-(4-Aminobenzyl)-2-(2,4-difluorophenyl)-3-(1H-1,2,4-triazol-1-yl) propenamide (43)

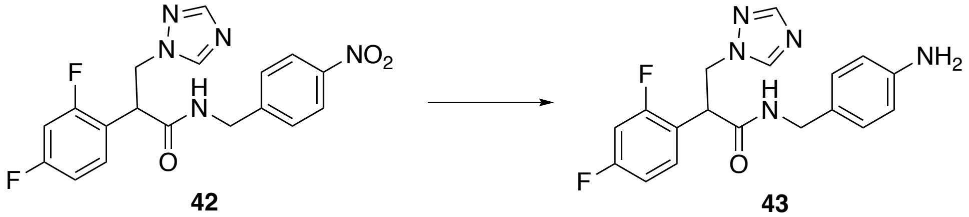

Method: as described for 4-amino-N-(2-(2,4-difluorophenyl)-2-hydroxy-3-(1H-1,2,4-triazol-1-yl)propyl) benzamide (**25**).

Prepared from 2-(2,4-difluorophenyl)-N-(4-nitrobenzyl)-3-(1H-1,2,4-triazol-1-yl) propenamide (**42**) (0.18 g, 0.46 mmol) and 10 % Pd-C (20 mg). The residue was purified by gradient column chromatography and the desired compound eluted with 3 % MeOH in CH_2Cl_2. Yield: 0.15 g (93 %) as a white solid; mp 147-149°; TLC: CH_2Cl_2-MeOH 95:5 v/v, R_f 0.30. ^{1}H NMR (DMSO-d_6) δ: 8.53 (t, J = 5.8 Hz, 1H, NH), 8.33 (s, 1H, triaz), 7.92 (s, 1H, triaz), 7.56 (q, J = 8.7 Hz, 1H, Ar), 7.20 (t, J = 7.8 Hz, 1H, Ar), 7.08 (t, J = 8.5 Hz, 1H, Ar), 6.69 (d, J = 8.5 Hz, 2H, Ar), 6.43 (d, J = 8.5 Hz, 2H, Ar), 4.92 (s, 2H, NH_2), 4.76 (dd, J = 10.6, 15.7 Hz, 1H, CH_2-triaz), 4.43 (m, 2H, CH_2-NH), 4.07 (dd, J = 6.0, 15.0 Hz, 1H, CH_2-triaz), 3.98 (dd, J = 5.6, 15.0 Hz, 1H, CH). ^{13}C NMR (DMSO-d_6) δ: 169.17 (C, C=O), 162.50 (dd, $^3J_{CF}$ =

12.2 Hz, $^{1}J_{CF}$ = 182.7 Hz, C, C2-Ar), 160.27 (dd, $^{3}J_{CF}$ = 12.0 Hz, $^{1}J_{CF}$ = 184.6 Hz, C, C4-Ar), 151.93 (CH, triaz), 147.95 (C, Ar), 144.99 (CH, triaz), 130.94 (dd, $^{3}J_{CF}$ = 5.2 Hz, $^{3}J_{CF}$ = 9.53 Hz, CH, C6-Ar), 128.39 (CH x 2, Ar), 126.07 (C, Ar), 120.82 (dd, $^{4}J_{CF}$ = 3.6 Hz, $^{2}J_{CF}$ = 15.0 Hz, CH, C1-Ar), 114.05 (CH x 2, Ar), 112.06 (dd, $^{4}J_{CF}$ = 3.5 Hz, $^{2}J_{CF}$ = 21.3 Hz, CH, C5-Ar), 104.29 (t, $^{2}J_{CF}$ = 26.5 Hz, CH, C3-Ar), 50.59 ($\underline{C}H_2$-triaz), 44.05 ($\underline{C}H$), 42.40 ($\underline{C}H_2$-NH). HPLC: 100 %, RT = 3.77 min. HRMS (ESI) m/z: theoretical mass: 358.1479 $[M+H]^+$, observed mass: 358.1480 $[M+H]^+$.

COMPUTATIONAL METHODS

Molecular Modelling. The crystal structure of the wild-type CaCYP51 (PDB 5FSA) was downloaded from the Protein Data Bank.[17] All compounds were protonated at physiological pH (7.4), to mimic the physiological condition in *C. albicans* endoplasmic reticulum and used in the docking studies. The MOE programme[18] was used to perform molecular docking and was found to closely replicate the position and binding interactions of posaconazole, as observed in the crystal structure. All minimisations were performed with MOE with MMFF94 forcefield and partial charges were automatically calculated. The charge of the haem iron at physiological pH was set to 3^+ (geometry d2sp3) through the atom manager in MOE. The London ΔG scoring function estimates the free energy of binding of the ligand from a given pose. Refinement of the results using MMFF94 forcefield and scoring of the refined results using the London ΔG scoring function was applied. The output database dock file was created with different poses for each ligand and arranged according to the final S-score function, which is the score of the last stage that was not set to zero. The single and double mutants were prepared using the protein builder function in Molecular Operating Environment software[18] to mutate Y132 and K143 in the wild-type crystal structure (PDB 5FSA) followed by energy

minimisation of the side chains, as described above, prior to molecular dynamics simulation of the protein *S*-**22** complex.

Molecular Dynamics (MD) Simulation. Molecular dynamics simulations were performed on CaCYP51-ligand complexes, which were optimised with protein preparation wizard in Maestro (Schrödinger release 2020-1),[19] by assigning bond orders, adding hydrogen, and correcting incorrect bond types. A default quick relaxation protocol was used to minimise the MD systems with the Desmond programme.[19,20] In Desmond, the volume of space in which the simulation takes place, the global cell, is built up by regular 3D simulation boxes, which was utilised as part of this system for protein interactions. The orthorhombic water box allowed for a 10 Å buffer region between protein atoms and box sides. Overlapping water molecules were deleted, and the systems were neutralised with Na^+ ions and salt concentration 0.15 M. Force-field parameters for the complexes were assigned using the OPLS_ 2005 forcefield, that is a 150 ns molecular dynamic run in the NPT ensemble (T = 300 K) at a constant pressure of 1 bar. Energy and trajectory atomic coordinate data were recorded at each 1.2 ns.

^{1}H, ^{13}C (APT), ^{19}F NMR and HPLC or elemental analysis of final compounds

Compound 11

^{1}H NMR (DMSO-d_6)

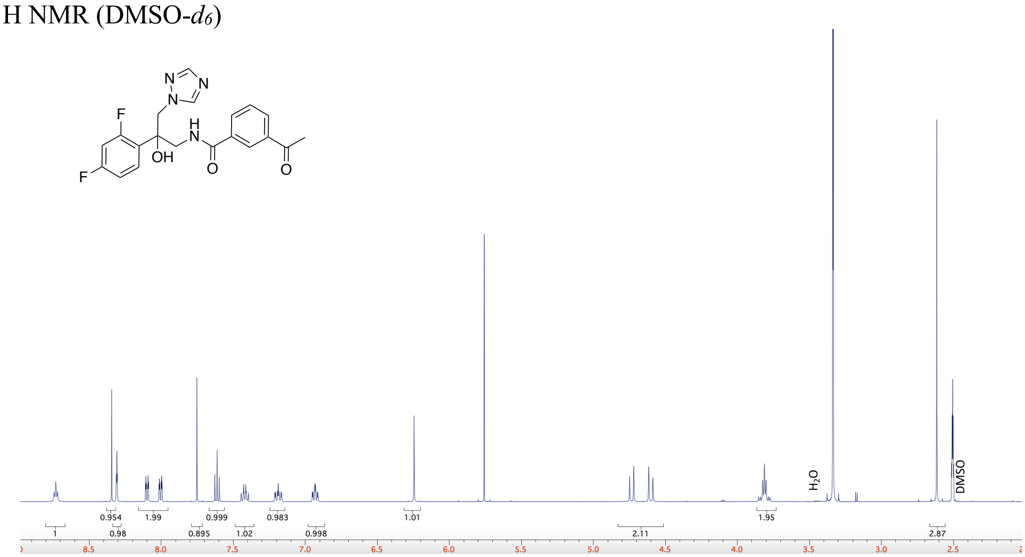

^{19}F NMR (DMSO-d_6)	^{13}C NMR (DMSO-d_6)
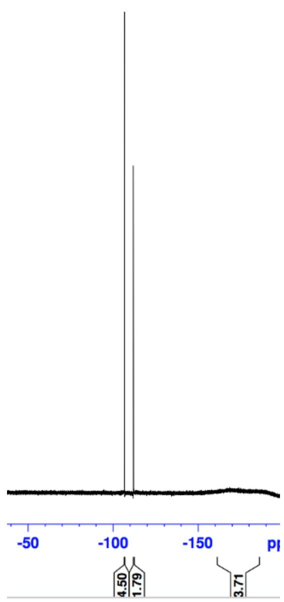	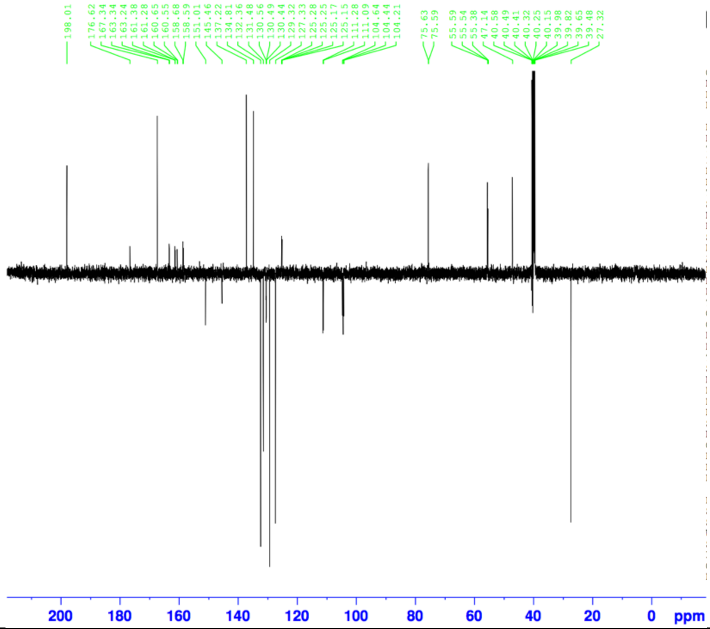

HPLC

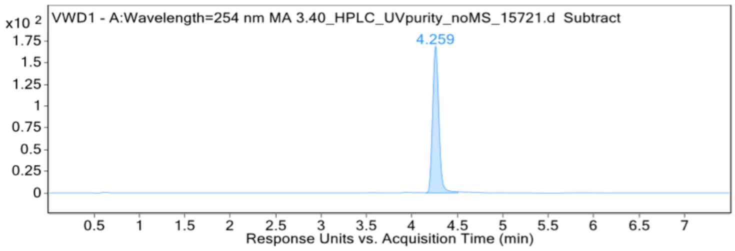

Figure: Base peak or HPLC chromatogram (indicated in left hand corner)

User Chromatogram Peak List

RT (min)	Area	Area %	Area Sum (%)	Symmetry	Width (min)
4.26	813.24	100.00	100.00	1.18	0.353

Compound 12

^{1}H NMR (DMSO-d_6)

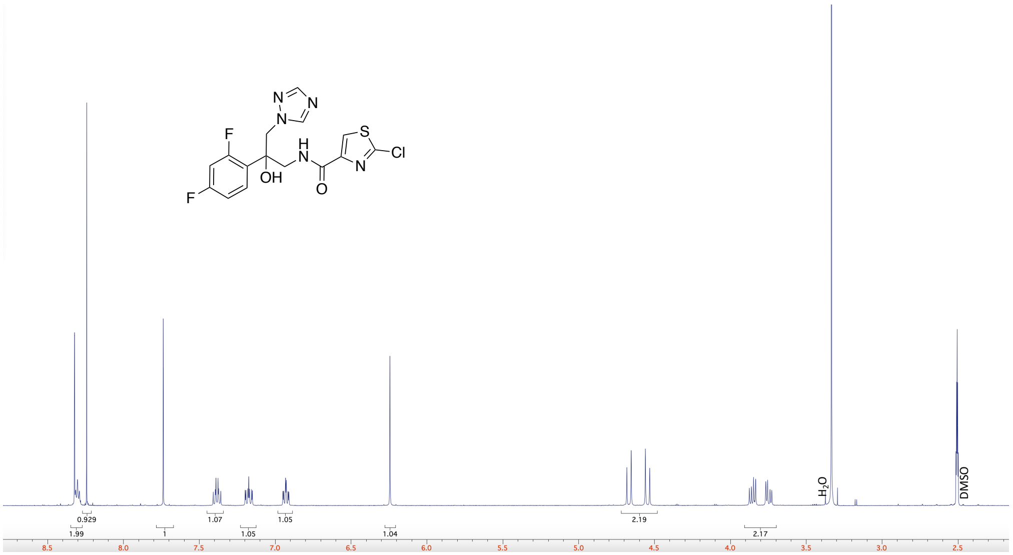

^{19}F NMR (DMSO-d_6)	^{13}C NMR (DMSO-d_6)
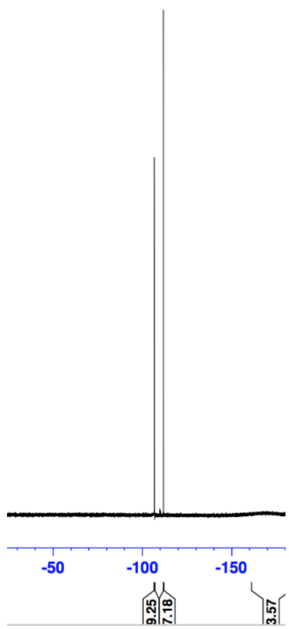	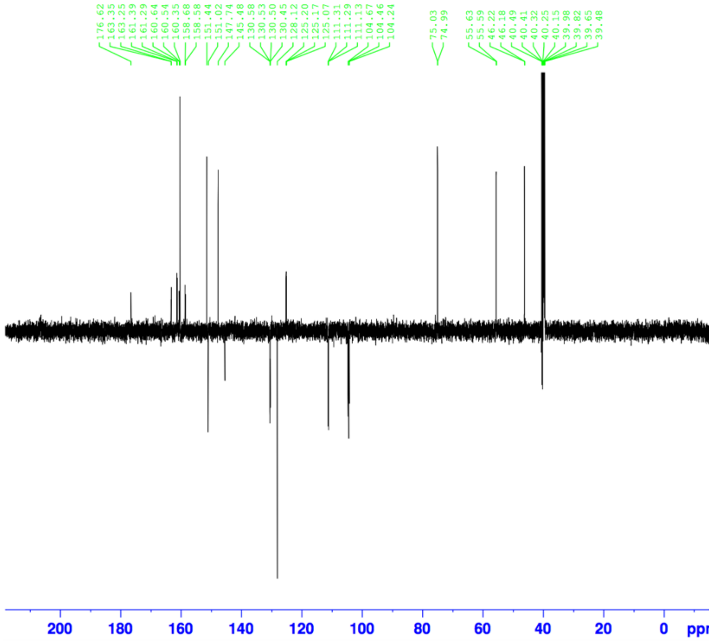

HPLC

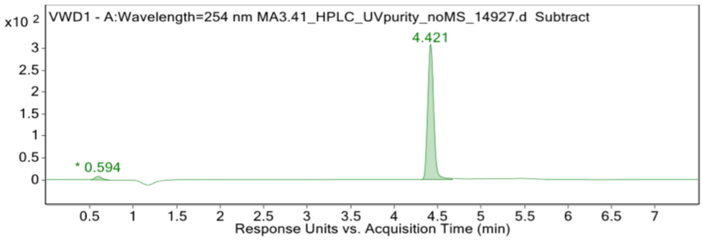

Figure: Base peak or HPLC chromatogram (indicated in left hand corner)

User Chromatogram Peak List

RT (min)	Area	Area %	Area Sum (%)	Symmetry	Width (min)
0.59	44.9	3.02	2.93	1.27	0.220
4.42	1485.35	100.00	97.07	1.07	0.353

Compound 13

^{1}H NMR (DMSO-d_6)

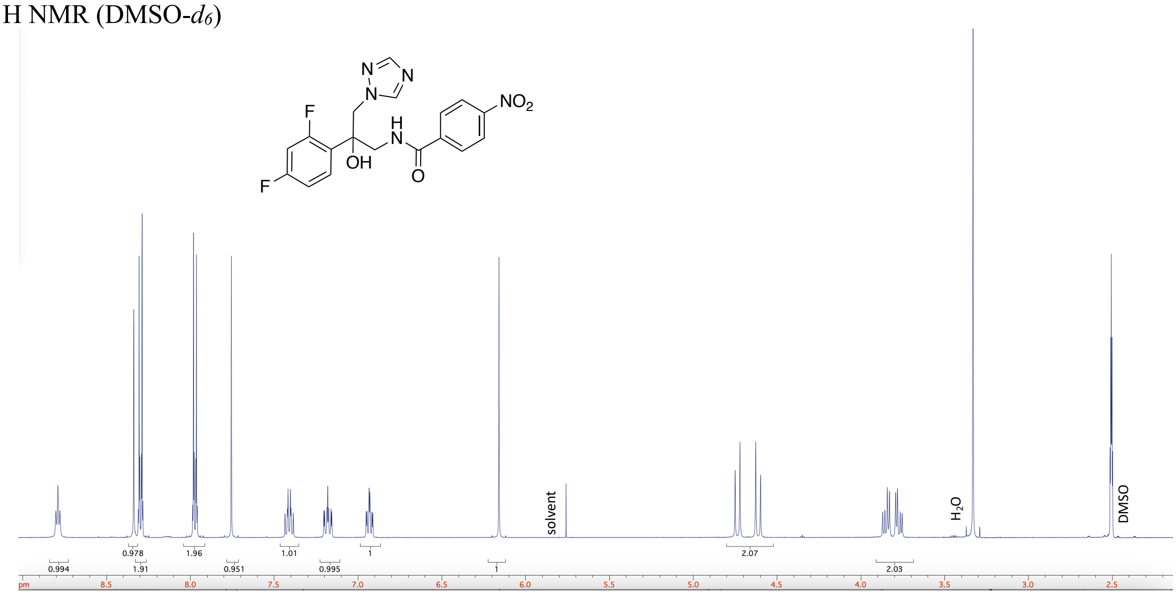

^{19}F NMR (DMSO-d_6)	^{13}C NMR (DMSO-d_6)
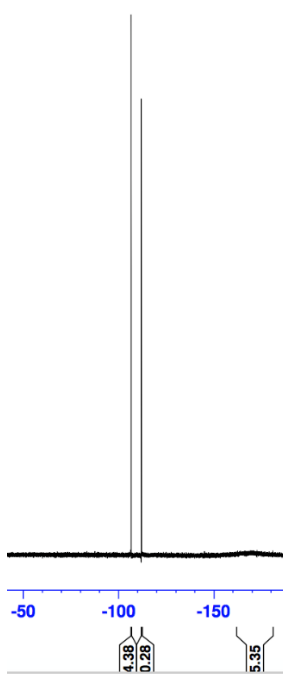	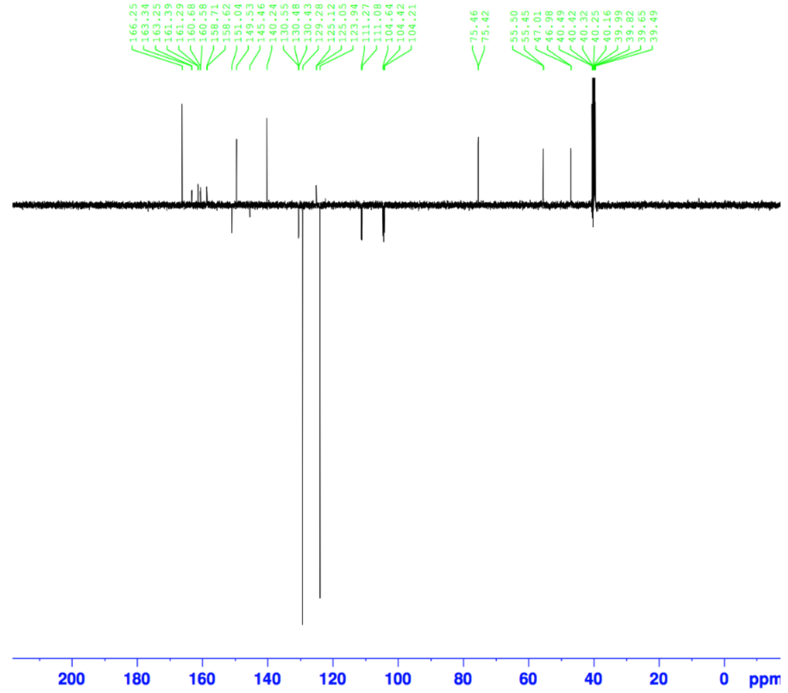

HPLC

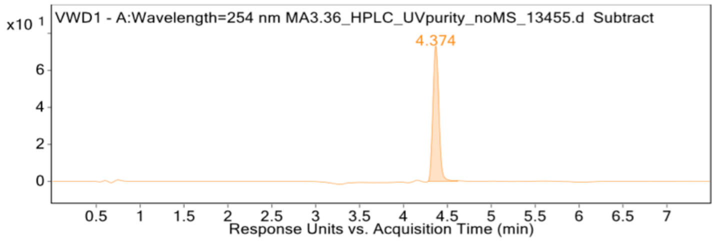

Figure: Base peak or HPLC chromatogram (indicated in left hand corner)

User Chromatogram Peak List

RT (min)	Area	Area %	Area Sum (%)	Symmetry	Width (min)
4.37	346.03	100.00	100.00	1.03	0.336

Compound 14

^{1}H NMR (DMSO-d_6)

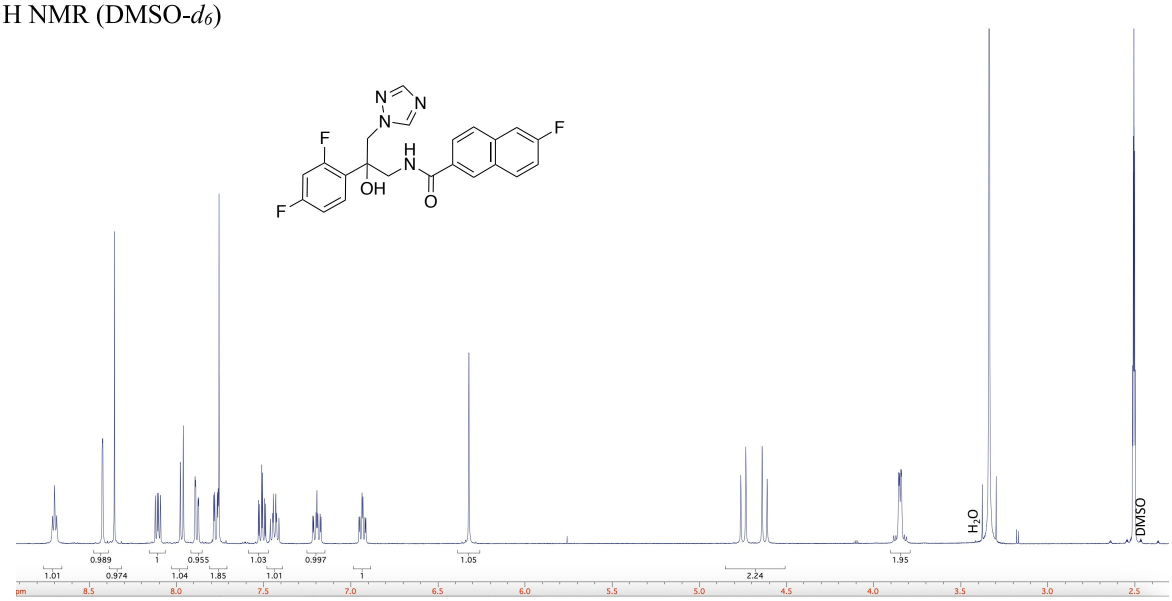

^{19}F NMR (DMSO-d_6)	^{13}C NMR (DMSO-d_6)
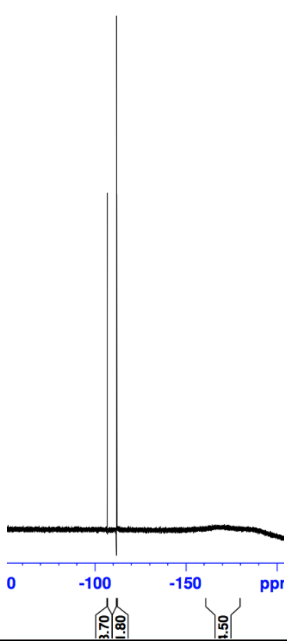	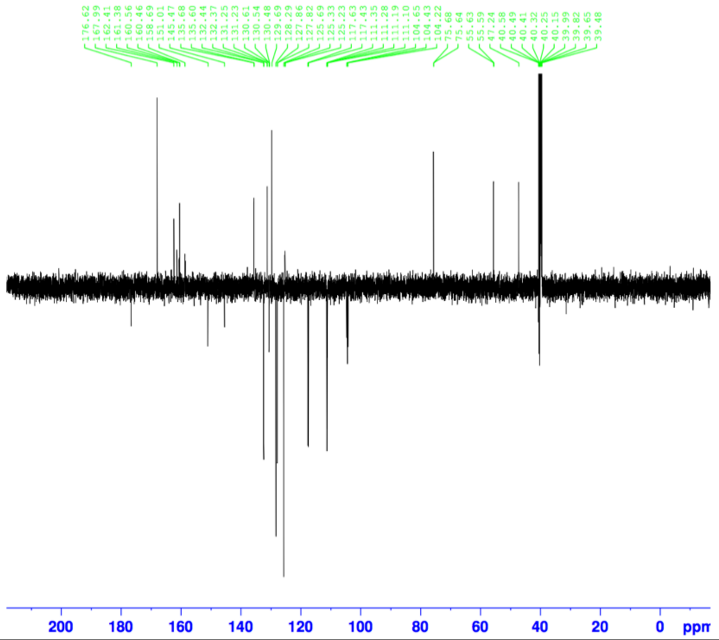

HPLC

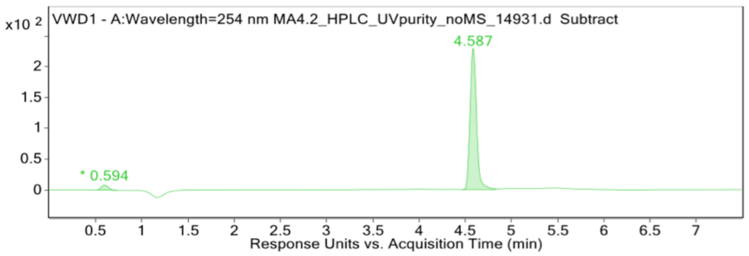

Figure: Base peak or HPLC chromatogram (indicated in left hand corner)

User Chromatogram Peak List

RT (min)	Area	Area %	Area Sum (%)	Symmetry	Width (min)
0.59	44.16	3.90	3.75	1.27	0.220
4.59	1133.27	100.00	96.25	1.09	0.360

Compound 15

^{1}H NMR (DMSO-d_6)

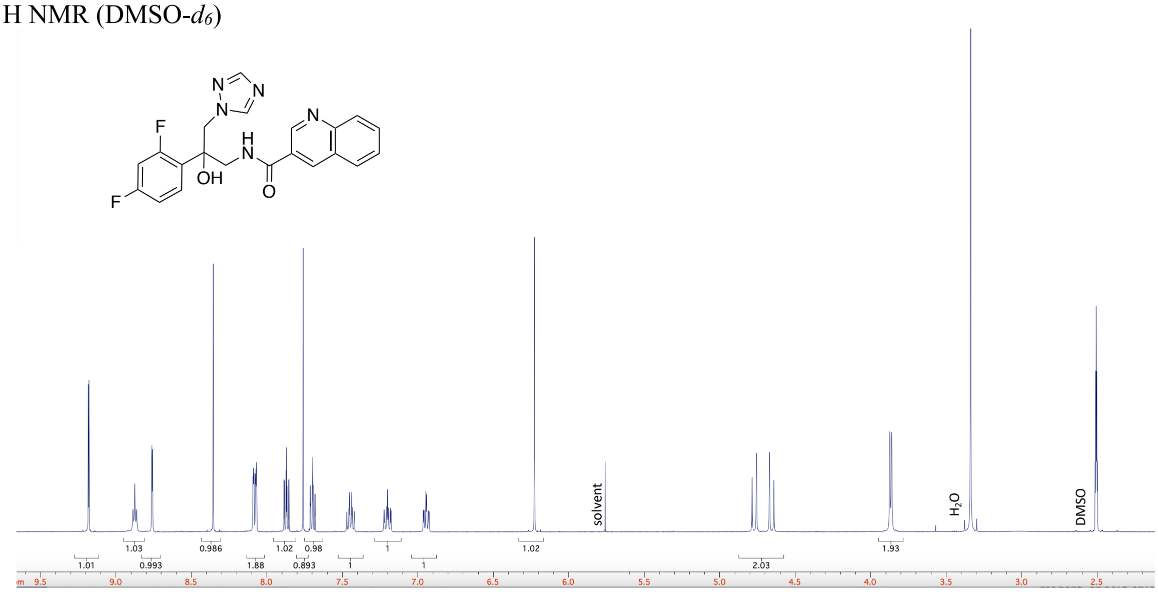

^{19}F NMR (DMSO-d_6)	^{13}C NMR (DMSO-d_6)
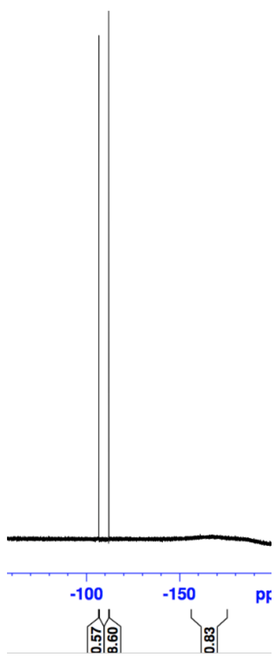	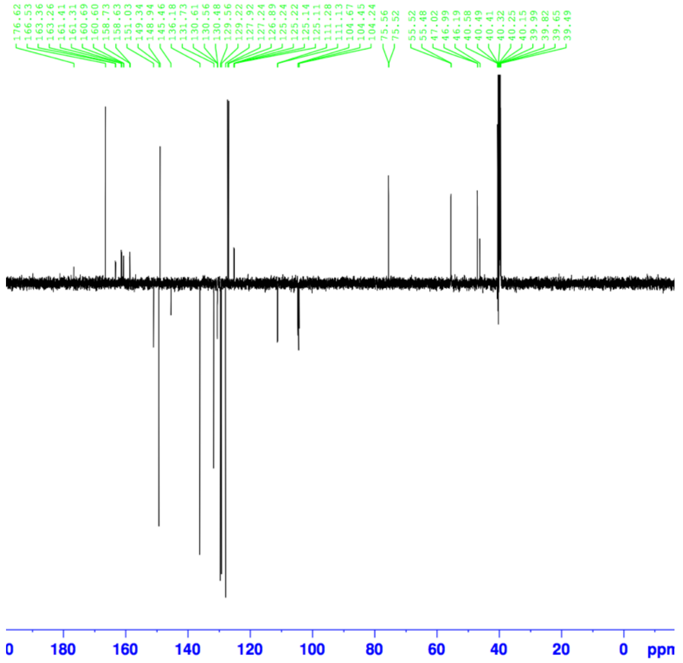

HPLC

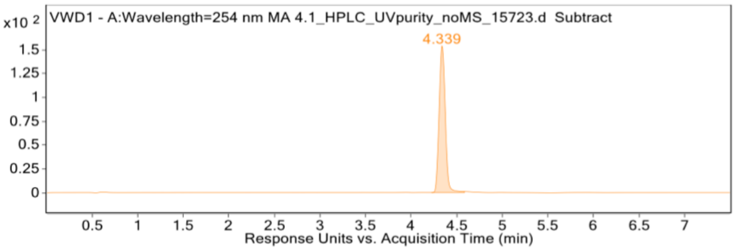

Figure: Base peak or HPLC chromatogram (indicated in left hand corner)

User Chromatogram Peak List

RT (min)	Area	Area %	Area Sum (%)	Symmetry	Width (min)
4.34	726.45	100.00	100.00	1.14	0.360

Compound 16
^{1}H NMR (DMSO-d_6)

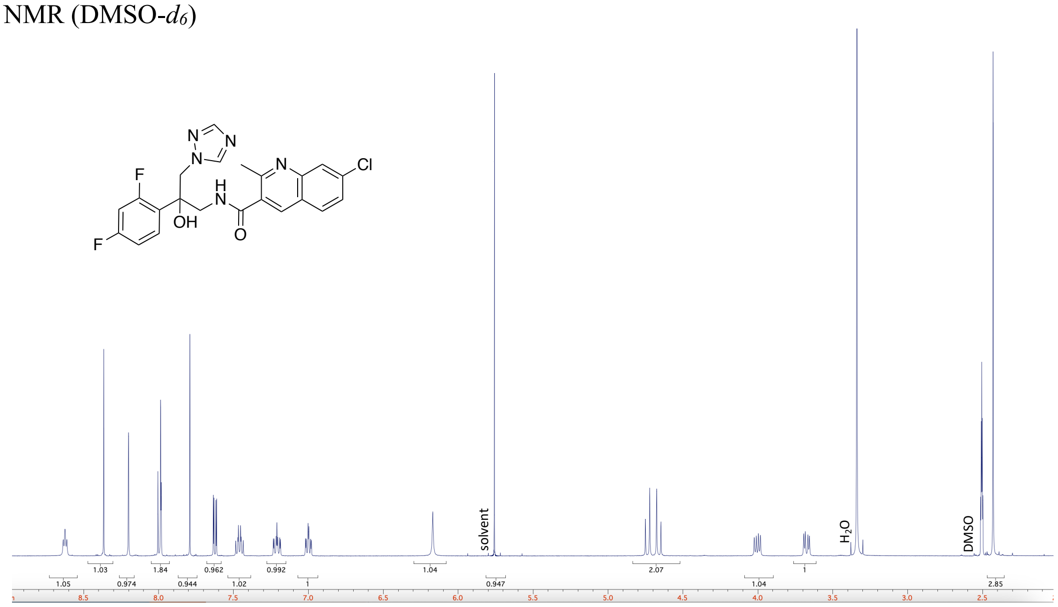

^{19}F NMR (DMSO-d_6)	^{13}C NMR (DMSO-d_6)
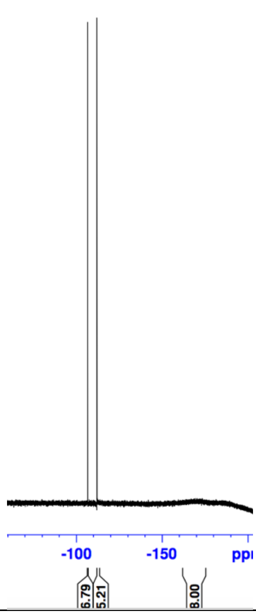	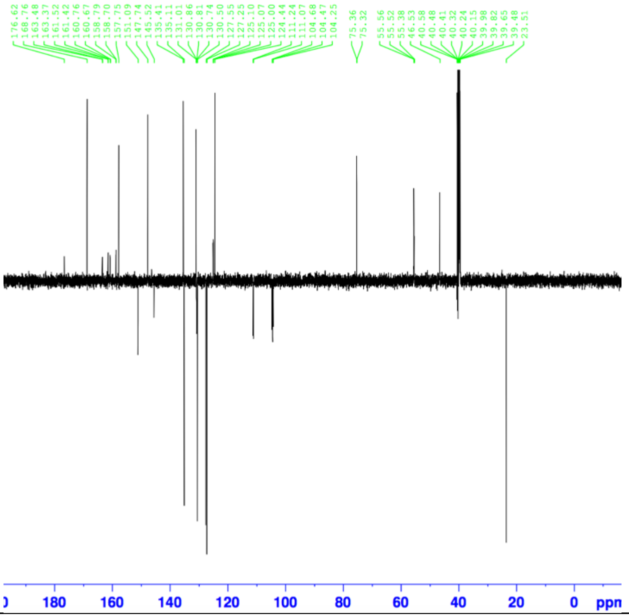

HPLC

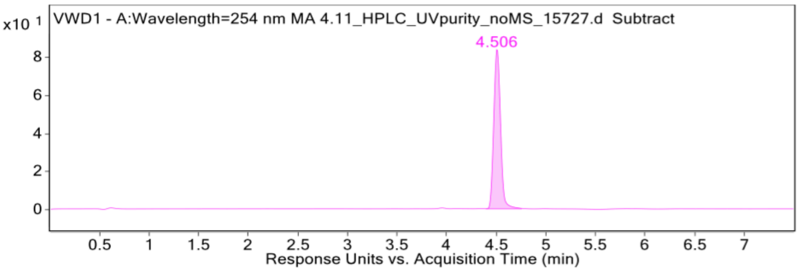

Figure: Base peak or HPLC chromatogram (indicated in left hand corner)

User Chromatogram Peak List

RT (min)	Area	Area %	Area Sum (%)	Symmetry	Width (min)
4.51	398.61	100.00	100.00	1.19	0.353

Compound 21

^{1}H NMR (DMSO-d_6)

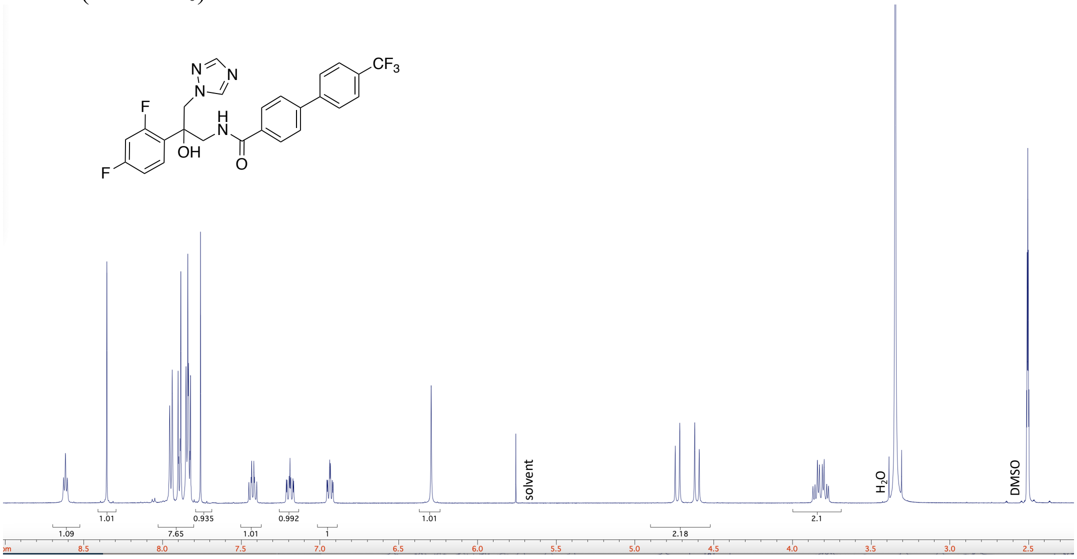

^{19}F NMR (DMSO-d_6)	^{13}C NMR (DMSO-d_6)
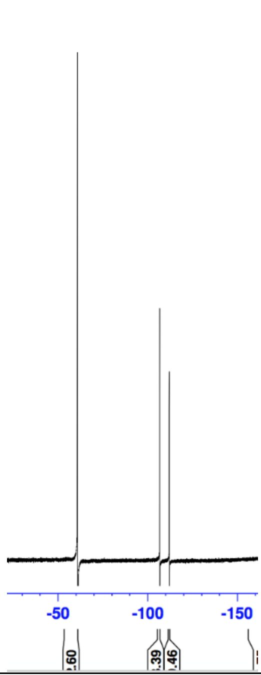	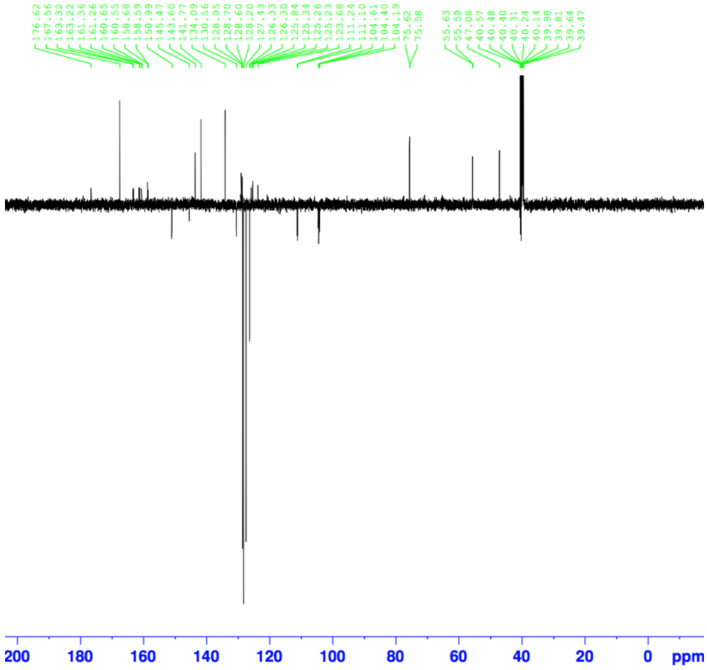

HPLC

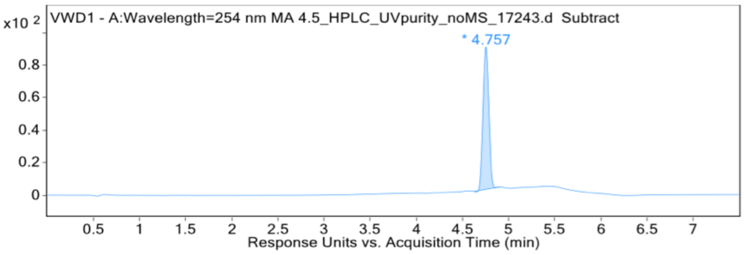

Figure: Base peak or HPLC chromatogram (indicated in left hand corner)

User Chromatogram Peak List

RT (min)	Area	Area %	Area Sum (%)	Symmetry	Width (min)
4.76	389.64	100.00	100.00	0.99	0.287

Compound 22

^{1}H NMR (DMSO-d_6)

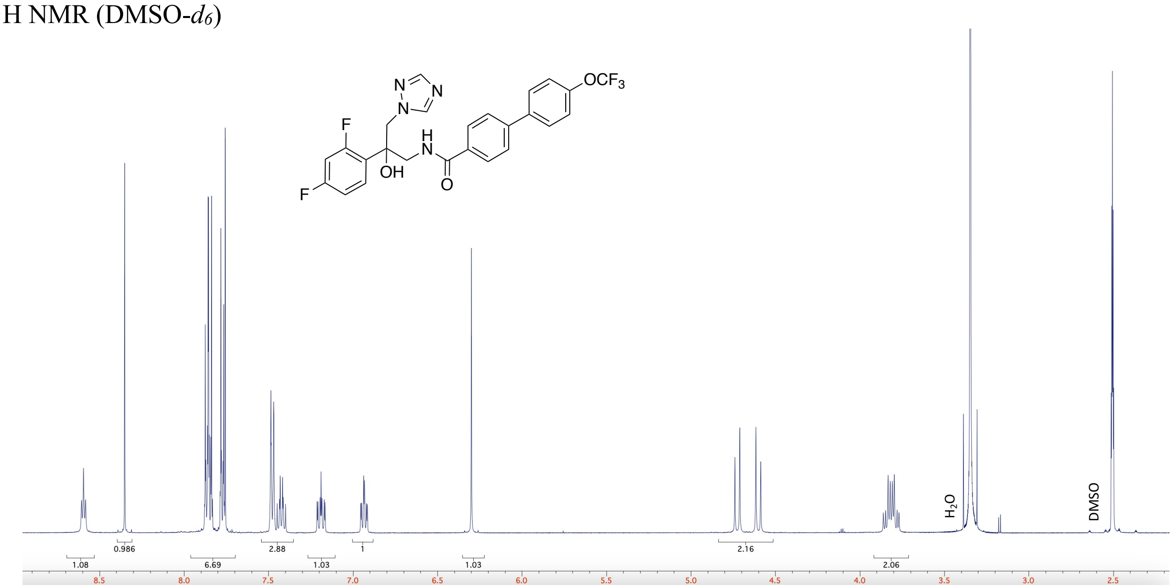

^{19}F NMR (DMSO-d_6)

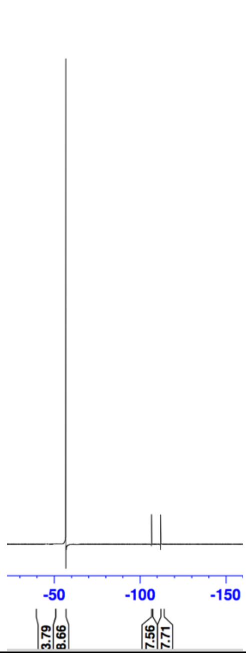

^{13}C NMR (DMSO-d_6)

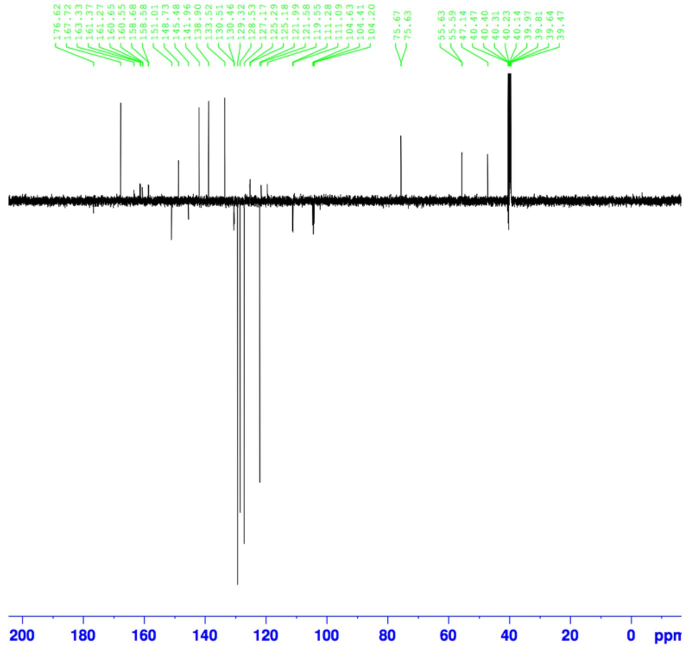

HPLC

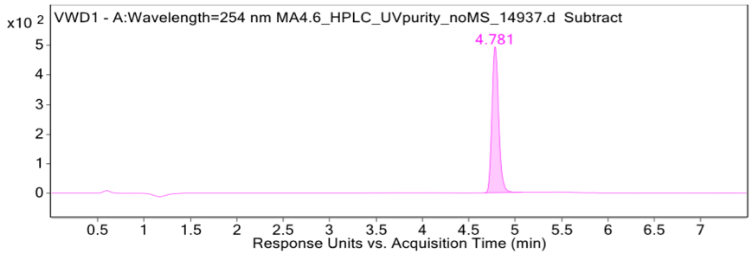

Figure: Base peak or HPLC chromatogram (indicated in left hand corner)

User Chromatogram Peak List

RT (min)	Area	Area %	Area Sum (%)	Symmetry	Width (min)
4.78	2513.54	100.00	100.00	1.34	0.400

Compound 23

^{1}H NMR (DMSO-d_6)

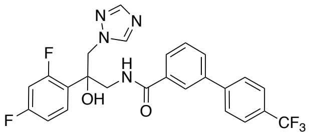

23

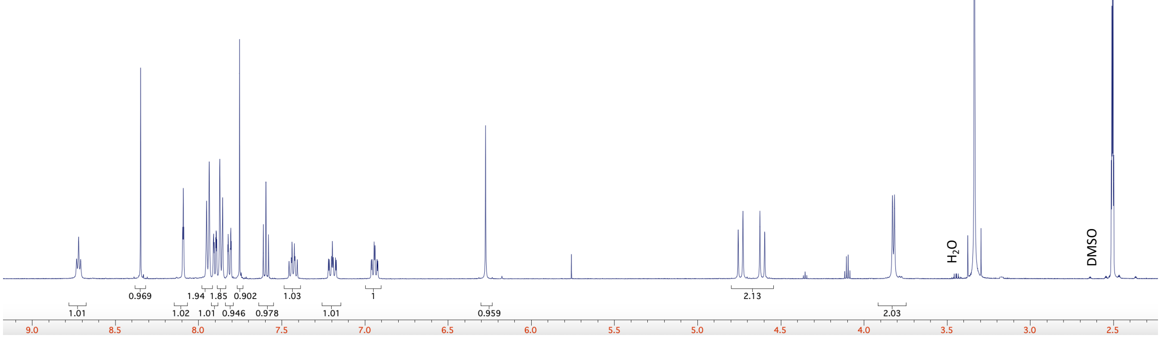

^{19}F NMR (DMSO-d_6)	^{13}C NMR (DMSO-d_6)
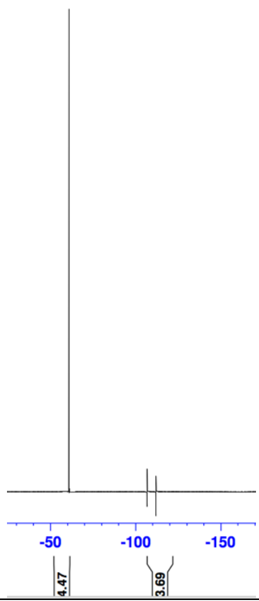	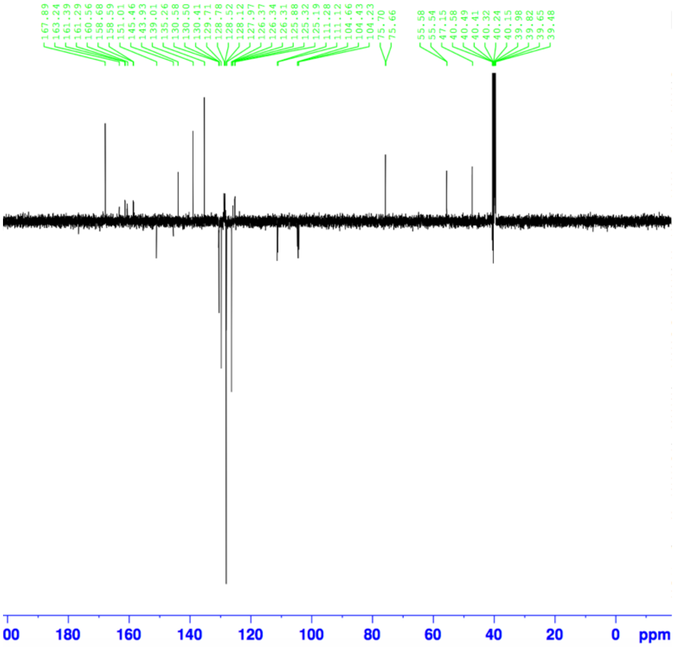

HPLC

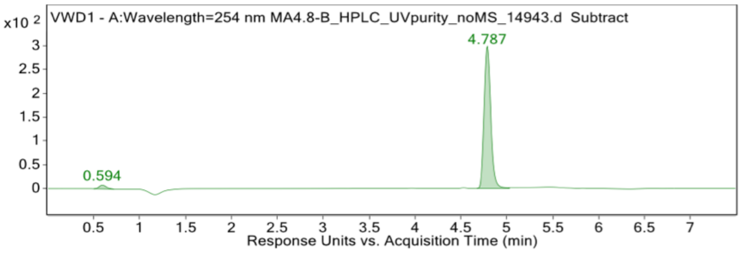

Figure: Base peak or HPLC chromatogram (indicated in left hand corner)

User Chromatogram Peak List

RT (min)	Area	Area %	Area Sum (%)	Symmetry	Width (min)
0.59	44.11	2.91	2.82	1.23	0.221
4.79	1518.29	100.00	97.18	1.22	0.353

Compound 24

^{1}H NMR (DMSO-d_6)

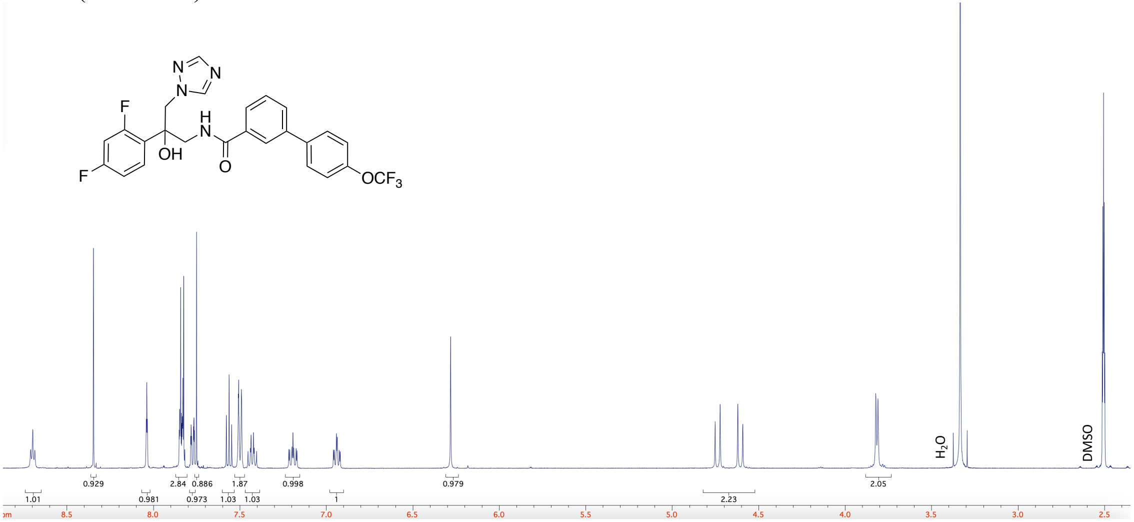

^{19}F NMR (DMSO-d_6)	^{13}C NMR (DMSO-d_6)

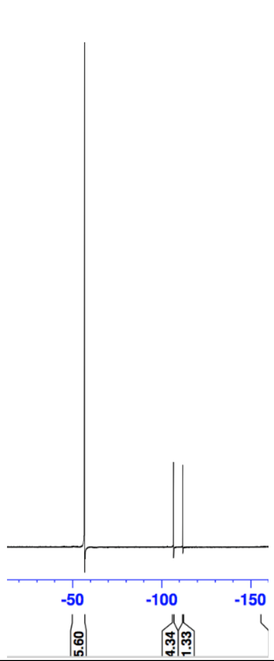

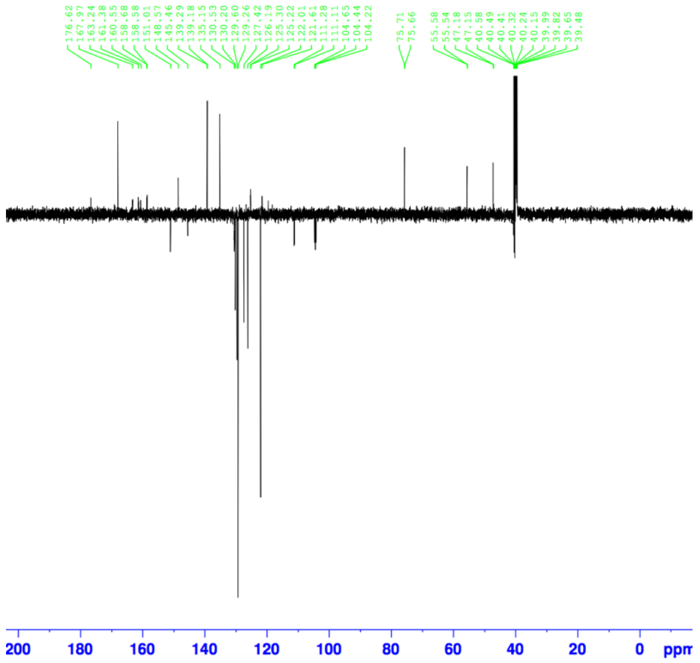

HPLC

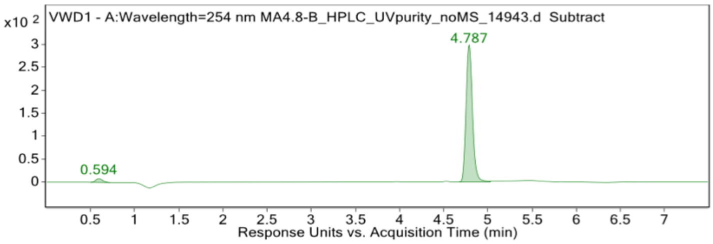

Figure: Base peak or HPLC chromatogram (indicated in left hand corner)

User Chromatogram Peak List

RT (min)	Area	Area %	Area Sum (%)	Symmetry	Width (min)
0.59	44.11	2.91	2.82	1.23	0.221
4.79	1518.29	100.00	97.18	1.22	0.353

Compound 26

^{1}H NMR (DMSO-d_6)

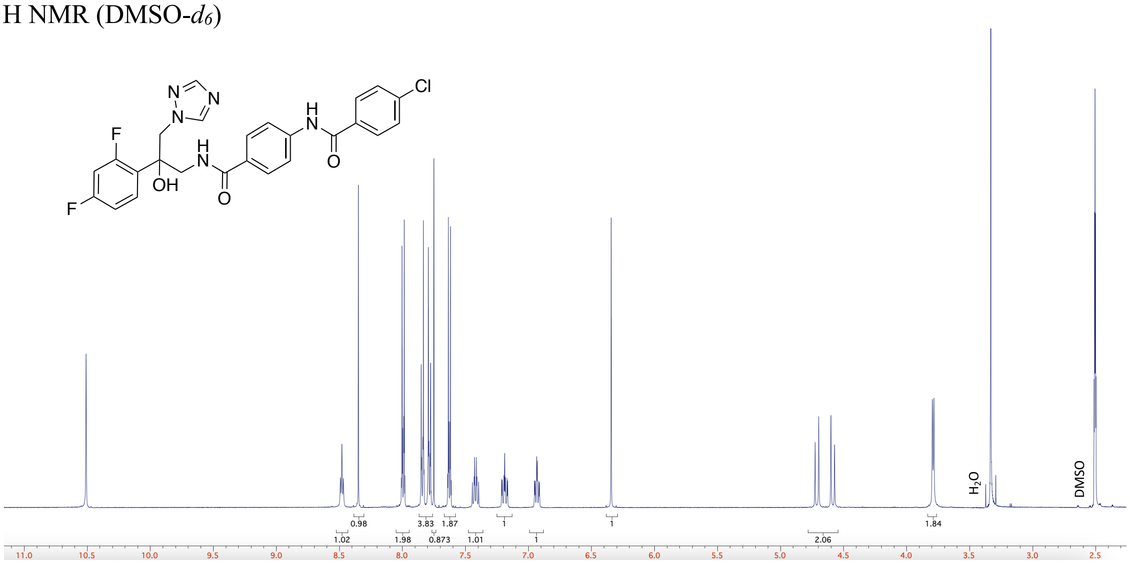

^{19}F NMR (DMSO-d_6)	^{13}C NMR (DMSO-d_6)
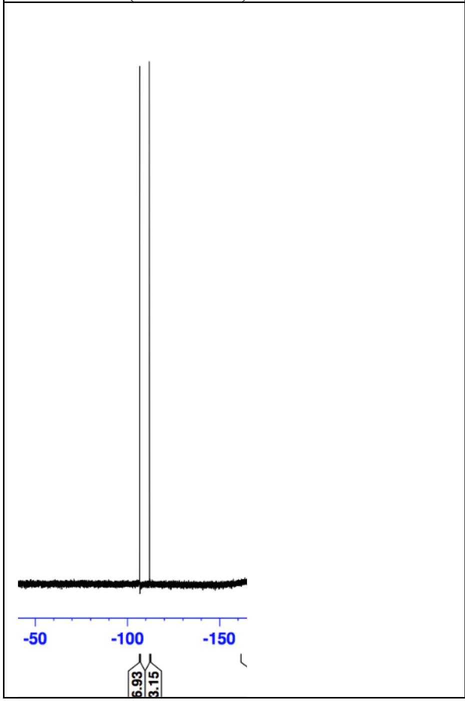	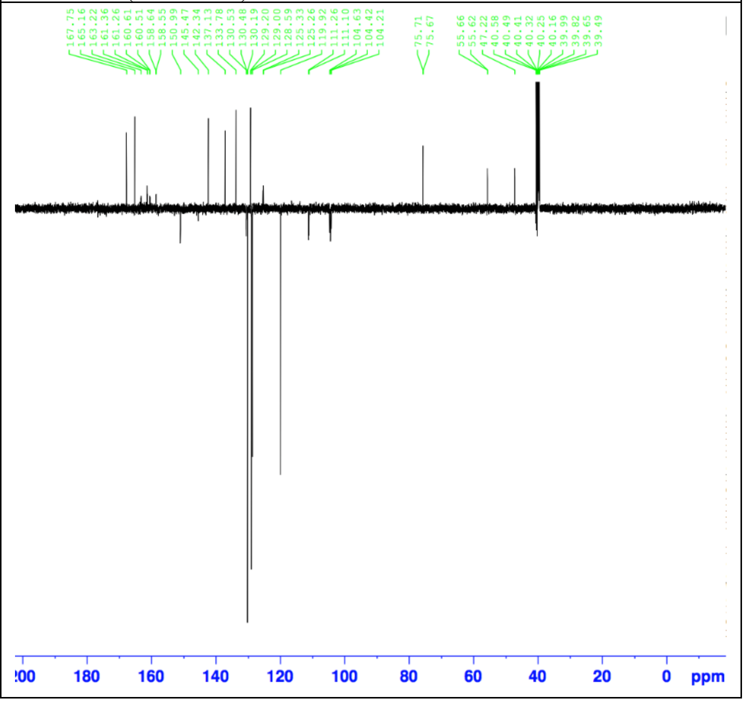

HPLC

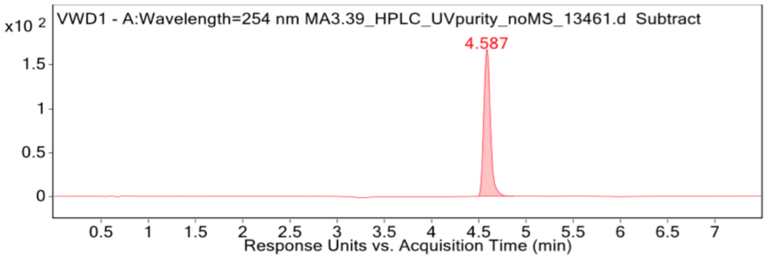

Figure: Base peak or HPLC chromatogram (indicated in left hand corner)

User Chromatogram Peak List

RT (min)	Area	Area %	Area Sum (%)	Symmetry	Width (min)
4.59	824.78	100.00	100.00	1.31	0.371

Compound 37

^{1}H NMR (DMSO-d_6)

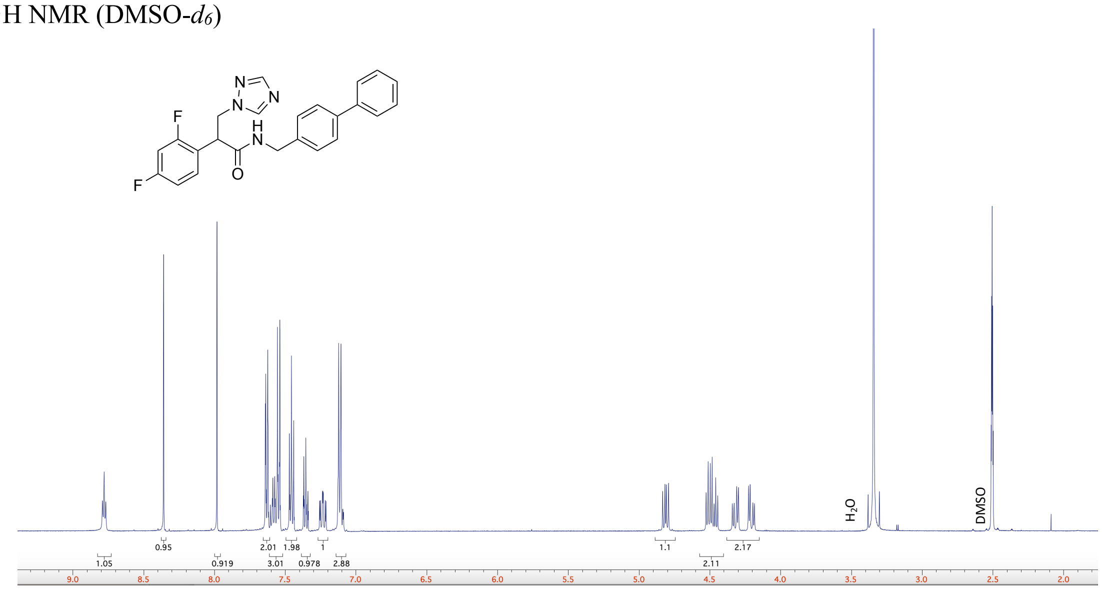

^{19}F NMR (DMSO-d_6)	^{13}C NMR (DMSO-d_6)
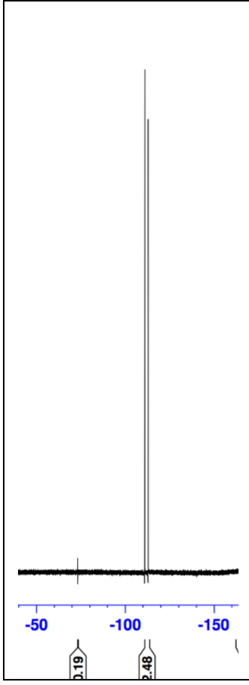	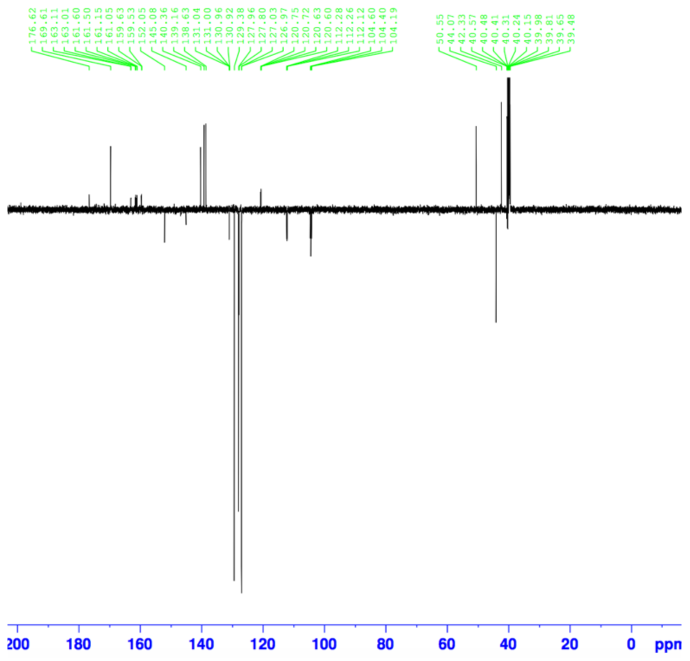

HPLC

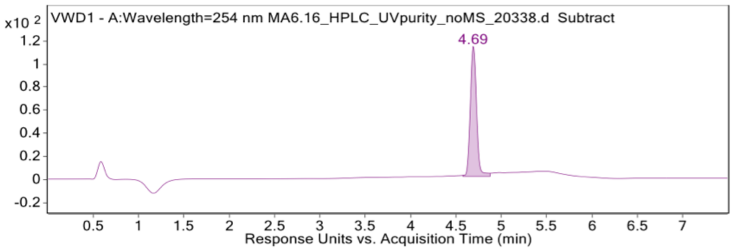

Figure: Base peak or HPLC chromatogram (indicated in left hand corner)

User Chromatogram Peak List

RT (min)	Area	Area %	Area Sum (%)	Symmetry	Width (min)
4.69	543.56	100.00	100.00	1.17	0.300

Compound 38

^{1}H NMR (DMSO-d_6)

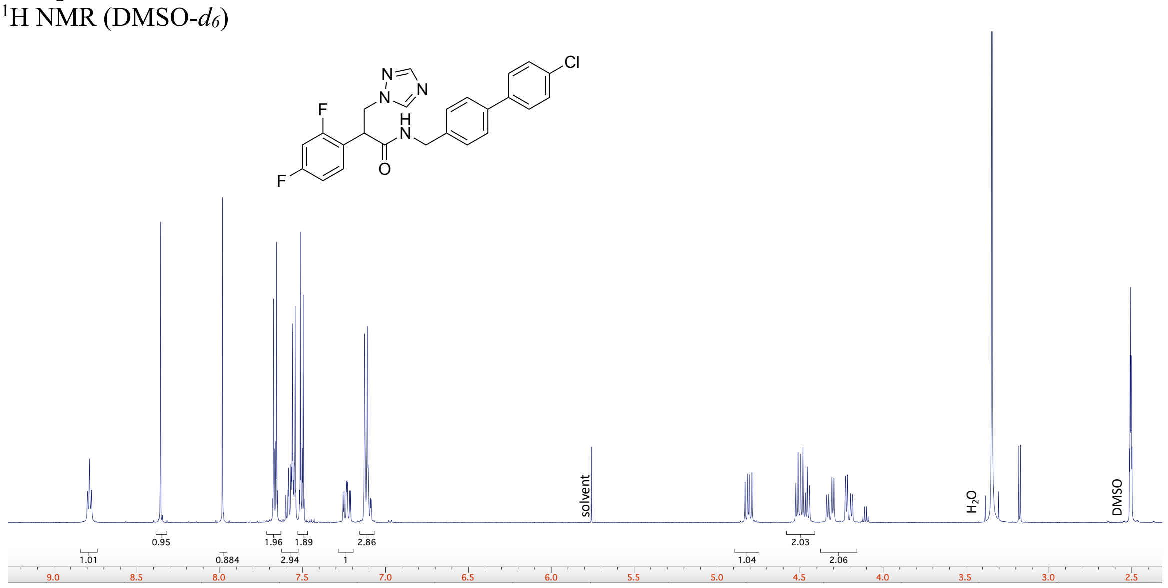

^{19}F NMR (DMSO-d_6)	^{13}C NMR (DMSO-d_6)

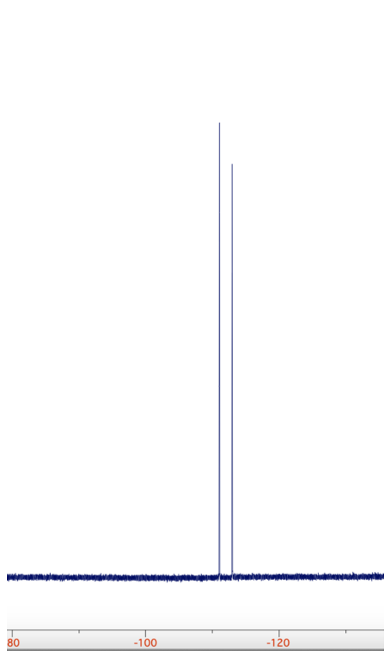

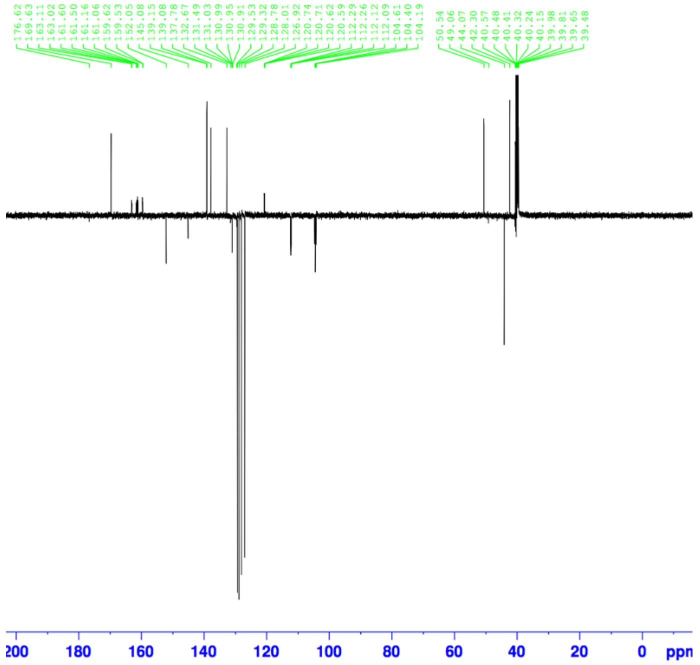

HPLC

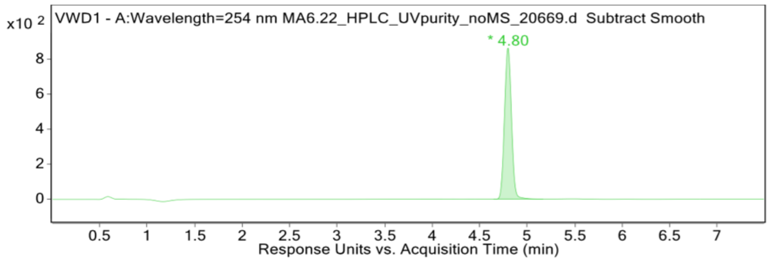

Figure: Base peak or HPLC chromatogram (indicated in left hand corner)

User Chromatogram Peak List

RT (min)	Area	Area %	Area Sum (%)	Symmetry	Width (min)
4.80	4422.08	100.00	100.00	0.97	0.520

Compound 39

^{1}H NMR (DMSO-d_6)

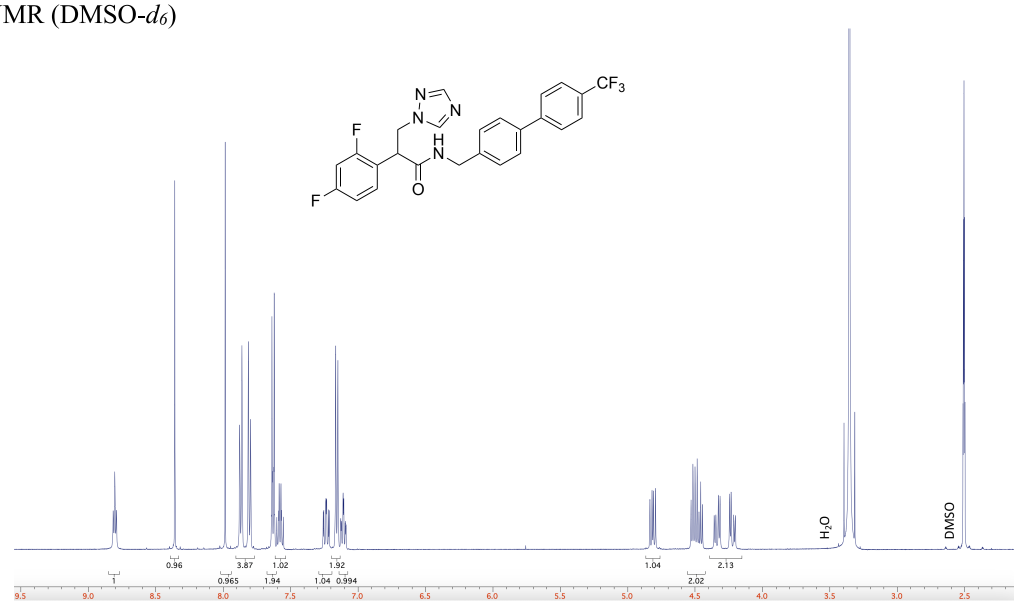

^{19}F NMR (DMSO-d_6)	^{13}C NMR (DMSO-d_6)
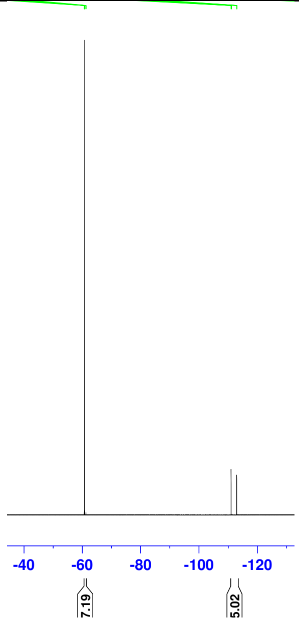	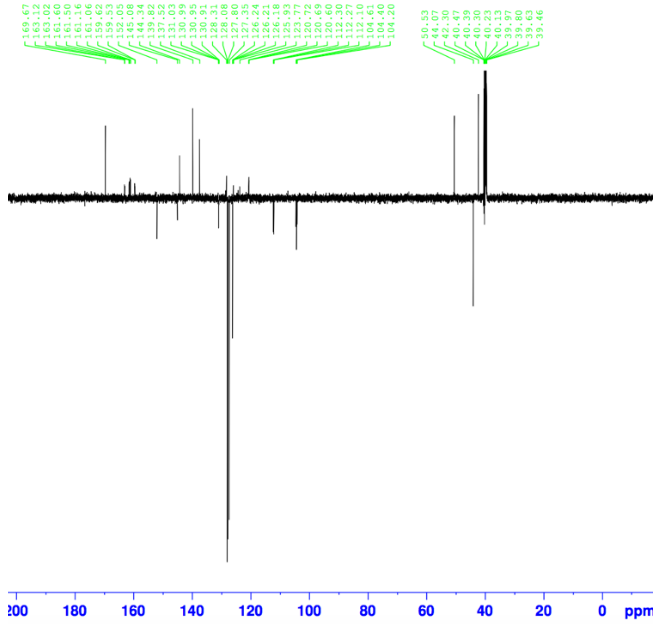

HPLC

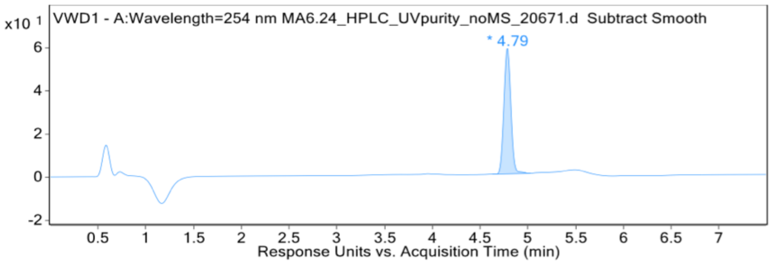

Figure: Base peak or HPLC chromatogram (indicated in left hand corner)

User Chromatogram Peak List

RT (min)	Area	Area %	Area Sum (%)	Symmetry	Width (min)
4.79	297.13	100.00	100.00	0.98	0.400

Compound 40

^{1}H NMR (DMSO-*d₆*)

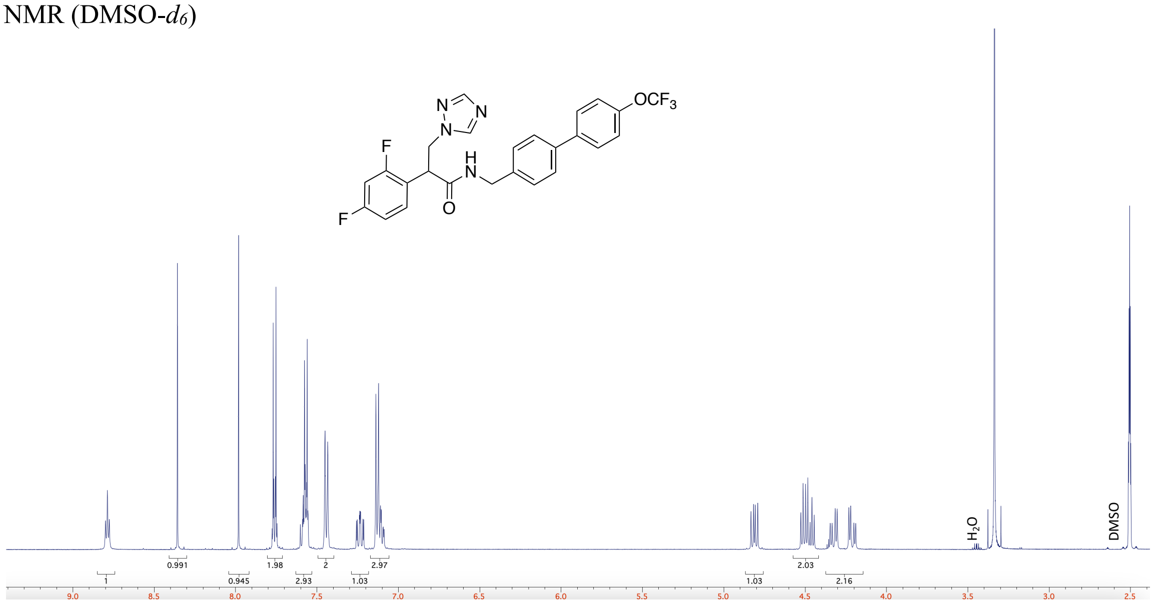

^{19}F NMR (DMSO-*d₆*)	^{13}C NMR (DMSO-*d₆*)
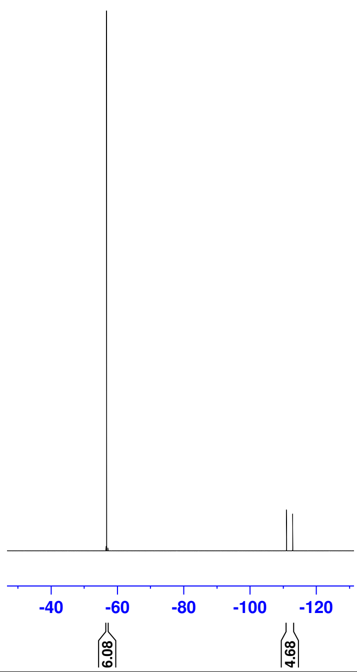	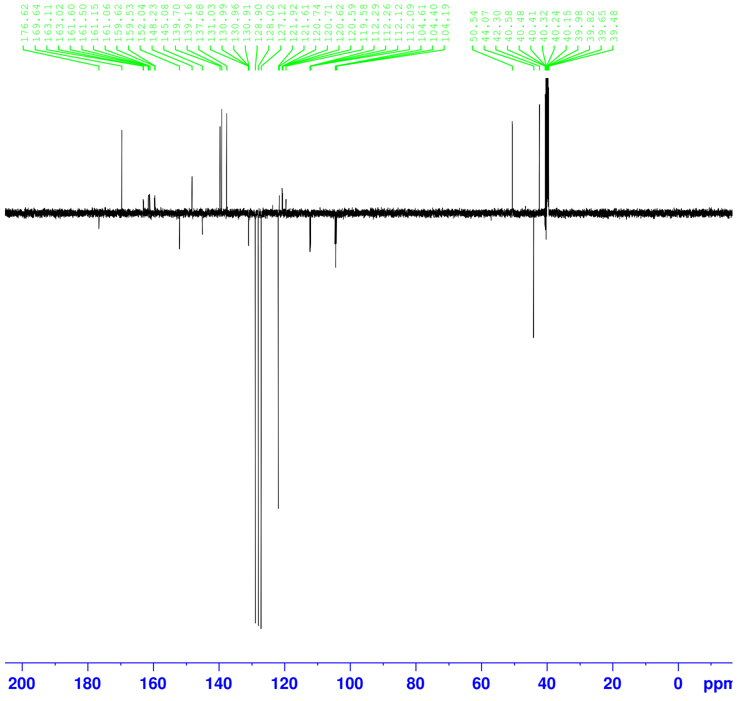

Elemental Analysis

Formula $C_{25}H_{19}F_5N_4O_2$

ELEMENT	C	H	N
% Theory	59.76	3.81	11.15
% Found 1	59.72	3.69	11.18
% Found 2			

Compound 44

^{1}H NMR (DMSO-d_6)

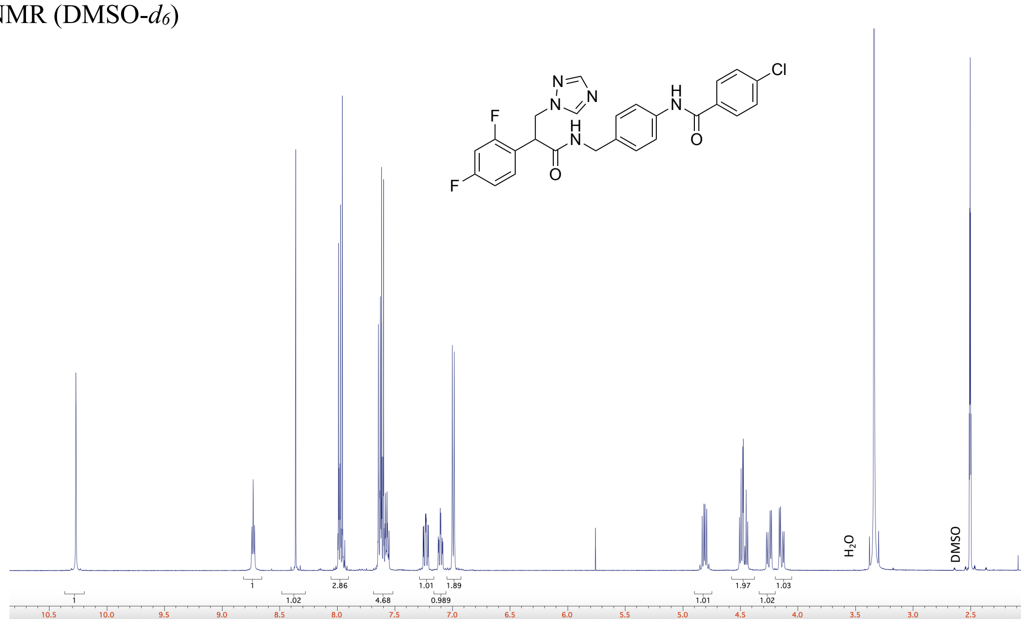

^{19}F NMR (DMSO-d_6)

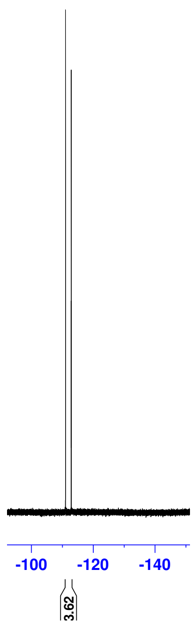

^{13}C NMR (DMSO-d_6)

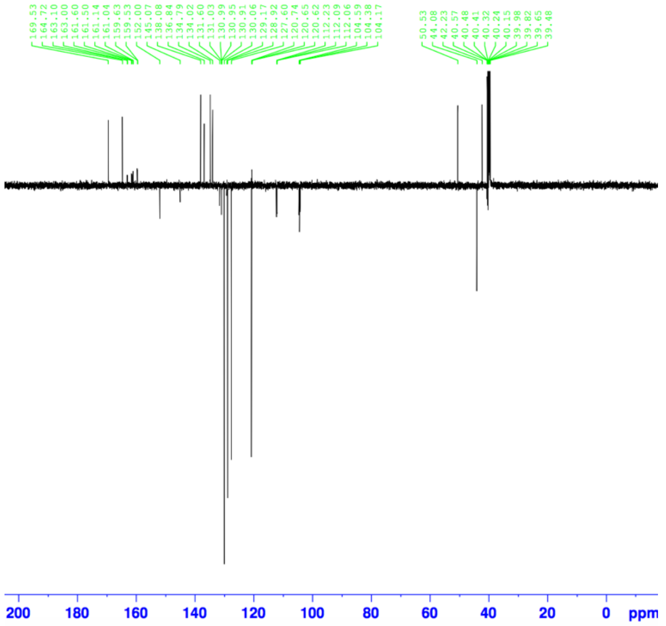

HPLC

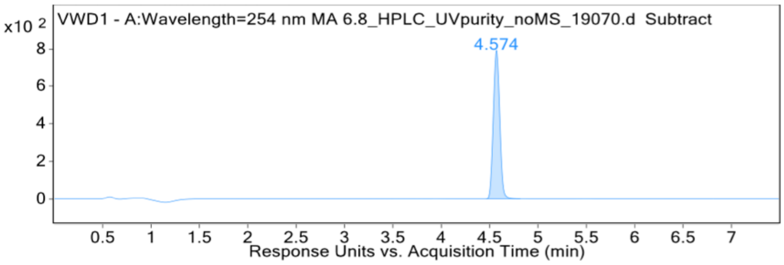

Figure: Base peak or HPLC chromatogram (indicated in left hand corner)

User Chromatogram Peak List

RT (min)	Area	Area %	Area Sum (%)	Symmetry	Width (min)
4.57	3667.95	100.00	100.00	1.07	0.350

Compound 45

^{1}H NMR (DMSO-d_6)

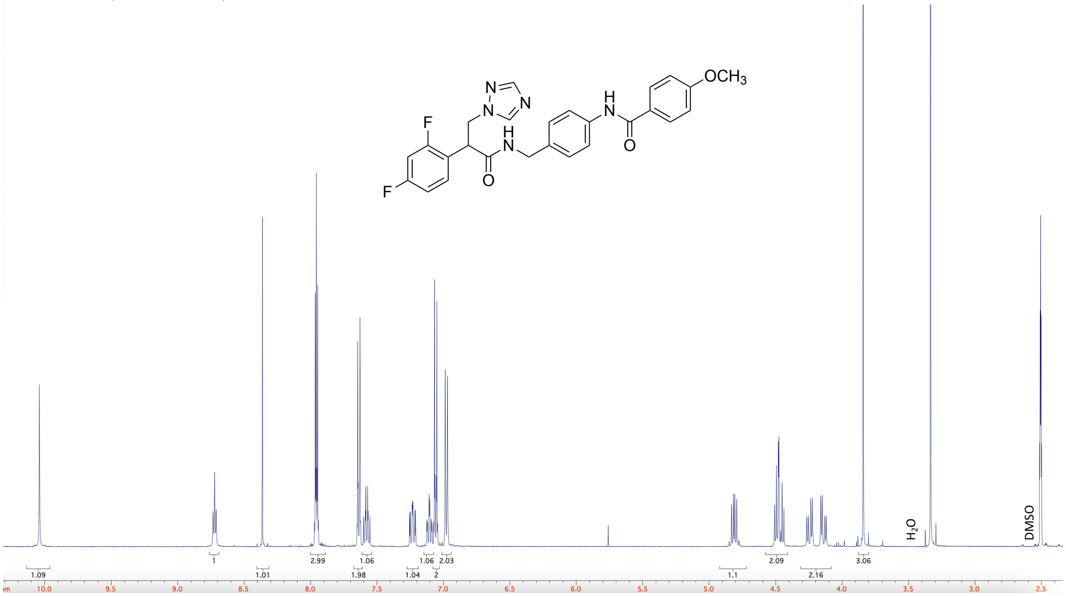

^{19}F NMR (DMSO-d_6)

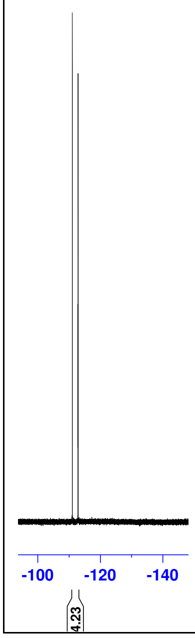

^{13}C NMR (DMSO-d_6)

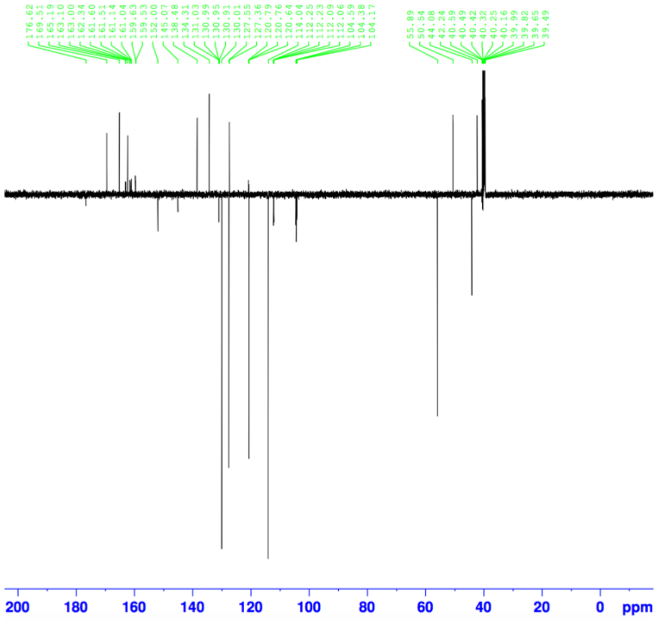

HPLC

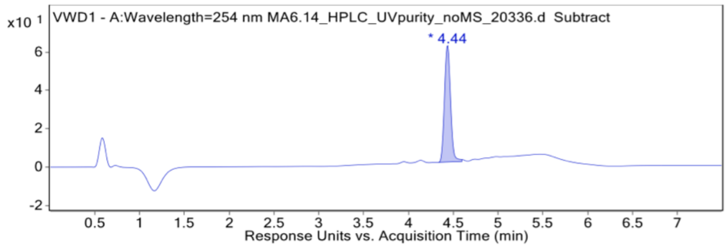

Figure: Base peak or HPLC chromatogram (indicated in left hand corner)

User Chromatogram Peak List

RT (min)	Area	Area %	Area Sum (%)	Symmetry	Width (min)
4.44	286.95	100.00	100.00	1.06	0.260

Compound 46

^{1}H NMR (DMSO-d_6)

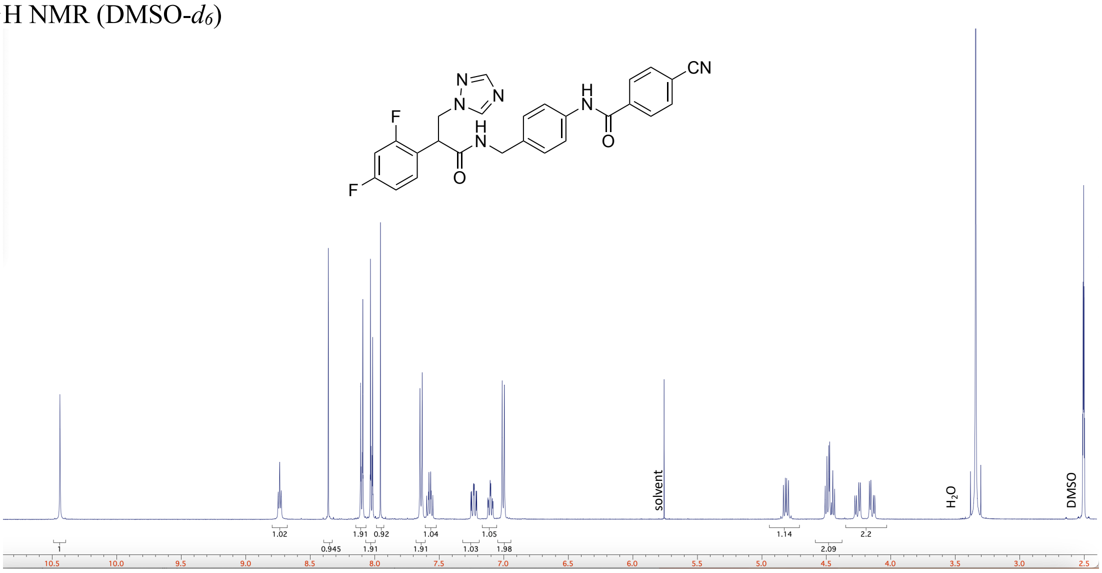

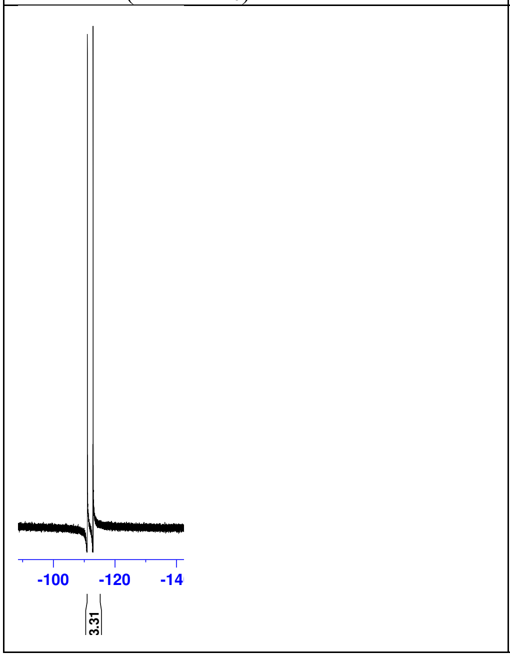

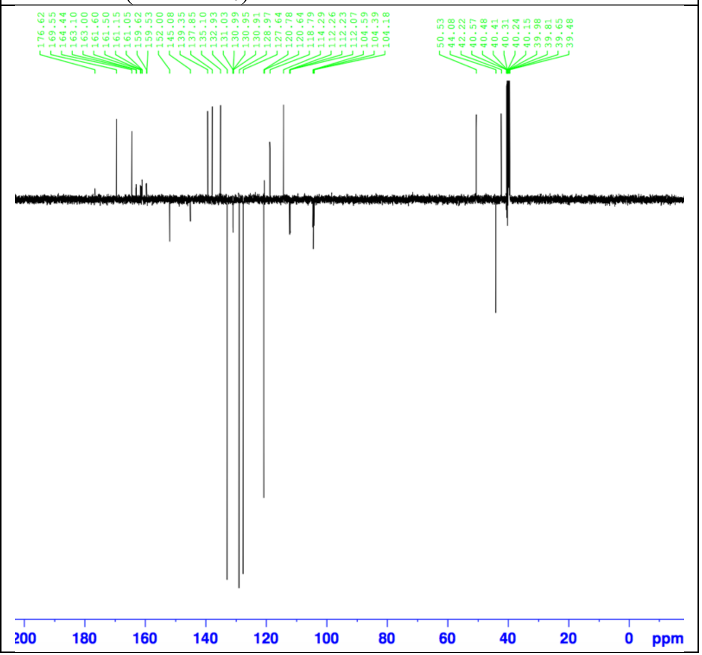

HPLC

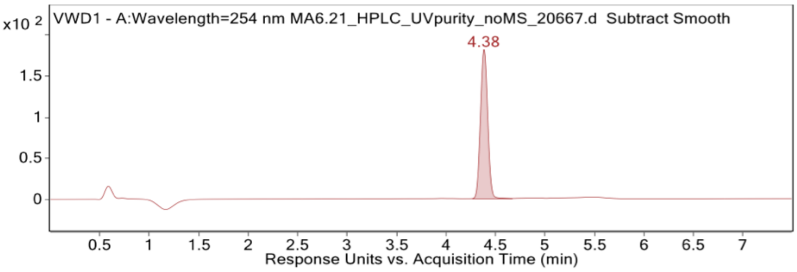

Figure: Base peak or HPLC chromatogram (indicated in left hand corner)

User Chromatogram Peak List

RT (min)	Area	Area %	Area Sum (%)	Symmetry	Width (min)
4.38	923.81	100.00	100.00	1.14	0.400

Compound 47

^{1}H NMR (DMSO-d_6)

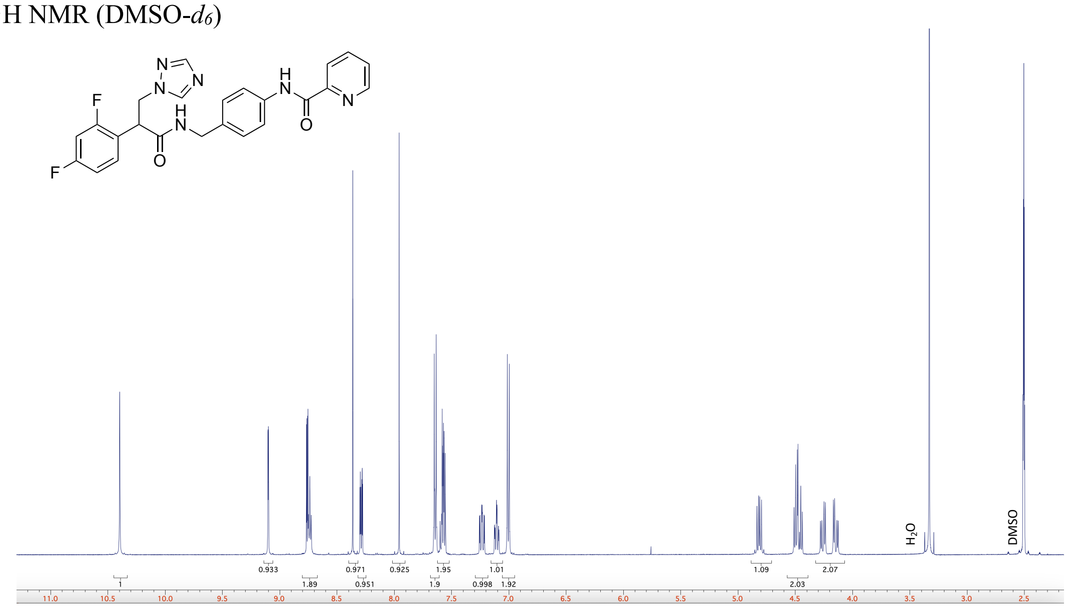

^{19}F NMR (DMSO-d_6)	^{13}C NMR (DMSO-d_6)

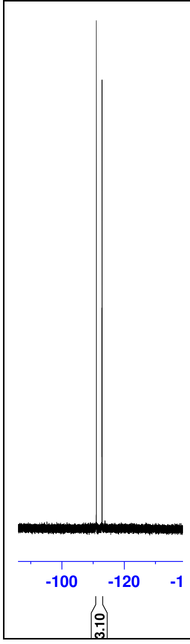

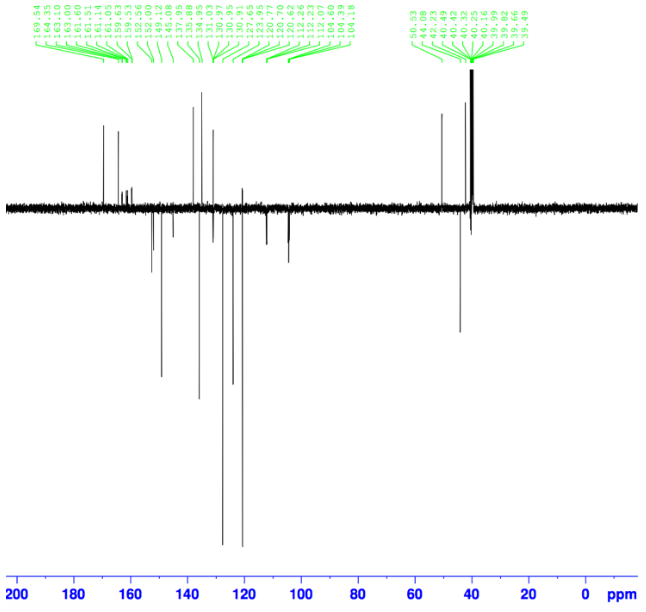

HPLC

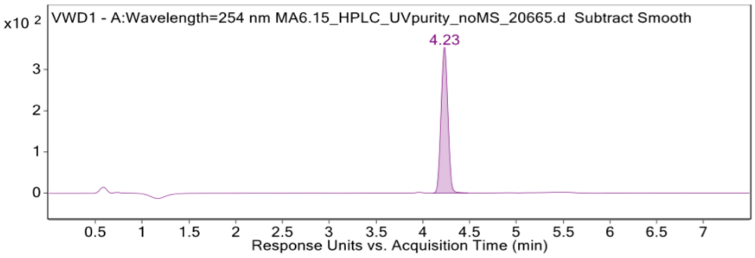

Figure: Base peak or HPLC chromatogram (indicated in left hand corner)

User Chromatogram Peak List

RT (min)	Area	Area %	Area Sum (%)	Symmetry	Width (min)
4.23	1791.72	100.00	100.00	0.96	0.370

Compound 48

^{1}H NMR (DMSO-d_6)

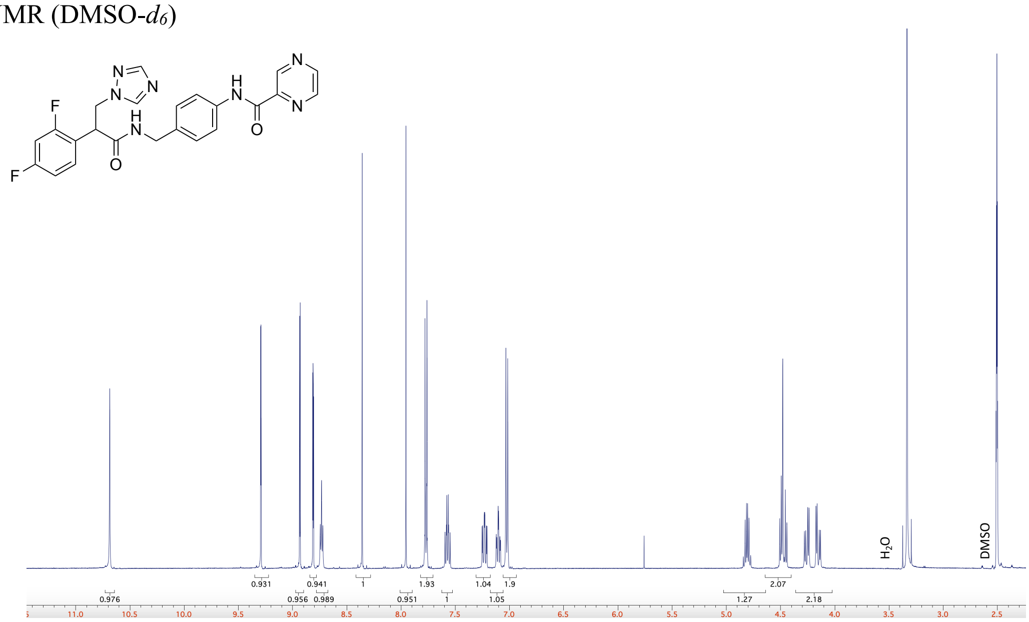

^{19}F NMR (DMSO-d_6)

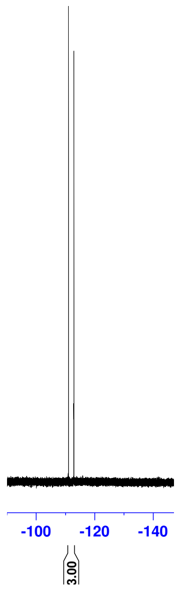

^{13}C NMR (DMSO-d_6)

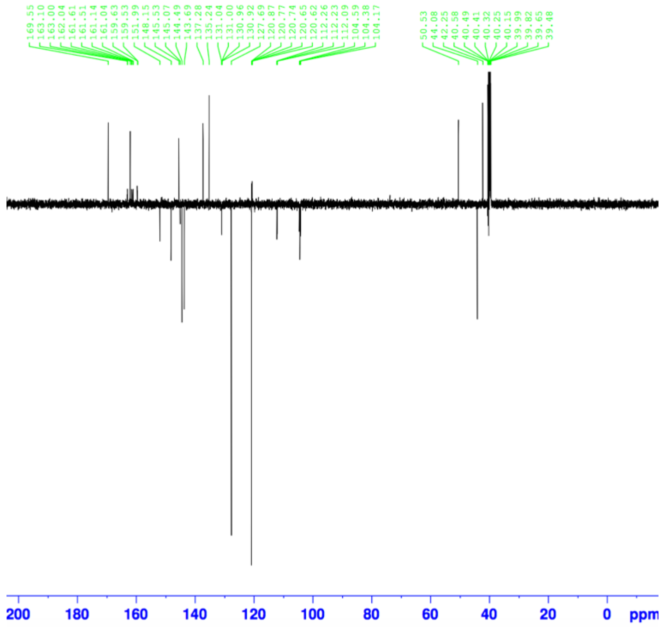

HPLC

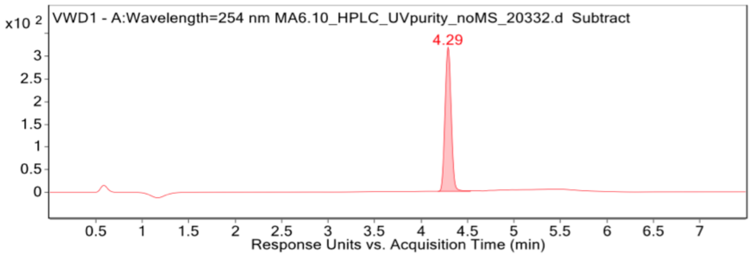

Figure: Base peak or HPLC chromatogram (indicated in left hand corner)

User Chromatogram Peak List

RT (min)	Area	Area %	Area Sum (%)	Symmetry	Width (min)
4.29	1468.98	100.00	100.00	1.1	0.350

References

1. Decottignies, A.; Grant, A.M.; Nichols, J.W.; de Wet, H.; McIntosh, D.B.; Goffeau, A. ATPase and multidrug transport activities of the overexpressed yeast ABC protein Yor1p. *J. Biol. Chem*., **1998**, *273*, 12612-12622. doi: 10.1074/jbc.273.20.12612

2. Lamping, E.; Monk, B.C.; Niimi, K.; Holmes, A.R.; Tsao, S.; Tanabe, K.; Nimii, M.; Uehara, Y.; Cannon, R.D. Characterization of three classes of membrane proteins involved in fungal azole resistance by functional hyperexpression in *Saccharomyces cerevisiae*. *Eukaryot. Cell*, **2007**, *6*, 1150-1165. doi: 10.1128/EC.00091-07.

3. Sagatova, A.A.; Keniya, M.V.; Wilson, R.K.; Monk, B.C.; Tyndall, J.D.A. Structural insights into binding of the antifungal drug fluconazole to *Saccharomyces cerevisiae* lanosterol 14α-demethylase. *Antimicrob. Agents Chemother*., **2015**, *59*, 4982-4989. doi: 10.1128/AAC.00925-15

4. Sagatova, A.A.; Keniya, M.V.; Wilson, R.K.; Sabherwal, M.; Tyndall, J.D.A.; Monk, B.C. Triazole resistance mediated by mutations of a conserved active site tyrosine in fungal lanosterol 14α-demethylase**.** *Sci. Rep*., **2016**, *6*, article No. 26213. doi: 10.1038/srep26213.

5. Graham, D.O.; Wilson, R.K.; Ruma, Y.N.; Keniya, M.V.; Tyndall, J.D.A.; Monk, B.C. Structural insights into the azole resistance of the *Candida albicans* Darlington strain using *Saccharomyces cerevisiae* lanosterol 14α-demethylase as a surrogate. *J. Fungi*, **2021**, *7*, 897. doi:10.3390/jof7110897.

6. Keniya, M.V.; Ruma, Y.N.; Tyndall, J.D.A.; Monk, B.C. Heterologous expression of full-length lanosterol 14α-demethylases of prominent fungal pathogens *Candida albicans* and *Candida glabrata* provides tools for antifungal discovery. *Antimicrob. Agents Chemother*., **2018**, *62*, e01131-18. doi: 10.1128/AAC.01131-18.

7. Ruma, Y.N.; Keniya, M.V.; Monk, B.C. Exploring *Cryptococcus neoformans* CYP51 and its cognate reductase as a drug target. *J. Fungi* **2022**, *8*, 1256. doi: 10.3390/jof8121256.

8. Ruma, Y.N.; Keniya, M.V.; Tyndall, J.D.A.; Monk, B.C. Characterisation of *Candida parapsilosis* CYP51 as a drug target using *Saccharomyces cerevisiae* as host. *J. Fungi* **2022**, *8*, 69. doi: 10.3390/jof8010069.

9. Toepfer, S.; Lackner, M.; Keniya, M.V.; Monk, B.C. Functional expression of recombinant *Candida auris* proteins in *Saccharomyces cerevisiae* enables azole susceptibility evaluation and drug discovery. *J. Fungi*, **2023**, *9*, 168. doi: 10.3390/jof9020168.

10. Wada, S.; Tanabe, K.; Yamazaki, A.; Niimi, M.; Uehara, Y.; Niimi, K.; Lamping, E.; Cannon, R.D.; Monk, B.C. Phosphorylation of *Candida glabrata* ATP-binding cassette transporter Cdr1p regulates drug efflux activity and ATPase stability. *J. Biol. Chem.*, **2005**, *280*, 94-103. doi: 10.1074/jbc.M408252200.

11. Lackner, M.; Keniya, M.V.; Lax, C.; Toepfer, S.; Cesarini, L.; Zenz, L-M.; Müller, C.; Molina, F.E.N; Binder, U.; Bracher, F.; Monk, B.C. The molecular basis of intrinsic resistance to voriconazole in mucormycetes. Unpublished.

12. Hosseini, P.; Keniya1, M.V.; Sagatova, A.A.; Toepfer, S.; Müller, C.; Tyndall, J.D.A.; Klinger, A.; Fleischer, E.; Monk, B.C. The molecular basis of the intrinsic and acquired resistance to azole antifungals in *Aspergillus fumigatus*. *J. Fungi*, **2024**, *10*, 820. doi: 10.3390/jof10120820

13. van het Hoog, M.; Rast, T.J.; Martchenko, M.; Grindle, S.; Dignard, D.; Hogues, H.; Cuomo, C.; Berriman, M.; Scherer, S.; Magee, B.B.; Whiteway, M.; Chibana, H.; Nantel, A.; Magee, P.T. Assembly of the *Candida albicans* genome into sixteen supercontigs aligned on the eight chromosomes. *Genome Biol.*, **2007**, *8*, R52. doi: 10.1186/gb-2007-8-4-r52

14. Marr, K.A.; Lyons, C.N.; Ha, K.; Rustad, T.R.; White, T.C. Inducible azole *resistance associated with a heterogeneous phenotype in Candida albicans. Antimicrob*. Agents Chemother., **2001**, *45*, 52–59. doi: 10.1128/AAC.45.1.52-59.2001.

15. Skrzypek, M.S.; Binkley, J.; Binkley, G.; Miyasato, S.R.; Simison, M.; Sherlock, G. The Candida Genome Database (CGD): incorporation of Assembly 22, systematic identifiers and visualization of high throughput sequencing data. *Nucleic Acids Res.*, **2017**, *45*, D592-D596. doi: 10.1093/nar/gkw924

16.Hofmayer, M.S.; Lutter, F.H.; Grokenberger, L.; Hammann, J.M.; Knochel, P. Practical Ni-catalyzed cross-coupling of unsaturated zinc pivalates with unsaturated nonaflates and triflates. *Org. Lett.,* **2019**, *21*, 36-39. https://doi.org/10.1021/acs.orglett.8b03417

17. Hargrove, T.Y.; Friggeri, L.; Wawrzak, Z.; Qi, A.; Hoekstra, W.J.; Schotzinger, R.J.; York, J.D.; Guengerich, F.P.; Lepesheva, G.I. Structural analyses of *Candida albicans* sterol 14α-demethylase complexed with azole drugs address the molecular basis of azole-mediated inhibition of fungal sterol biosynthesis. *J. Biol. Chem.*, **2017**, *292*, 6728-6743. doi: 10.1074/jbc.M117.778308.

18. Molecular Operating Environment (MOE 2022); Chemical Computing Group Inc.: Montreal, QC, Canada, 2022. Available online: https://www.chemcomp.com
19. Desmond Schrödinger Release 2020-1 Available online: https://www.schrodinger.com/products/desmond.
20. Bowers, K.J.; Chow, D.E.; Xu, H.; Dror, R.O.; Eastwood, M.P.; Gregersen, B.A.; Klepeis, J.L.; Kolossvary, I.; Moraes, M.A.; Sacerdoti, F.D. Scalable algorithms for molecular dynamics simulations on commodity clusters. In Proceedings of the SC'06: Proceedings of the 2006 ACM/IEEE Conference on Supercomputing; IEEE, 2006; p. 43.
